# Actinomycetoquinones A–E, Anthraquinone-γ-Pyrones Discovered from Marine-Derived *Actinomycetospora* sp. Bacterium

**DOI:** 10.1021/acs.jnatprod.5c00578

**Published:** 2025-09-25

**Authors:** Fan Zhang, Christopher D. Roberts, Tae Hyun Lee, Doug R. Braun, Zachary D Bennett, Shaurya Chanana, Song Guo, Gene E. Ananiev, Ilia A. Guzei, Scott R. Rajski, Thomas C. Brunold, Tim S. Bugni

**Affiliations:** † Pharmaceutical Sciences Division, 5228 University of Wisconsin−Madison, Madison, Wisconsin 53706, United States; ‡ Small Molecule Screening Facility, UW Carbone Cancer Center, Madison, Wisconsin 53706, United States; § Department of Chemistry, University of Wisconsin−Madison, Madison, Wisconsin 53706, United States; ∥ Lachman Institute for Pharmaceutical Development, 5228 University of Wisconsin−Madison, Madison, 53706 Wisconsin, United States

## Abstract

Facilitated by LC–MS–PCA metabolomics methods and effective molecular networking for strain prioritization and dereplication, five new anthraquinone-γ-pyrones, actinomycetoquinones A–E (**1**–**5**), were isolated from a marine-derived *Actinomycetospora* sp. bacterium. The structures of **1**–**5** were elucidated by analysis of their HRMS and NMR spectroscopic data. The absolute configuration of **1** was unequivocally determined by single-crystal X-ray diffraction analysis using Cu Kα radiation. Actinomycetoquinone C exhibited antibacterial activity against methicillin-resistant *S. aureus* (MRSA) and *E. coli* with MIC values of 1 and 4 μg/mL, respectively.

## Graphical abstract

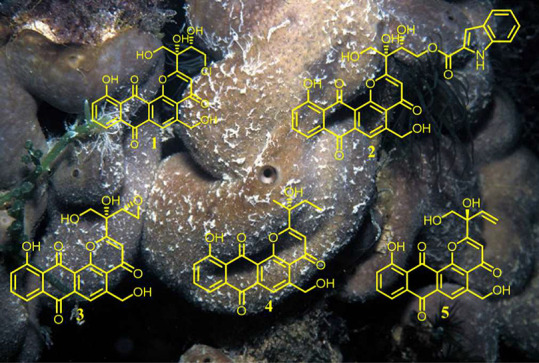

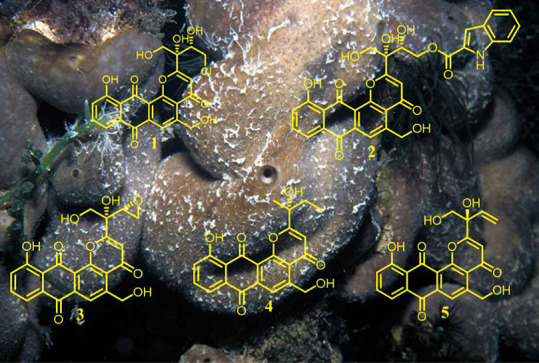

The emergence of multidrug-resistant microbial pathogens presents humanity with ever evolving challenges and represents one of the most serious global public health threats. In the United States, it has been estimated that each year antibiotic-resistant bacteria and fungi cause over 3 million infections with bacterial pathogens accounting for the majority.^1^ Additionally, Gram-negative bacterial pathogens have emerged that are pan-resistant.^2^ This growing disease burden underscores the need for new antibacterial agents.

Actinomycetes, particularly *Streptomyces* species, have been one of the most prolific sources for the discovery of new antibiotics, and nearly 80% of the clinical antibiotic pharmacophores can be traced to *Streptomyces*.^3^ However, more recently, attention has been diverted to non-*Streptomyces* actinomycetes to lessen the high rediscovery rate of known compounds from *Streptomyces*.^4^ Studies on actinomycetes from marine habitats have led to the discovery of new genera including some that are obligate marine species such as *Salinospora*;^500^ this supports the notion that the marine environment is a rich source for understudied taxa and has high potential for the discovery of new scaffolds. Furthermore, the proven track record of marine-derived rare actinomycetes indicates that they are prolific producers of diverse chemicals, structurally unique secondary metabolites, and novel therapeutic compounds.^5^ The genus *Actinomycetospora* was first described by Jiang et al. in 2008 ^6^ and emended by Tamura et al. in 2011.^7^ Only two examples of new molecules have been isolated from *Actinomycetospora*: they include thiasporine A, the first natural product featuring a 5-hydroxy-4*H*-1,3-thiazin-4-one moiety, along with two novel thiazole derivatives, thiasporines B and C, derived from *A. chlora*.^8^ Additionally, mycetoindole, a *N*-acyl dehydrotryptophan compound, was isolated from *Actinomycetospora* sp. RD054979.^9^ The scarcity of new molecules from *Actinomycetospora* highlights the underexplored nature of this genus.

In our efforts to explore rare actinomycetes as sources of new antibiotics, 1667 microbial crude extracts were screened against methicillin-resistant *Staphylococcus aureus* (MRSA), *Escherichia coli*, and *Candida albicans*. Of these organisms, strain *Actinomycetospora* sp. WMMD-1143 displayed growth inhibition against *E. coli* and MRSA. In parallel, metabolomics showed that WMMD-1143 was a metabolic outlier using LC–MS–PCA.^10,11^ Therefore, we investigated this strain in greater detail using molecular networking (GNPS; http://gnps.ucsd.edu).^12^ The LC–MS/MS-based molecular network from GNPS helped to uncover the relationships involving a class of unknown antibiotics from WMMD-1143 (Figure 1). Importantly, leveraging the MS data for targeted isolation enabled us to discover five new antibacterial anthraquinone-γ-pyrones, actinomycetoquinones A–E (Figure 2A).

**Figure 1:**
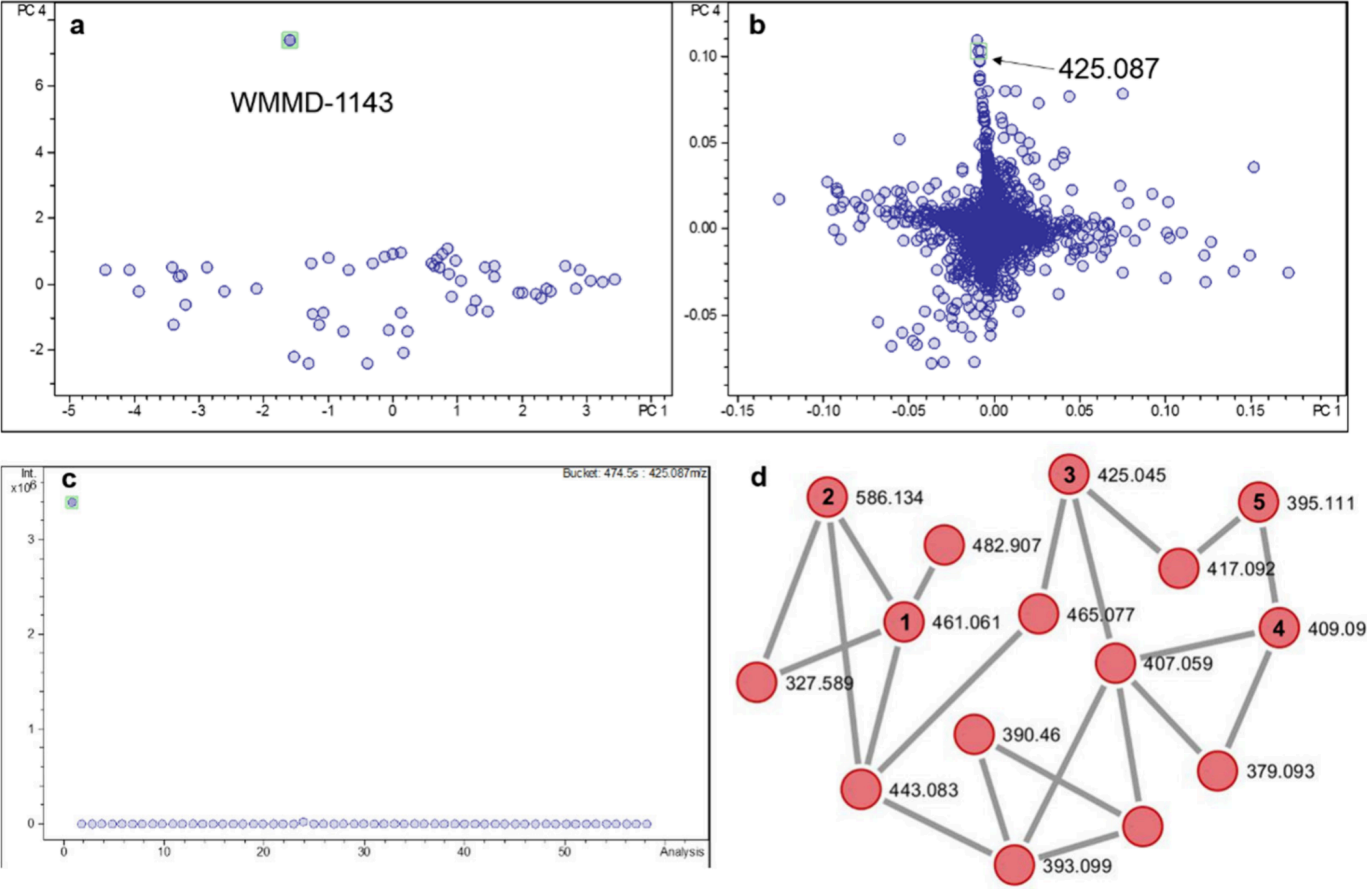
PCA scores and loading plots and molecular networking for strain WMMD-1143. (a) The PCA score plot for PC1 vs PC4 shows that WMMD-1143 is separate from the other strains. (b) The loadings plot shows molecular features unique to WMMD-1143. (c) The bucket statistics plot indicates that the compound at *m*/*z* 425.087 ([M + H]^+^) is unique to strain WMMD-1143. (d) Cluster of anthraquinone-γ-pyrone-derived analogues generated by GNPS.

**Figure 2:**
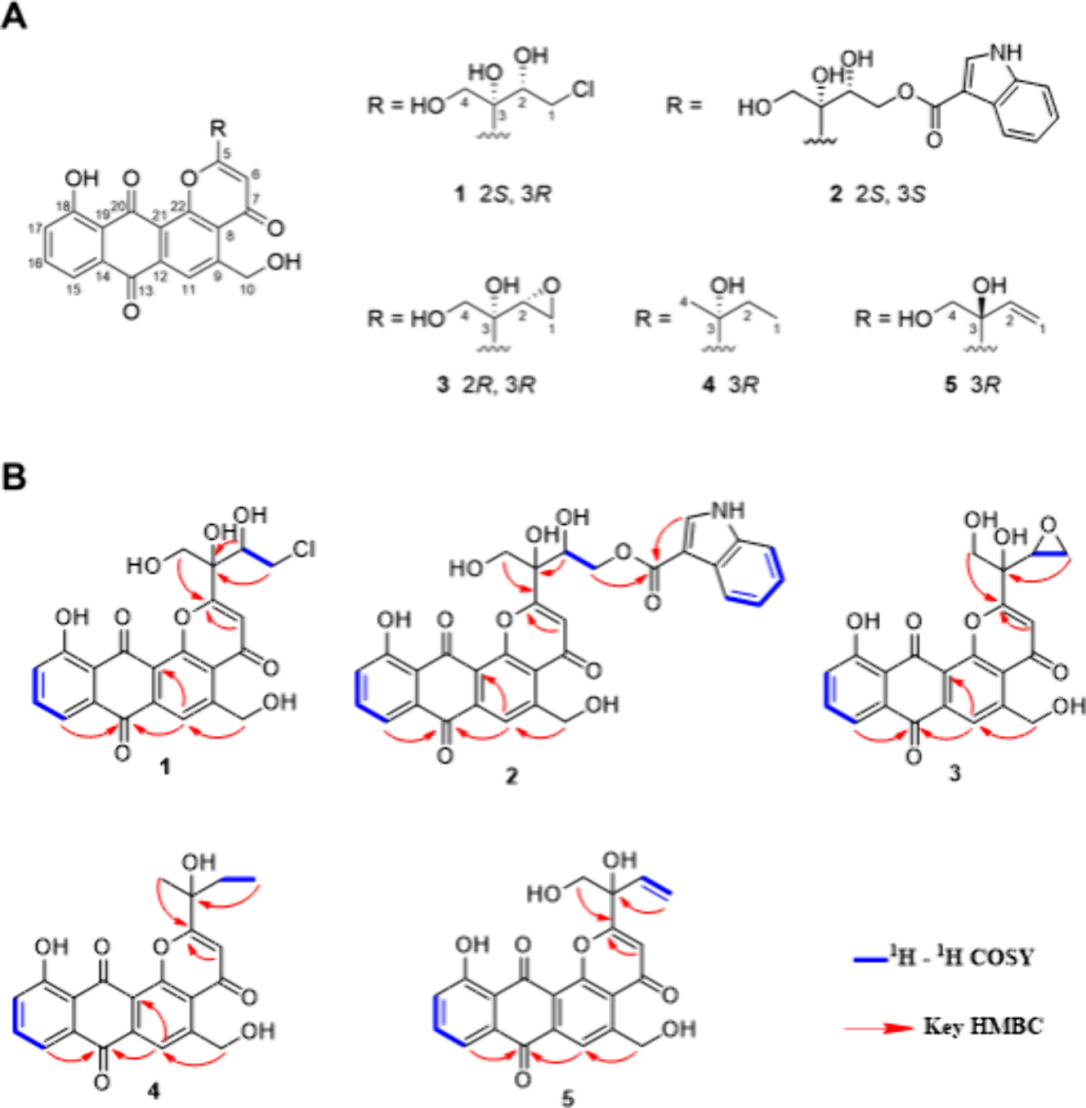
Structural determination of actinomycetoquinones A–E (**1**–**5**). (A) Chemical structures of actinomycetoquinones A–E (**1**–**5**) isolated from WMMD-1143. (B) Key COSY (blue bold) and HMBC (red arrows) correlations of **1**–**5**.

## Results and Discussion

Actinomycetoquinone A (**1**) was isolated as yellow needles with the molecular formula C_22_H_17_ClO_9_ on the basis of the protonated molecular ion at *m*/*z* 461.0631 [M + H]^+^ (calcd for C_22_H_18_ClO_9_, 461.0634, error = 0.65 ppm) in the HRESIMS. The ^1^H and ^13^C NMR showed characteristic signals corresponding to one anthraquinone-γ-pyrone moiety [δ_H_ 8.61 (1H, s, H-11), 7.78 (1H, d, *J* = 7.9 Hz, H-15), 7.69 (1H, d, *J* = 7.9 Hz, H-16), 7.32 (1H, d, *J* = 7.9 Hz, H-17), 6.78 (1H, s, H-6), and 5.27 (2H, s, H-10); δ_C_ 188.0 (C-20), 182.5 (C-13), 180.5 (C-7), 170.9 (C-5), 163.1 (C-18), 156.6 (C-22), 153.5 (C-9), 137.6 (C-16), 137.4 (C-12), 133.1 (C-14), 125.9 (C-17), 125.8 (C-8), 121.4 (C-21), 121.2 (C-11), 120.2 (C-15), 117.5 (C-19), 112.8 (C-6), and 64.2 (C-10)], which was identified on the basis of comparisons to previously reported data for saliniquinone G,^13^ along with one oxygenated methine [δ_H_ 4.48 (1H, dd, *J* = 8.3 and 3.7 Hz, H-2); δ_C_ 74.5 (C-2)], one oxygenated methylene [δ_H_ 4.31 (1H, d, *J* = 12.0 Hz, H-4a) and 4.13 (1H, d, *J* = 12.0 Hz, H-4b); δ_C_ 66.5 (C-4)], and one oxygenated tertiary carbon [δ_C_ 79.4 (C-3)] (Table 1). Furthermore, a deshielded shift of the methylene [δ_H_ = 3.64 (1H, *J* = 12.0 and 3.7 Hz, H-1a) and 3.60 (1H, *J* = 12.0 and 8.3 Hz, H-1b); δ_C_ = 46.0 (C-1)] supported the placement of the Cl attached to C-1. The planar structure was determined based on the ^1^H–^1^H COSY, HSQC, and HMBC analysis. The HMBC correlations of H_2_-1 (δ_H_ 3.64 and 3.60), H_2_-4 (δ_H_ 4.31 and 4.13), and H-6 (δ_H_ 6.78) with C-3 (δ_C_ 79.4) as well as the correlations of H-2 (δ_H_ 4.48), H_2_-4 (δ_H_ 4.31 and 4.13), and H-6 (δ_H_ 6.78) with C-5 (δ_C_ 170.9) indicated that the linear structure (C-1 to C-4) is connected at C-5 of the anthraquinone-γ-pyrone moiety (Figure 2B). Importantly, the absolute configuration of **1** was determined by single-crystal X-ray diffraction, which established the configuration as 2*S*,3*R*, shown in the ORTEP diagram (Figure 3A).

**Table 1:** ^1^H (600 MHz) and ^13^C (150 MHz) NMR Spectroscopic Data for Compounds **1**–**5** (*δ* in ppm, *J* values in Hz)

	**1** ^a^		**2** ^a^		**3** ^b^		**4** ^a^		**5** ^a^	
Position	δ_C_	δ_H_ (*J* in Hz)	δ_C_	δ_H_ (*J* in Hz)	δ_C_	δ_H_ (*J* in Hz)	δ_C_	δ_H_ (*J* in Hz)	δ_C_	δ_H_ (*J* in Hz)
1a	46.0	3.64, dd (12.0, 3.7)	62.9	4.33, t (10.0)	42.0	2.87, dd (5.5, 2.5)	8.3	0.92, t (7.4)	117.4	5.66, d (17.0)
1b		3.60, dd (12.0, 8.3)		4.21, dd (10.0, 3.3)		2.73, dd (5.5, 4.1)				5.36, d (11.0)
2a	74.5	4.48, dd (8.3, 3.7)	70.8	4.69, dd (10.0, 3.3)	53.4	3.66, dd (4.1, 2.5)	34.1	2.16, m	137.4	6.40, dd (17.0, 11.0)
2b								1.96, m		
3	79.4		78.7		74.4		74.4		78.4	
4a	66.5	4.31, d (12.0)	67.1	4.08, d (12.0)	65.3	4.22, dd (11.0, 6.0)	26.9	1.70, s	68.3	4.36, d (12.0)
4b		4.13, d (12.0)		4.00, d (12.0)		3.84, dd (11.0, 6.0)				3.80, d (12.0)
5	170.9		171.2		169.4		176.1		172.4	
6	112.8	6.78, s	112.1	6.64, s	111.2	6.53, s	109.8	6.68, s	111.0	6.74, s
7	180.5		180.8		178.2		181.1		181.0	
8	125.8		125.5		124.7		125.6		125.8	
9	153.5		151.3		153.4		153.3		153.5	
10a	64.2	5.27, s	64.1	4.89, d (17.0)	62.3	5.15, br s	64.3	5.27, s	64.3	5.27, s
10b				4.81, d (17.0)						
11	121.2	8.61, s	120.7	7.86, s	118.8	8.50, s	121.3	8.61, s	121.3	8.62, s
12	137.4		136.3		136.0		137.7		137.7	
13	182.5		182.2		181.4		182.6		182.6	
14	133.1		132.9		132.1		133.1		133.2	
15	120.2	7.78, d (7.9)	119.8	7.49, d (8.0)	118.8	7.68, d (7.5)	120.1	7.78, d (7.9)	120.2	7.80, d (7.9)
16	137.6	7.69, t (7.9)	137.0	7.50, t (8.0)	136.7	7.76, t (7.5)	137.3	7.70, t (7.9)	137.4	7.70, t (7.9)
17	125.9	7.32, d (7.9)	125.6	7.16, d (8.0)	124.1	7.38, d (7.5)	125.9	7.34, d (7.9)	125.9	7.36, d (7.9)
18	163.1		162.9		161.4		163.1		163.2	
19	117.5		117.4		116.7		117.5		117.5	
20	188.0		187.4		186.9		187.8		188.0	
21	121.4		120.4		119.8		121.3		121.3	
22	156.6		156.2		155.2		156.8		156.9	
2′			132.2	6.98, s						
3′			107.6							
4′			121.0	7.26, d (8.0)						
5′			122.1	6.32, t (8.0)						
6′			123.6	6.56, t (8.0)						
7′			112.5	6.82, d (8.0)						
8′			136.6							
9′			125.2							
10′			165.6							

a CD_3_OD:CDCl_3_ (1:1).

b DMSO-*d*_*6*_.

**Figure 3:**
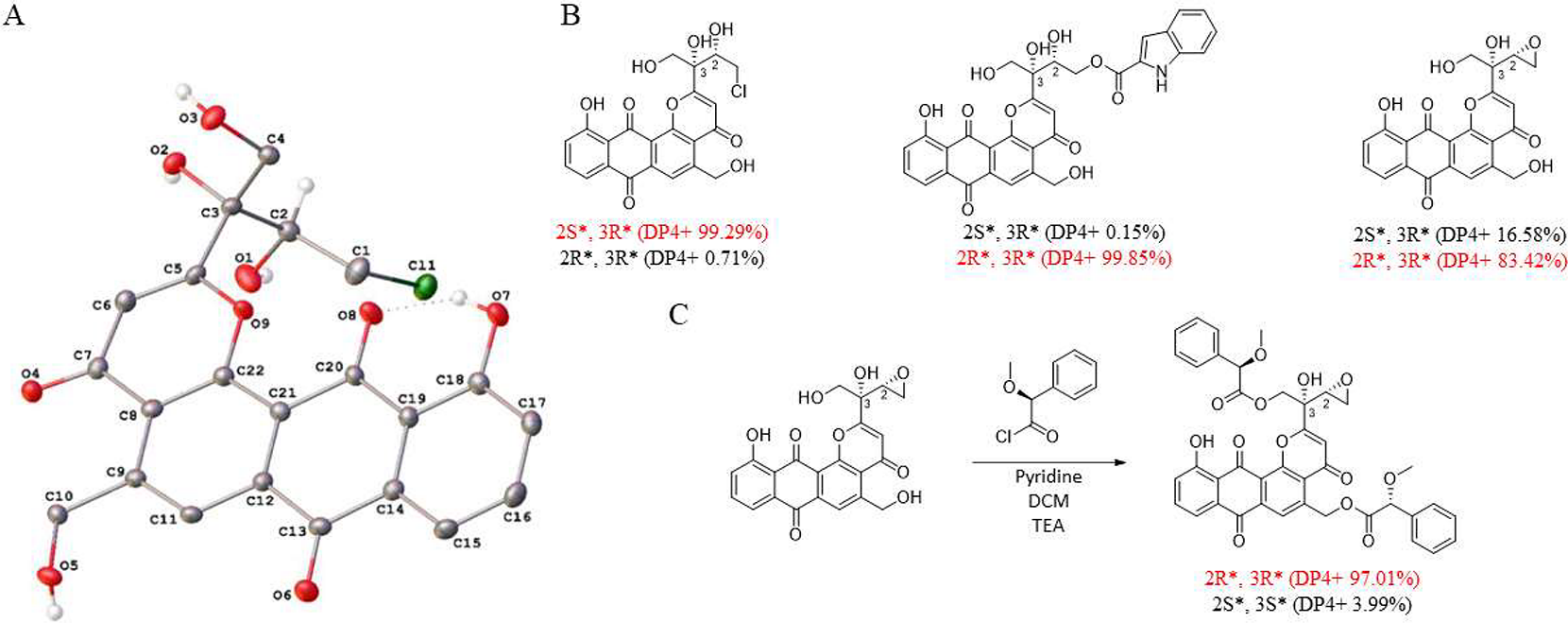
Configurational analysis of **1**–**3**. (A) A molecular drawing of **1** shown with 50% atomic displacement ellipsoids. (B) From left to right, the DP4+ probability analysis of **1**–**3**. (C) Esterification of the alcohol groups in **3** and DP4+ probability analysis comparing calculated NMR data with experimental values.

Actinomycetoquinone B (**2**) was obtained as a yellow powder, and its molecular formula was determined as C_31_H_23_N_1_O_11_ based on the protonated molecular ion at *m*/*z* 586.1333 [M + H]^+^ (calcd for C_31_H_24_NO_11_, 586.1344, error = 1.87 ppm) in the HRESIMS. The ^1^H and ^13^C NMR of **2** were comparable to those of **1**, with the major difference being the presence of an indole-3-carboxylate moiety [δ_H_ 7.26 (1H, d, *J* = 8.0 Hz, H-4′), 6.98 (1H, s, H-2′), 6.82 (1H, d, *J* = 8.0 Hz, H-7′), 6.56 (1H, t, *J* = 8.0 Hz, H-6′), and 6.32 (1H, t, *J* = 8.0 Hz, H-5′); δ_C_ 165.6 (C-10′), 136.6 (C-8′), 132.2 (C-2′), 125.2 (C-9′), 123.6 (C-6′), 122.1 (C-5′), 121.0 (C-4′), 112.5 (C-7′), and 107.6 (C-3′)] (Table 1). The detailed ^1^H and ^13^C NMR analysis revealed that **2** possessed a core scaffold similar to that of **1**; this was further confirmed by the ESI–MS/MS data of **2** (Figure S14, Supporting Information). ESI–MS/MS data revealed a fragment ion at *m*/*z* 443.0958, roughly equivalent to the molecular weight for C_22_H_19_O_10_ (calcd for C_22_H_19_O_10_, 443.0973, error = 3.38 ppm), whereas the downfield-shifted signals for H_2_-1 (δ_H_ 4.33 and 4.21) and C-1 (δ_C_ 62.9) suggested that the C-1 chlorine atom of **1** was replaced by an oxygen atom in **2**. In addition, the molecular formula of the remaining partial structure was calculated as C_9_H_7_N_1_O_2_, on the basis of the molecular weight of **2** and the fragment ion at *m*/*z* 443.0958, suggesting the presence of the indole-3-carboxylic acid moiety (Figure S14, Supporting Information). The indole-3-carboxylate moiety was theorized to be linked at C-1 on the basis of the downfield-shifted chemical shifts for **2** (δ_H‑1_ 4.33 and 4.21; δ_C‑1_ 62.9) compared to those of **1** (δ_H‑1_ 3.64 and 3.60; δ_C‑1_ 46.0), which was verified by an HMBC cross-peak between H_2_-1 (δ_H‑1_ 4.33 and 4.21) and C-10′ (δ_C_ 165.6) (Figure 2B). To determine the relative configuration at positions 2 and 3, experimental NMR shifts were compared to calculated NMR shifts of the possible diastereomers (2R*,3R*) and (2S*,3R*). A library search of conformers was done at the molecular mechanics level, and these conformers were further optimized using density functional theory (DFT) calculations. (2*S*,3*R*)-**2** resulted in two conformers below the set energy cutoff (10 kJ/mol), while (2*R*,3*R*)-**2** resulted in three stable conformers. The conformer NMR shifts for each diastereomer were averaged with the Boltzmann distribution theory. Using DP4+ to compare the calculated and experimental ^13^C shifts, the DP4+ probability analyses of (2*S*,3*R*)-**2** and (2*R*,3*R*)-**2** suggested **2** to be 2R*,3R* with 99.85% probability (Figure 3B). To elucidate the absolute configuration, (2*R*,3*R*)-**2** and (2*S*,3*S*)-**2** underwent time dependent density functional theory (TDDFT) calculations using Gaussian 16 to generate the calculated ECD spectrum for each enantiomer. Two conformers for (2*R*,3*R*)-**2** lay below the energy cutoff (10 kJ/mol), while 14 conformers for (2*S*,3*S*)-**2** lay below that cutoff. The calculated ECD spectrum of each enantiomer was generated by averaging ECD spectra using the Boltzmann distribution theory. The absolute configuration of **2** was elucidated by comparisons of the calculated ECD spectra of (2*R*,3*R*)-**2** and (2*S*,3*S*)-**2** with the experimental ECD spectrum of **2**. The experimental ECD data of **2** closely matched the calculated ECD data of (2*S*,3*S*)-**2** (Figure S15, Supporting Information).

Actinomycetoquinone C (**3**) was purified as a yellow powder. The molecular formula of **3** was confirmed to be C_22_H_16_O_9_ based on the protonated molecular ion at *m*/*z* 425.0871 [M + H]^+^ (calcd for C_22_H_17_O_9_, 425.0867, error = 0.94 ppm) in the HRESIMS. The ^1^H and ^13^C NMR of **3** were quite similar to those of **1**, except for resonances indicating the presence of a terminal epoxy ring signal [δ_H_ 3.66 (1H, dd, *J* = 4.1 and 2.5 Hz, H-2), 2.87 (1H, dd, *J* = 5.5 and 2.5 Hz, H-1a), and 2.73 (1H, dd, *J* = 5.5 and 4.1 Hz, H-1b); δ_C_ 53.4 (C-2) and 42.0 (C-1)] (Table 1), which was identified by comparison with previously reported values.^14–17^ The position/connectivity of the distinctive partial structure was assigned at C-2 based on the upfield-shifted proton and carbon chemical values of **3** (δ_H‑1a_ 2.87, δ_H‑1b_ 2.73, and δ_H‑2_ 3.66; δ_C‑1_ 42.0 and δ_C‑2_ 53.4) compared to those of **1** (δ_H‑1a_ 3.64, δ_H‑1b_ 3.60, and δ_H‑2_ 4.48; δ_C‑1_ 46.0 and δ_C‑2_ 74.5) (Table 1). This logic was validated by HMBC correlations involving H-2 (δ_H_ 3.66) and H-6 (δ_H_ 6.53) with C-3 (δ_C_ 74.4) as well as COSY cross-peaks between H_2_-1 (δ_H‑1a_ 2.87 and δ_H‑1b_ 2.73) and H-2 (δ_H_ 3.66) (Figure 2B). The calculated ^13^C NMR chemical shifts of (2S*,3R*)-**3** and (2R*,3R*)-**3** were compared with the experimental NMR data of **3** using DP4+ analyses to confirm the relative configuration using the same methodology as **2**. (2*R*,3*R*)-**3** resulted in eight low energy conformers, while (2*S*,3*R*)-**3** resulted in nine low energy conformers. (2*R*,3*R*)-**3** showed a DP4+ probability score of 83.42%, which indicates the relative configuration must be (2R*,3R*)-**3** (Figure 3B). The absolute configuration of **3** was assigned as 2*R*,3*R* based on comparisons of the calculated ECD spectra of (2*S*,3*S*)-**3** and (2*R*,3*R*)-**3** with the experimental ECD spectrum of **3** (Figure S22, Supporting Information). Due to the low signal of the ECD spectrum, another approach was used to confirm the absolute configuration. Esterification of the primary alcohol in compound **3** with *R*-meth­oxy­phenyl­acetyl chloride enabled differentiation of the two possible enantiomers by NMR. The resulting ^13^C NMR spectrum was compared to calculated ^13^C shifts for both stereoisomers using DP4+ analysis, which indicated (2*R*,3*R*)-**3** as the most probable absolute configuration (Figure 3C).

Actinomycetoquinone D (**4**) was isolated as a yellow powder. Its molecular formula was found to be C_22_H_18_O_7_ based on the protonated molecular ion at *m*/*z* 395.1128 [M + H]^+^ (calcd for C_22_H_19_O_7_, 395.1125, error = 0.75 ppm) and the sodiated molecular ion at *m*/*z* 417.0942 [M + Na]^+^ (calcd for C_22_H_18_NaO_7_, 417.0950, error = 1.91 ppm) in the HRESIMS. Detailed inspection of the ^1^H and ^13^C NMR spectra of **4** suggested this compound to be structurally very similar to **1**, with the major differences being the presence of one methylene [δ_H_ 2.16 (1H, m, H-2a) and 1.96 (1H, m, H-2b); δ_C_ 34.1 (C-2)] and two methyl groups [δ_H_ 1.70 (3H, s, H-4) and 0.92 (3H, t, *J* = 7.4 Hz, H-1); δ_C_ 26.9 (C-4) and 8.3 (C-1)] (Table 1). HMBC correlations from H-1 (δ_H_ 0.92), H_2_-2 (δ_H_ 2.16 and 1.96), H-4 (δ_H_ 1.70), and H-6 (δ_H_ 6.68) to C-3 (δ_C_ 74.4) corroborated the linkage of the methylene and methyl groups at C-3 (Figure 2B). According to previously reported compounds similar to **4**, asymmetric centers were determined by comparing the optical rotation values {[α]_D_^25^ 4.0 (*c* 0.1, DMSO) for *R*-form; [α]_D_^25^ −12 (MeOH) for *S*-form}.^13,18^ Compound **4** showed a positive specific rotation value {[α]_D_^25^ 52.0 (*c* 1, MeOH:CHCl_3_ = 1:1)}, indicating the (*R*)-configuration at C-3.

Actinomycetoquinone E (**5**) was obtained as a yellow powder, and the molecular formula was deduced to be C_22_H_16_O_8_ on the basis of the protonated molecular ion at *m*/*z* 409.0923 [M + H]^+^ (calcd for C_22_H_17_O_8_, 409.0918, error = 1.22 ppm) in the HRESIMS. The ^1^H and ^13^C NMR data sets resembled those of **3**. The major difference was in the presence of an allylic group [δ_H_ 6.40 (1H, dd, *J* = 17.0 and 11.0 Hz, H-2), 5.66 (1H, d, *J* = 17.0 Hz, H-1a), and 5.36 (1H, d, *J* = 11.0 Hz, H-1b); δ_C_ 137.4 (C-2) and 117.4 (C-1)] (Table 1) in **5**, instead of the terminal epoxy ring at C-2 in **3**. The location of the allylic group was elucidated on the basis of HMBC correlations from H-1 (δ_H_ 5.66 and 5.36), H-2 (δ_H_ 6.40), and H-6 (δ_H_ 6.74) to C-3 (δ_C_ 78.4) (Figure 2B). The absolute configuration of the hydroxyl group at C-3 in **5** was determined by ECD analysis and optical rotation. The calculated ECD spectra were generated with the Boltzmann averaged ECD spectra of nine low energy conformers for (3*R*)-**5** and eight low energy conformers for (3*S*)-**5**. The experimental ECD curve of **5** proved to be quite similar to the calculated (*R*)-configuration curve (Figure S36, Supporting Information), and the positive optical rotation value of **5** supported the notion that **5** possesses the (*R*)-configuration at C-3.^18^

Compounds **1**–**5** were tested for antibacterial activity against methicillin-resistant *Staphylococcus aureus* (MRSA) and *Escherichia coli* (Table 2). Compound **3** showed antibacterial activity against MRSA 33591 with an MIC value of 1 μg/mL and against *E. coli* 25922 with an MIC value of 4 μg/mL; compounds **1**, **2**, **4** and **5** all failed to show any detectable antibacterial activities against *E. coli* even at concentrations up to 64 μg/mL. The absence of antimicrobial activity observed for **1**, **2**, **4**, and **5** against *E. coli* suggests that the side chain at C-5 directly impacts antibacterial activity. That compound **3** demonstrated, by a moderately wide margin, the most potent antibacterial activity suggests that the epoxide moiety likely plays a crucial role in dictating the observed biological activities.

**Table 2:** MIC Values (μg/mL) of Actinomycetoquinones A–E (**1**–**5**) against MRSA 33591 and *E. coli* 25922

	**1**	**2**	**3**	**4**	**5**	Vancomycin	Gentamicin
MRSA	2	>64	1	4	8	0.25	N.T.^a^
*E. coli*	>64	>64	4	64	>64	N.T.^a^	4

a N.T.: not tested.

In conclusion, five new anthraquinone-γ-pyrones, actinomycetoquinones A–E (**1**–**5**), were identified from a marine-derived *Actinomycetospora* sp. bacterium. The strain was prioritized based on biological activity and via LC–MS–PCA. Secondary analyses using molecular networking (GNPS) helped identify the family of related compounds. The actinomycetoquinones represent another example of the metabolic diversity of the genus *Actinomycetospora*. Only compound **3** was found to have antibacterial activity albeit against the challenging Gram-negative bacterium *E. coli*. MS-based tools helped identify the bacterial strain as a putative producer of novel antibiotics. Given that the genus is mostly unexplored, *Actinomycetospora* spp. represent a potential source of new molecules.

## Experimental Section

### General Experimental Procedures

Optical rotations were measured on a Perkin-Elmer 241 polarimeter. UV spectra were recorded on an Aminco/OLIS UV–vis spectrophotometer. ECD spectra were recorded on an AVIV Model 420 circular dichroism spectrometer. IR spectra were measured with a Bruker Equinox 55/S FT-IR spectrophotometer. Both 1D and 2D NMR spectra were obtained using a Bruker Avance 500 MHz spectrometer with a ^1^H­{^13^C/^15^N} cryoprobe and a 500 MHz spectrometer with a ^13^C/^15^N­{^1^H} cryoprobe; chemical shifts were referenced to the residual solvent peaks (CD_3_OD: δ_H_ = 3.31, δ_C_ = 49.15; DMSO-*d*_6_: δ_H_ = 2.50, δ_C_ = 39.51). Raw FID data were deposited into the Natural Product Magnetic Resonance Database Project (NP-MRD). HRMS and MS/MS data were acquired with a Bruker MaXis 4G ESI-QTOF mass spectrometer. UHPLC–HRMS and UHPLC–HRMS/MS spectra were acquired using a Bruker MaXis 4G ESI-QTOF mass spectrometer coupled with a Waters Acquity UPLC system operated by Bruker Hystar software and a C_18_ column (Phenomenex Kinetex 2.6 μm, 2.1 × 100 mm). Reversed-phase (RP) HPLC was performed using a Shimadzu Prominence HPLC system and a Phenomenex Gemini C_18_ column (250 × 30 mm, 5 μm) or a Phenomenex Luna C_18_ column (250 × 10 mm, 5 μm).

### Biological Material

WMMD-1143 was isolated from the sponge *Chondrilla nucula* collected in the Florida Keys, USA (24° 41.779′ N, 81° 26.884′ W). It was isolated on Gauze 1 medium containing 50% ASW and supplemented with 50 μg/mL cycloheximide, 25 μg/mL nystatin, and 25 μg/mL nalidixic acid.^19^

### Sequencing

16S rDNA was sequenced as previously described.^20^ WMMD-1143 was identified as *Actinomycetospora* sp., and its 16s rDNA sequence was deposited with GenBank and assigned the accession number MT257077.

### Metabolomics

Bacterial crude extracts were prepared from cultures grown in 24-well plates and analyzed by using our standard LC–MS method. The resulting data were processed using Bruker Compass ProfileAnalysis 2.3 (Billerica, MA, USA). Feature detection was performed with the “Find Molecular Features” algorithm using the following parameters: signal-to-noise (S/N) threshold of 5, correlation coefficient threshold of 0.7, minimum compound length of 10, and smoothing width of 1. LC–MS data were evaluated over a retention time (RT) window of 2 to 14 min and a mass-to-charge (*m*/*z*) range of 150 to 1500. Advanced bucketing was applied with parameters set to ΔRT = 0.33 min and Δ*m*/*z* = 4 ppm. The resulting data sets were analyzed for unique chemical features using the *hcapca*.

### Fermentation, Extraction, and Isolation

Two 10 mL seed cultures (25 × 150 mm tubes) in medium ASW-A (20 g of soluble starch, 10 g of glucose, 5 g of peptone, 5 g of yeast extract, and 5 g of CaCO_3_ per liter of artificial seawater) were inoculated with strain WMMD-1143 and agitated at 200 rpm for 14 days at 28 °C. Artificial seawater was used in the fermentations as described previously.^21^ For an intermediate culture, two 2 L flasks each containing 500 mL of ASW-A were inoculated with 20 mL of seed culture and incubated (200 rpm, 28 °C) for 7 days. Four liter flasks (4) each containing 1 L of Ram2 medium (4 g of corn meal, 10 g of glucose, 15 g of maltose, 7.5 g of pharmamedia, and 5 g of yeast per liter of 50% artificial seawater) with Diaion HP20 (7% by weight) were inoculated with 40 mL from the intermediate culture and shaken at 200 rpm at 28 °C for 14 days. Filtered HP20 was washed with distilled H_2_O and extracted with acetone. The acetone extract (20 g) was subjected to liquid–liquid partitioning using 30% aqueous CH_3_OH and CHCl_3_ (1:1). The CHCl_3_-soluble material (1.5 g) was fractionated using Sephadex LH20 column chromatography (CHCl_3_:CH_3_OH, 1:1, column size 500 × 40 mm, 16 mL for each fraction). The fractions containing **1**–**5** were further subjected to RP preparative HPLC (10–100% CH_3_OH-H_2_O with H_2_O containing 0.1% acetic acid over 25 min, 20.0 mg/mL) using a Shimadzu Prominence HPLC system and a Phenomenex Gemini C_18_ column (250 × 30 mm, 5 μm), yielding **3** (80 mg, *t*_R_ 17.1 min) and **4** (10 mg, *t*_R_ 19.6 min). The HPLC fractions containing **1**, **2**, and **5** were further subjected to RP HPLC (45–80% CH_3_OH-H_2_O with H_2_O containing 0.1% acetic acid over 30 min, 4.0 mg/mL) using a Phenomenex Luna C_18_ column (250 × 10 mm, 5 μm), yielding **1** (10 mg, *t*_R_ 20 min), **2** (2 mg, *t*_R_ 21.1 min), and **5** (5.2 mg, *t*_R_ 22.4 min).

#### Actinomycetoquinone A (**1**)

Yellow needles; mp 255–257 °C; [α]_D_^25^ +14 (*c* 0.5, CH_3_OH:CHCl_3_ 1:1); UV (CH_3_OH:CHCl_3_ 1:1) λ_max_ (log ε) 243 (4.29), 269 (4.05), 423 (3.53) nm; IR (ATR) υ_max_ 3356.2, 2976.1, 1645.29, 1455.6, 1296.3, 1077.1, 1024.6 cm^–1^; ^1^H and ^13^C NMR (see Table 1); HRESIMS *m*/*z* 461.0631 [M + H]^+^ (calcd for C_22_H_18_ClO_9_, 461.0634, error = 0.65 ppm).

#### Actinomycetoquinone B (**2**)

Yellow powder; [α]_D_^25^ +54 (*c* 0.1, CH_3_OH:CHCl_3_ 1:1); UV (CH_3_OH:CHCl_3_ 1:1) λ_max_ (log ε) 242 (4.30), 269 (4.15), 423 (3.54) nm; IR (ATR) υ_max_ 3361.82, 2947.6, 1642.3, 1454.2, 1083.3, 1023.4 cm^–1^; ^1^H and ^13^C NMR (see Table 1); HRESIMS *m*/*z* 586.1333 [M + H]^+^ (calcd for C_31_H_24_NO_11_, 586.1344, error = 1.87 ppm).

#### Actinomycetoquinone C (**3**)

Yellow powder; [α]_D_^25^ +43 (*c* 0.1, CH_3_OH:CHCl_3_ 1:1); UV (CH_3_OH:CHCl_3_ 1:1) λ_max_ (log ε) 243 (4.40), 269 (3.95), 423 (3.70) nm; IR (ATR) υ_max_ 3362.5, 2947.6, 2831.9, 1649.9, 1463.2, 1451.9, 1310.3, 1023.4 cm^–1^; ^1^H and ^13^C NMR (see Table 1); HRESIMS *m*/*z* 425.0871 [M + H]^+^ (calcd for C_22_H_17_O_9_, 425.0867, error = 0.94 ppm).

#### Actinomycetoquinone D (**4**)

Yellow powder; [α]_D_^25^ +52 (*c* 0.1, CH_3_OH:CHCl_3_ 1:1); UV (CH_3_OH:CHCl_3_ 1:1) λ_max_ (log ε) 245 (4.0), 268 (3.99), 422 (3.60) nm; IR (ATR) υ_max_ 3360.0, 2942.1, 1642.5, 1458.4, 1354.0, 1023.6 cm^–1^; ^1^H and ^13^C NMR (see Table 1); HRESIMS *m*/*z* 395.1128 [M + H]^+^ (calcd for C_22_H_19_O_7_, 395.1125, error = 0.75 ppm).

#### Actinomycetoquinone E (**5**)

Yellow powder; [α]_D_^25^ +37 (*c* 0.1, CH_3_OH:CHCl_3_ 1:1); UV (CH_3_OH:CHCl_3_ 1:1) λ_max_ (log ε) 243 (4.25), 268 (4.05), 422 (3.50) nm; IR (ATR) υ_max_ 3362.0, 2947.8, 1642.9, 1453.2, 1067.1, 1024.2 cm^–1; 1^H and ^13^C NMR (see Table 1); HRESIMS *m*/*z* 409.0923 [M + H]^+^ (calcd for C_22_H_17_O_8_, 409.0918, error = 1.22 ppm).

### X-ray Crystallographic Analysis of Actinomycetoquinone A

Crystallization from MeOH/H_2_O/acetic acid (9:1:0.001) using the vapor diffusion method yielded yellow crystals of actinomycetoquinone A. A yellow crystal with approximate dimensions of 0.11 × 0.03 × 0.03 mm^3^ was selected under oil under ambient conditions and attached to the tip of a MiTeGen MicroMount. The crystal was mounted in a stream of cold nitrogen at 100(1) K and centered in the X-ray beam by using a video camera. The crystal evaluation and data collection were performed on a Bruker D8 VENTURE PhotonIII four-circle diffractometer with Cu Kα (λ = 1.54178 Å) radiation with a detector to crystal distance of 4.0 cm. The initial cell constants were obtained from a 180° φ scan conducted at a 2θ = 50° angle with an exposure time of 3 s per frame. The reflections were successfully indexed by an automated indexing routine built into the APEX3 program. The final cell constants were calculated from a set of 9945 strong reflections from the actual data collection. The data were collected by using a full sphere data collection routine to survey reciprocal space to the extent of a full sphere to a resolution of 0.77 Å. A total of 43 505 data were harvested by collecting 46 sets of frames with 0.9° scans in ω and φ with an exposure time 4–20 s per frame. These highly redundant data sets were corrected for Lorentz and polarization effects. The absorption correction was based on fitting a function to the empirical transmission surface as sampled by multiple equivalent measurements.^22^ The diffraction data were consistent with the space groups *P*1̅ and *P*1. The *E*-statistics strongly suggested the noncentrosymmetric space group *P*1 that yielded chemically reasonable and computationally stable results of refinement. A successful solution by direct methods provided most non-hydrogen atoms from the *E*-map. The remaining non-hydrogen atoms were located in an alternating series of least-squares cycles and difference Fourier maps. All non-hydrogen atoms were refined with anisotropic displacement coefficients. All hydrogen atoms (except those bound to the O atoms) were included in the structure factor calculation at idealized positions and were allowed to ride on the neighboring atoms with relative isotropic displacement coefficients. The structure composition is [C_22_H_18_O_9_Cl]·0.5 H_2_O. There are two symmetry independent molecules in the unit cell. These have the same chemical composition and handedness but different conformations.

The absolute configurations for the two molecules were unequivocally [Flack *x* = 0.014(13)] established by anomalous dispersion effects: C2 and C2A, *S*; C3 and C3A, *R*.

The crystal chosen for the single-crystal X-ray diffraction experiment proved to be a three-component non-merohedral twin with contributions of ∼23 and ∼5.8% from the two minor components. Both minor domains are related to the first domain by a ∼5° rotation. The data were processed in several ways; the best results of refinement were obtained when the main domain was treated as a single crystal and the minor components were disregarded.

The final least-squares refinement of 623 parameters against 7848 data resulted in residuals *R* (based on *F* ^2^ for *I* ≥ 2σ) and *wR* (based on *F* ^2^ for all data) of 0.0322 and 0.0818, respectively. The final difference Fourier map was featureless. Crystal data for C_22_H_18_ClO_9.5_ (*M* = 469.81 g/mol): triclinic, space group *P*1 (no. 1), *a* = 6.9698(8) Å, *b* = 11.2605(16) Å, *c* = 12.5702(16) Å, α = 90.932(7)°, β = 99.055(6)°, γ = 99.003(10)°, *V* = 961.4(2) Å^3^, *Z* = 2, *T* = 100.0 K, μ­(Cu Kα) = 2.313 mm^–1^, *D*_calc_ = 1.623 g/cm^3^, 43 505 reflections measured (7.128° ≤ 2Θ ≤ 159.1°), 7848 unique (*R*_int_ = 0.0487, *R*_sigma_ = 0.0342), which were used in all calculations. The final *R*_1_ was 0.0322 (*I* > 2σ­(*I*)), and *wR*_2_ was 0.0818 (all data).

### Molecular Modeling and DFT Calculations

Using Spartan ‘18, a library of low energy conformers (MMFF) within 15 kJ/mol was generated. Each of these conformers then underwent geometry optimization and energy analysis using the Hartree–Fock method (HF/3-21G) and DFT calculations (M06-2X/aug-cc-pVDZ), respectively. Subsequently, the conformers were further optimized using DFT (B3LYP/6-31G*), and their NMR shifts were calculated via DFT (mPW1PW91/6-31+G**).^23^ The computed shifts were then compared to experimental values using the DP4+ probability method.^24^ For ECD calculations, Gaussian 16 was utilized, and the energies, oscillator strengths, and rotational strengths of the first 50 electronic excitations for each conformer were determined using the TDDFT methodology at the B3LYP/6-31G­(d) level in MeOH [Gaussian Keywords: td = (nstates = 50) b3lyp/6-31g­(d) scrf = (iefpcm, solvent = methanol) geom = connectivity] . To get the final spectra, the simulated spectra of the conformers were averaged according to the Boltzmann distribution theory.

### Antibacterial Assay

Compounds **1**–**5** were tested for antibacterial activity against methicillin-resistant *Staphylococcus aureus* (MRSA) (ATCC #33591), and *E. coli* (ATCC #25922). A dilution antimicrobial susceptibility test for aerobic bacteria was used to determine the MICs, and the MIC value was defined as the lowest concentration that inhibited visible growth of bacteria.^25^ Compounds **1**–**5** were dissolved in DMSO and serially diluted to 10 concentrations (0.125–64 μg/mL) in a 96-well plate. Vancomycin was used as a positive control with an MIC of 0.25 μg/mL against MRSA. Gentamicin was used as a positive control with an MIC of 4 μg/mL against *E. coli*. Compounds **1**–**5** and the positive controls were tested in triplicate. On each plate, there were six untreated media controls. The plates were incubated at 35 °C for 18 h.

## Supplementary Material



## Data Availability

NMR FID files for compounds **1**–**5** were submitted to the Natural Products Magnetic Resonance Database (NP-MRD) (NP0351519, NP0351520, NP0351521, NP0351522, and NP0351523), and high-resolution mass spectrometry (HRMS) data were deposited in the Global Natural Products Social Molecular Networking (GNPS) platform. All other data are available upon request.

## Notes

Comprehensive data sets for all compounds reported (PDF)
