# Supplementary material for: Actinomycetoquinones A–E, Anthraquinone-γ-Pyrones Discovered from Marine-Derived Actinomycetospora sp. Bacterium
Source: J Nat Prod. 2025 Sep 25;88(10):2325–32. doi: 10.1021/acs.jnatprod.5c00578 (PMC12503092; doi:10.1021/acs.jnatprod.5c00578)
Supplement: Supplementary file 1 [file np5c00578_si_001.pdf]

Actinomycetoquinones A−E, Anthraquinone-γ-Pyrones Discovered from Marine-Derived *Actinomycetospora* sp. Bacterium

Fan Zhang,[†] Christopher D. Roberts,[†] Tae Hyun Lee,[†] Doug R. Braun,[†] Zachary D Bennett[§], Shaurya Chanana,[†] Song Guo,[‡] Gene E. Ananiev,[‡] Ilia A. Guzei,[§] Scott R. Rajski,[†] Thomas C. Brunold[§], and Tim S. Bugni[*,†, ‡,ǁ]

[†]Pharmaceutical Sciences Division, University of Wisconsin–Madison, Madison, Wisconsin, 53706 United States.

[‡]Small Molecule Screening Facility, UW Carbone Cancer Center, Madison, Wisconsin, 53706 United States.

[§]Department of Chemistry, University of Wisconsin-Madison, Madison, Wisconsin, 53706, United States

[ǁ]Lachman Institute for Pharmaceutical Development, University of Wisconsin–Madison, Madison, 53706 Wisconsin, United States.

* To whom correspondence should be addressed. Tel.: 1-608-263-2519. E-mail: tim.bugni@wisc.edu.

Supporting Information Table of Contents

Contents **Page**

1) **Figure S1.** ^{1}H NMR spectrum of actinomycetoquinone A (**1**; 600 MHz, CD_3OD:$CDCl_3$ 1:1) ...4
2) **Figure S2.** ^{13}C NMR spectrum of actinomycetoquinone A (**1**; 150 MHz, CD_3OD:$CDCl_3$ 1:1) ...5
3) **Figure S3.** gCOSY spectrum of actinomycetoquinone A (**1**; 150 MHz, CD_3OD:$CDCl_3$ 1:1) 6
4) **Figure S4.** gHSQC spectrum of actinomycetoquinone A (**1**; 150 MHz, CD_3OD:$CDCl_3$ 1:1) 7
5) **Figure S5.** gHMBC spectrum of actinomycetoquinone A (**1**; 150 MHz, CD_3OD:$CDCl_3$ 1:1) 8
6) **Figure S6.** Positive ion HRESIMS of actinomycetoquinone A (**1**) 9
7) **Figure S7.** ECD spectrum of actinomycetoquinone A (**1**) 10
8) **Figure S8.** ^{1}H NMR spectrum of actinomycetoquinone B (**2**; 500 MHz, CD_3OD:$CDCl_3$ 1:1) 11
9) **Figure S9.** ^{13}C NMR spectrum of actinomycetoquinone B (**2**; 125 MHz, CD_3OD:$CDCl_3$ 1:1) 12
10) **Figure S10.** gCOSY spectrum of actinomycetoquinone B (**2**; 125 MHz, CD_3OD:$CDCl_3$ 1:1) ...13
11) **Figure S11.** gHSQC spectrum of actinomycetoquinone B (**2**; 125 MHz, CD_3OD:$CDCl_3$ 1:1) ...14
12) **Figure S12.** gHMBC spectrum of actinomycetoquinone B (**2**; 125 MHz, CD_3OD:$CDCl_3$ 1:1) ..15
13) **Figure S13.** Positive ion HRESIMS of actinomycetoquinone B (**2**) 16
14) **Figure S14.** Positive ion ESI-MS/MS spectrum of actinomycetoquinone B (**2**) 17
15) **Figure S15.** Calculated and Experimental ECD spectrum of actinomycetoquinone B (**2**) . ..18
16) **Figure S16.** ^{1}H NMR spectrum of actinomycetoquinone C (**3**; 600 MHz, DMSO-d_6) 19
17) **Figure S17.** ^{13}C NMR spectrum of actinomycetoquinone C (**3**; 150 MHz, DMSO-d_6)................20
18) **Figure S18.** gCOSY spectrum of actinomycetoquinone C (**3**; 150 MHz, DMSO-d_6) 21
19) **Figure S19.** gHSQC spectrum of actinomycetoquinone C (**3**; 150 MHz, DMSO-d_6) 22
20) **Figure S20.** gHMBC spectrum of actinomycetoquinone C (**3**; 150 MHz, DMSO-d_6) 23
21) **Figure S21.** Positive ion HRESIMS of actinomycetoquinone C (**3**) 24
22) **Figure S22.** Calculated and Experimental ECD spectrum of actinomycetoquinone C (**3**) 25
23) **Figure S23.** ^{1}H NMR spectrum of actinomycetoquinone D (**4**; 600 MHz, CD_3OD:$CDCl_3$ 1:1) ..26
24) **Figure S24.** ^{13}C NMR spectrum of actinomycetoquinone D (**4**; 150 MHz, CD_3OD:$CDCl_3$ 1:1) ..27
25) **Figure S25.** gCOSY spectrum of actinomycetoquinone D (**4**; 150 MHz, CD_3OD:$CDCl_3$ 1:1) 28
26) **Figure S26.** gHSQC spectrum of actinomycetoquinone D (**4**; 150 MHz, CD_3OD:$CDCl_3$ 1:1) 29
27) **Figure S27.** gHMBC spectrum of actinomycetoquinone D (**4**; 150 MHz, CD_3OD:$CDCl_3$ 1:1) ...30
28) **Figure S28.** Positive ion HRESIMS of actinomycetoquinone D (**4**) 31
28) **Figure S29.** Calculated and Experimental ECD spectrum of actinomycetoquinone D (**4**)…..........32
30) **Figure S30.** ^{1}H NMR spectrum of actinomycetoquinone E (**5**; 600 MHz, CD_3OD:$CDCl_3$ 1:1) ..33
31) **Figure S31.** ^{13}C NMR spectrum of actinomycetoquinone E (**5**; 150 MHz, CD_3OD:$CDCl_3$ 1:1) ..34
32) **Figure S32.** gCOSY spectrum of actinomycetoquinone E (**5**; 150 MHz, CD_3OD:$CDCl_3$ 1:1) 35
33) **Figure S33.** gHSQC spectrum of actinomycetoquinone E (**5**; 150 MHz, CD_3OD:$CDCl_3$ 1:1) 36
34) **Figure S34.** gHMBC spectrum of actinomycetoquinone E (**5**; 150 MHz, CD_3OD:$CDCl_3$ 1:1) ...37
35) **Figure S35.** Positive ion HRESIMS of actinomycetoquinone E (**5**) 38
36) **Figure S36.** Calculated and Experimental ECD spectrum of actinomycetoquinone E (**5**) 39
37) **Figure S37.** A molecular drawing of actinomycetoquinone A (**1**) shown with 50% probability ellipsoids and selected atom labels. All H atoms bound to C atoms (except those bound to atoms C2 and C2A) are omitted 40

38) **Figure S38.** A molecular overlay of the two symmetry-independent molecules in actinomycetoquinone A (**1**) shown with 50% probability ellipsoids and selected atom labels 41
39) **Table S1**. Crystal data and structure refinement for actinomycetoquinone A (**1**) 42
40) **Table S2.** Fractional atomic coordinates (×10^4) and equivalent isotropic displacement parameters (Å^2×10^3) for actinomycetoquinone A (**1**). U_{eq} is defined as 1/3 of of the trace of the orthogonalised U_{IJ} 43
41) **Table S3.** Anisotropic displacement parameters (Å^2×10^3) for actinomycetoquinone A (**1**). The anisotropic displacement factor exponent takes the form: $-2\pi^2[h^2a^{*2}U_{11}+2hka^*b^*U_{12}+\ldots]$ 45
42) **Table S4.** Bond lengths for actinomycetoquinone A (**1**) 47
43) **Table S5.** Bond angles for actinomycetoquinone A (**1**) 48
44) **Table S6.** Hydrogen bonds for actinomycetoquinone A (**1**) 50
45) **Table S7.** Torsion angles for actinomycetoquinone A (**1**) 51
46) **Table S8.** Hydrogen atom coordinates (Å×10^4) and isotropic displacement parameters (Å^2×10^3) for actinomycetoquinone A (**1**) 53
47) **Table S9.** ^{1}H and ^{13}C NMR data for **2** (500 MHz for ^{1}H, 125 MHz for ^{13}C, CD_3OD:$CDCl_3$ 1:1)..54
48) **Table S10.** ^{1}H and ^{13}C NMR data for **3** (600 MHz for ^{1}H, 150 MHz for ^{13}C, DMSO-*d*$_6$) 55
49) **Table S11.** ^{1}H and ^{13}C NMR data for **4** (600 MHz for ^{1}H, 150 MHz for ^{13}C, CD_3OD:$CDCl_3$ 1:1).. 56
50) **Table S12.** ^{1}H and ^{13}C NMR data for **5** (600 MHz for ^{1}H, 150MHz for ^{13}C, CD_3OD:$CDCl_3$ 1:1)... 57

Figure S1. ^{1}H NMR spectrum of actinomycetoquinone A (**1**; 600 MHz, $CD_3OD:CDCl_3$ 1:1)

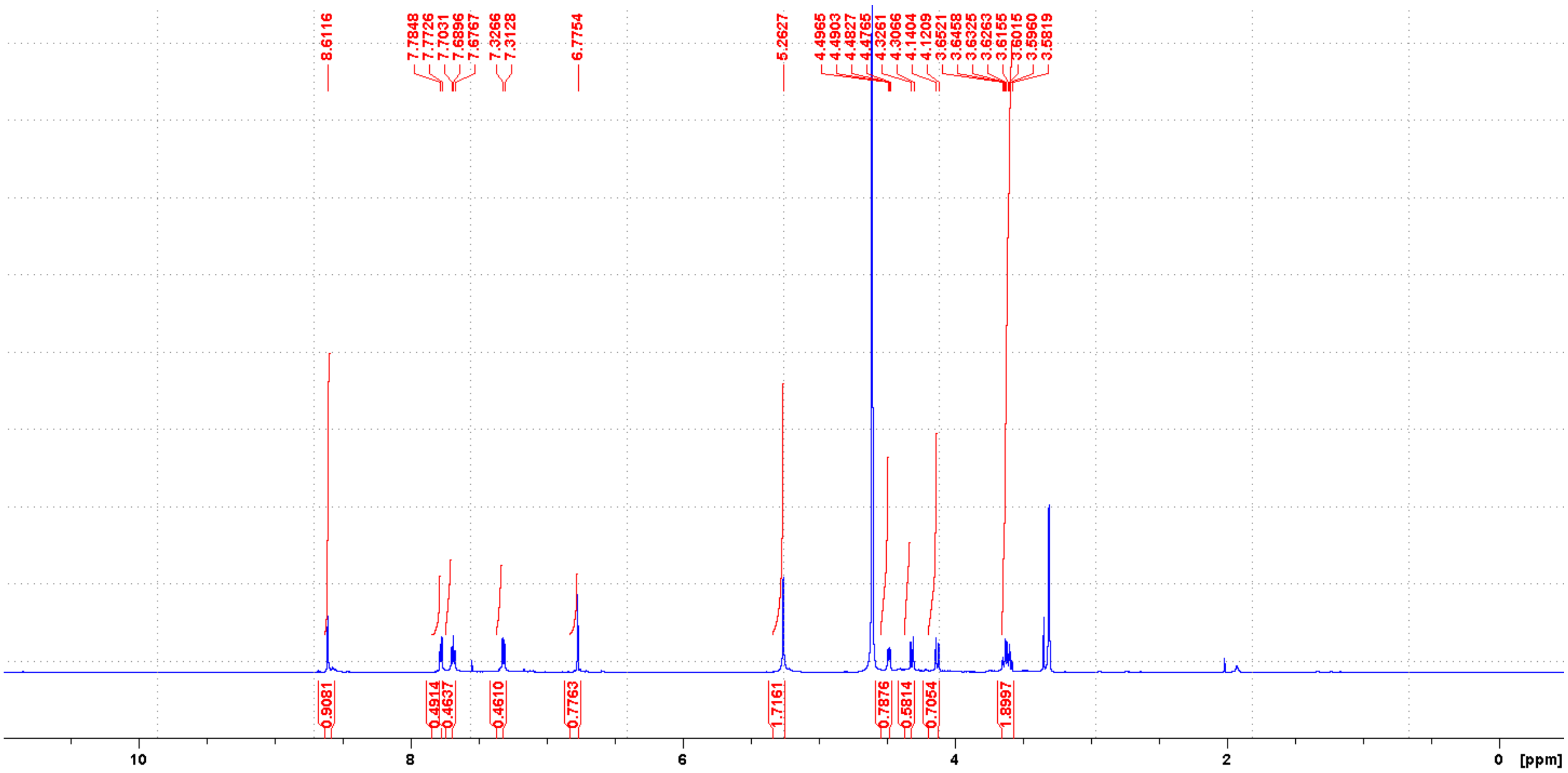

Figure S2. ^{13}C NMR spectrum of actinomycetoquinone A (**1**; 150 MHz, CD_3OD:$CDCl_3$ 1:1)

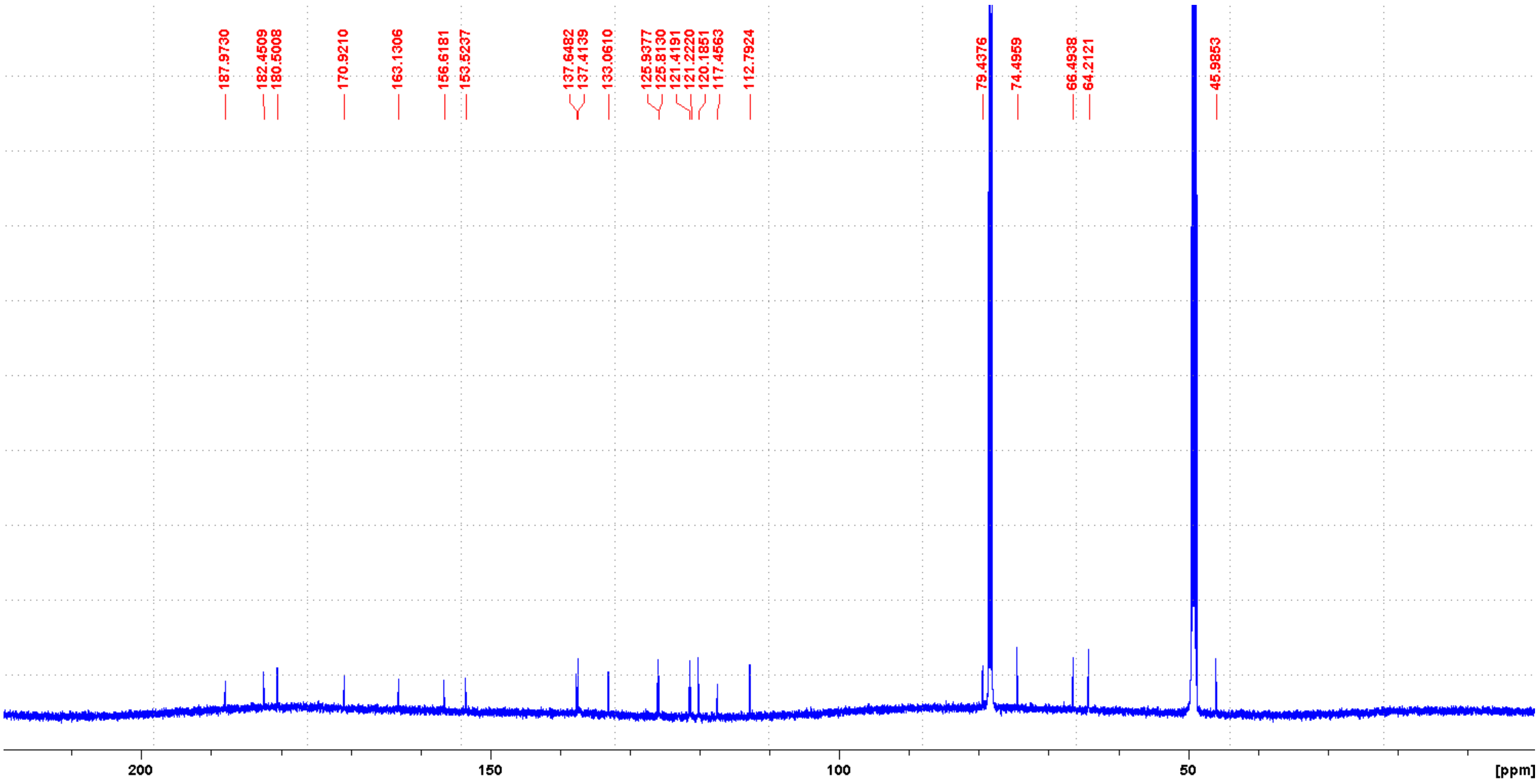

Figure S3. gCOSY spectrum of actinomycetoquinone A (**1**; 150 MHz, $CD_3OD:CDCl_3$ 1:1)

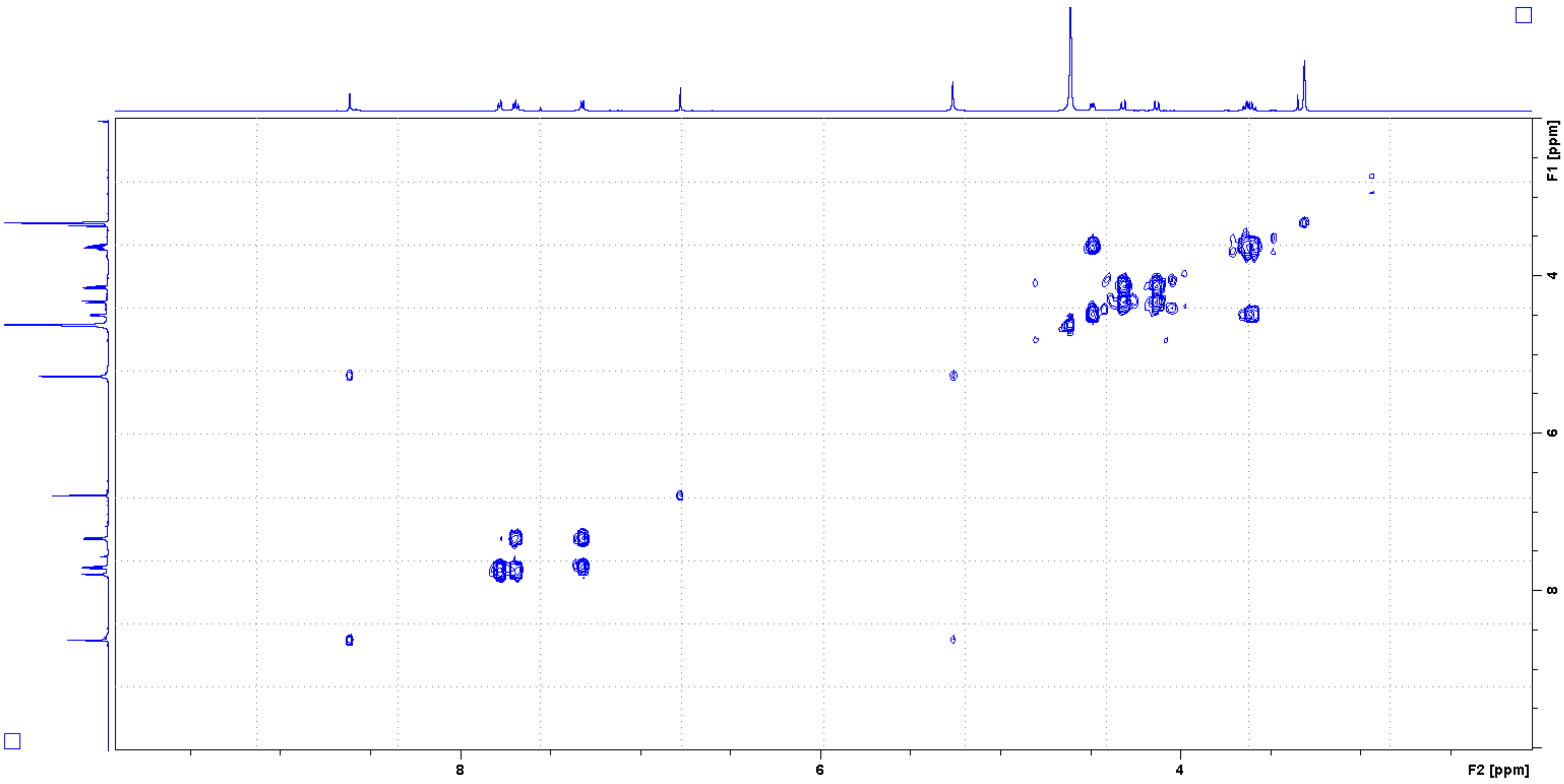

Figure S4. gHSQC spectrum of actinomycetoquinone A (**1**; 150 MHz, $CD_3OD:CDCl_3$ 1:1)

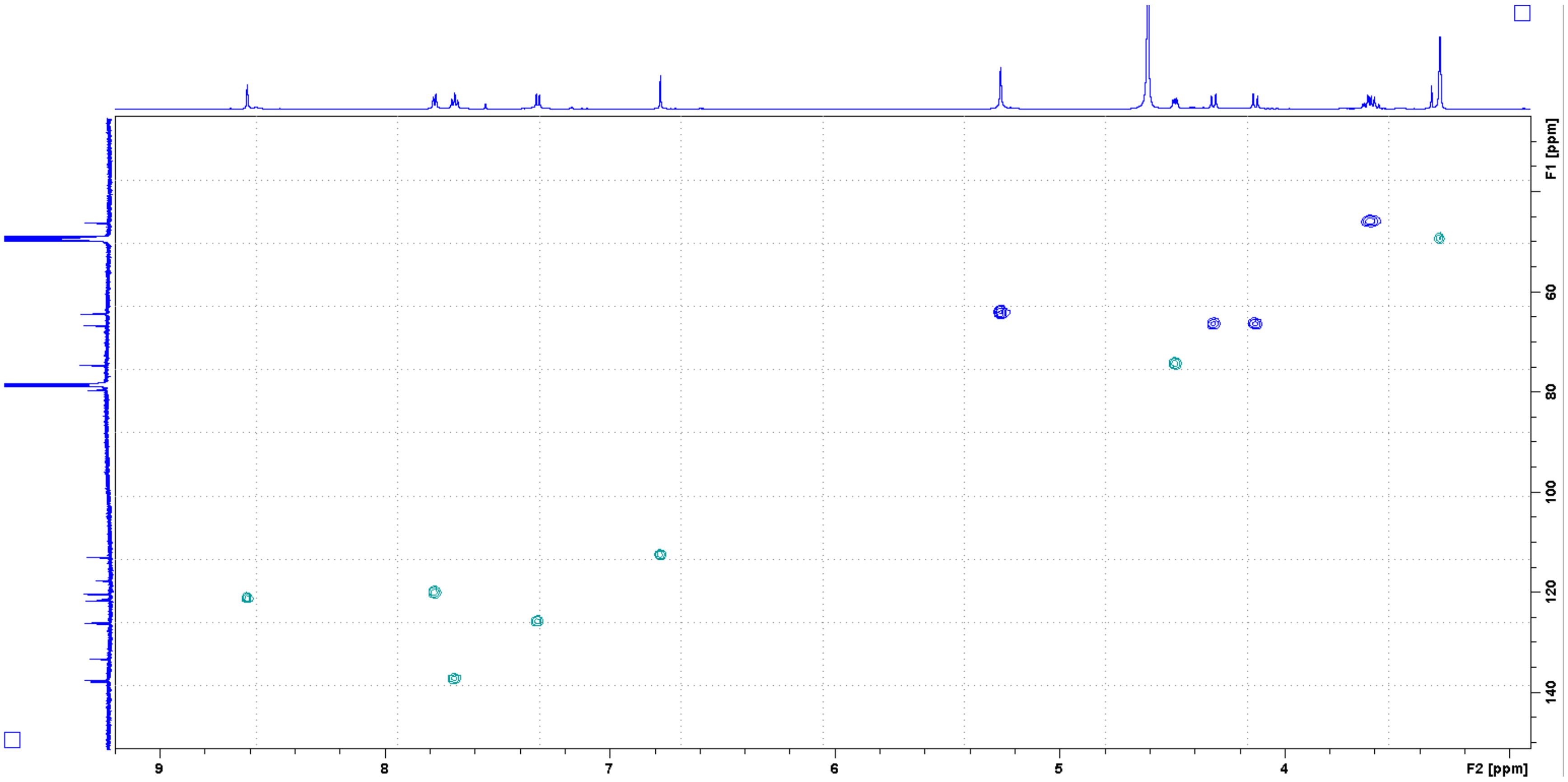

Figure S5. gHMBC spectrum of actinomycetoquinone A (**1**; 150 MHz, $CD_3OD:CDCl_3$ 1:1)

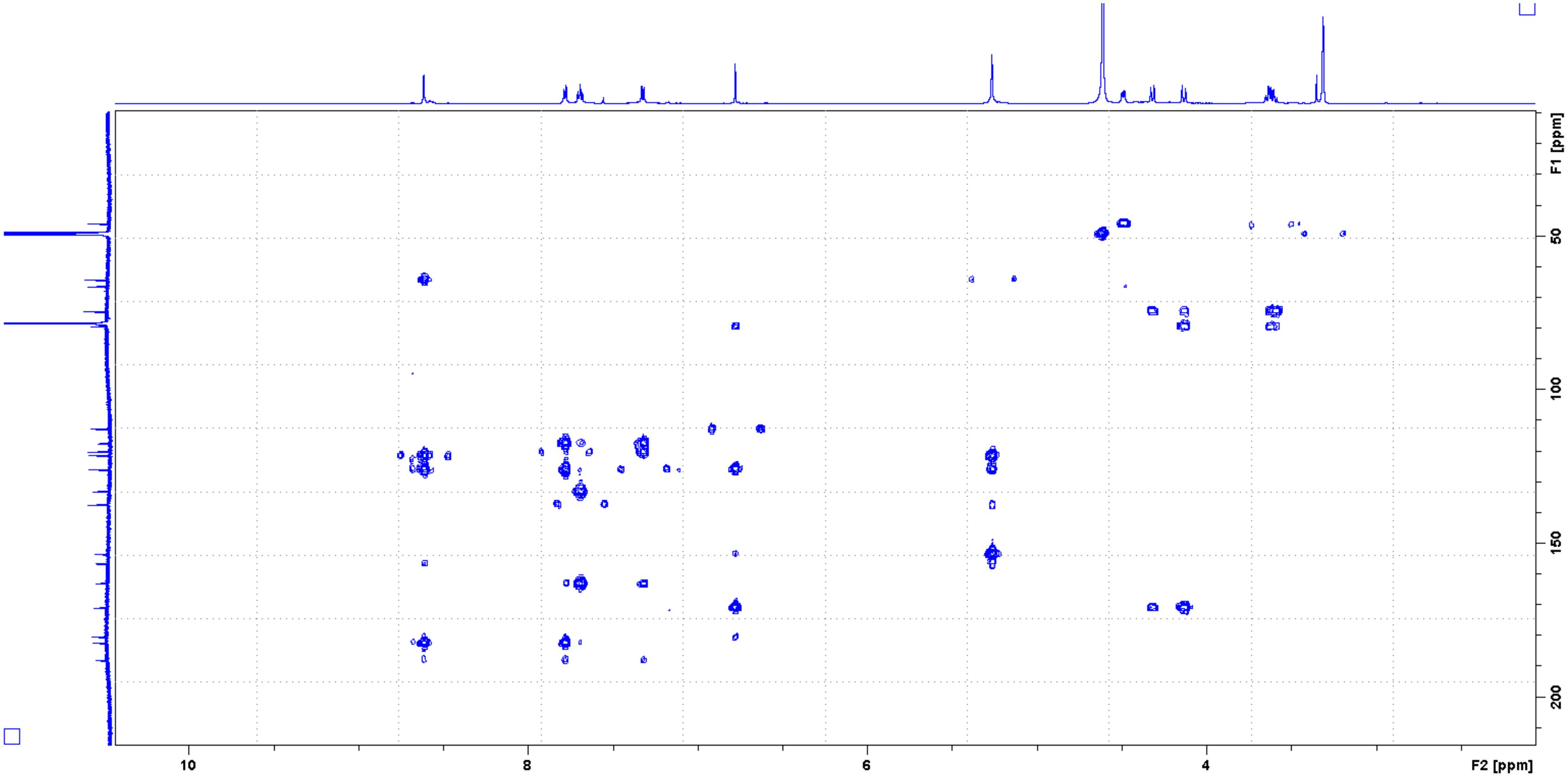

Figure S6. Positive ion HRESIMS of actinomycetoquinone A (**1**)

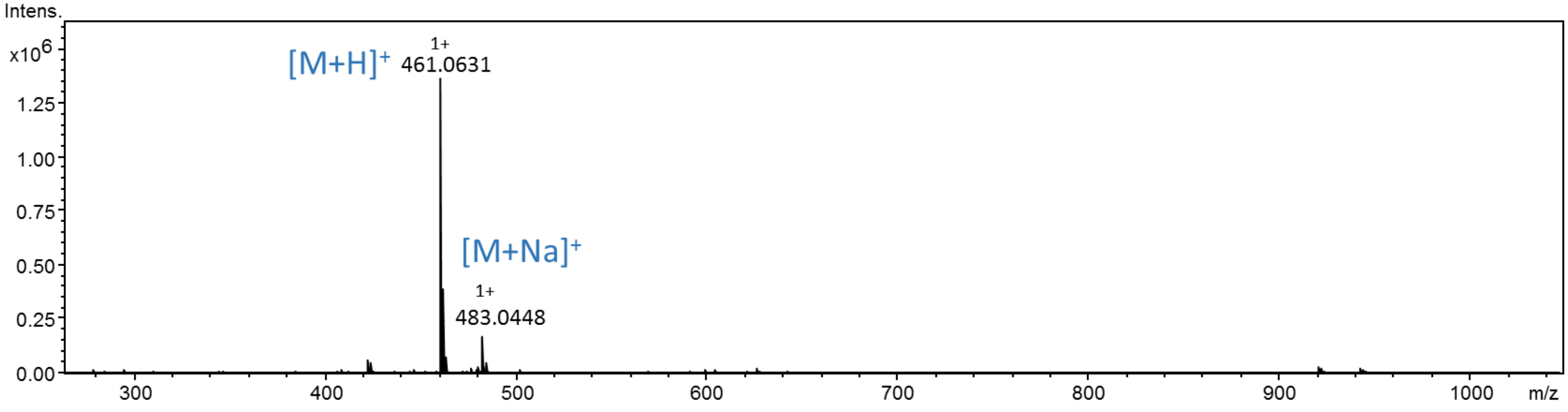

Figure S7. ECD spectrum of actinomycetoquinone A (**1**)

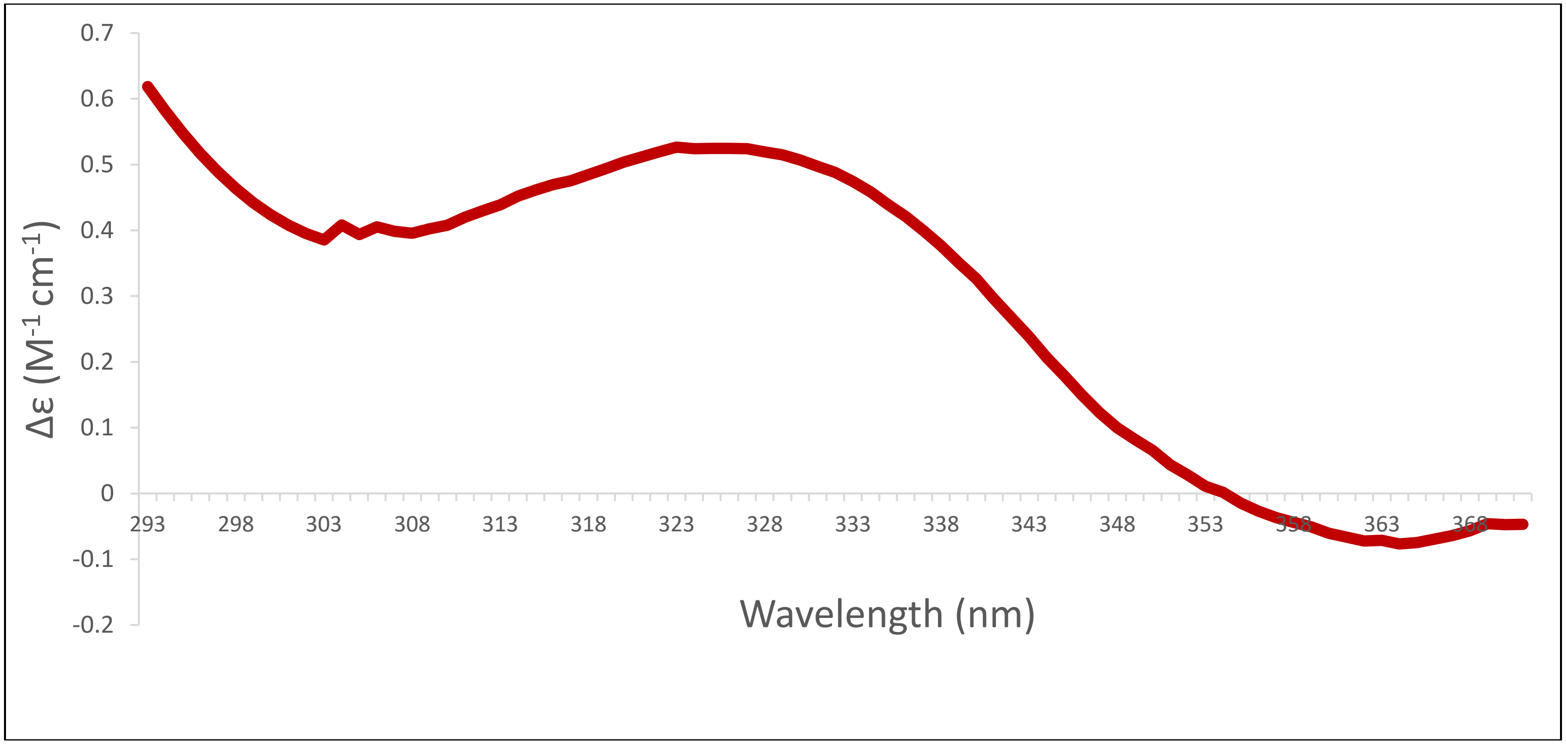

Figure S8. ^{1}H NMR spectrum of actinomycetoquinone B (**2**; 500 MHz, CD_3OD:$CDCl_3$ 1:1)

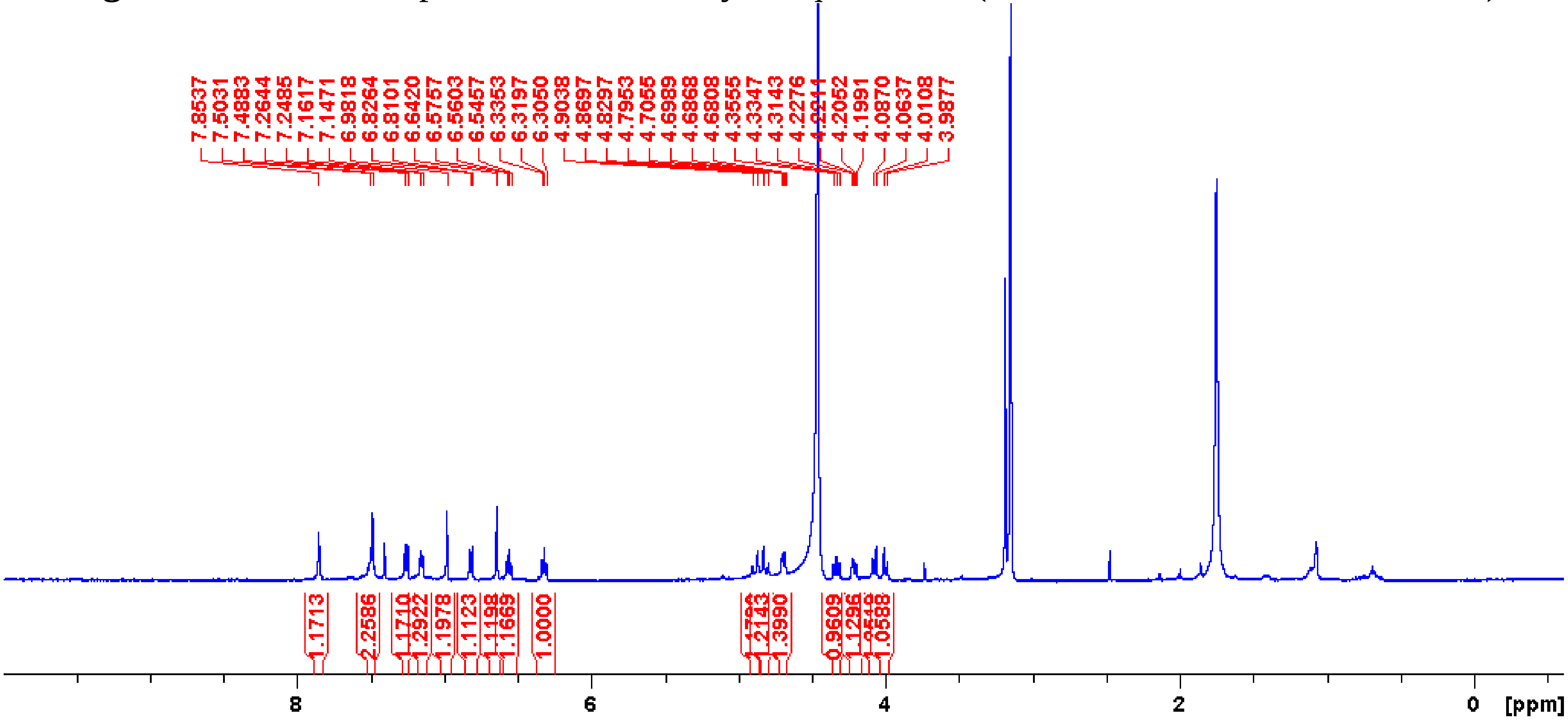

Figure S9. ^{13}C NMR spectrum of actinomycetoquinone B (**2**; 125 MHz, CD_3OD:$CDCl_3$ 1:1)

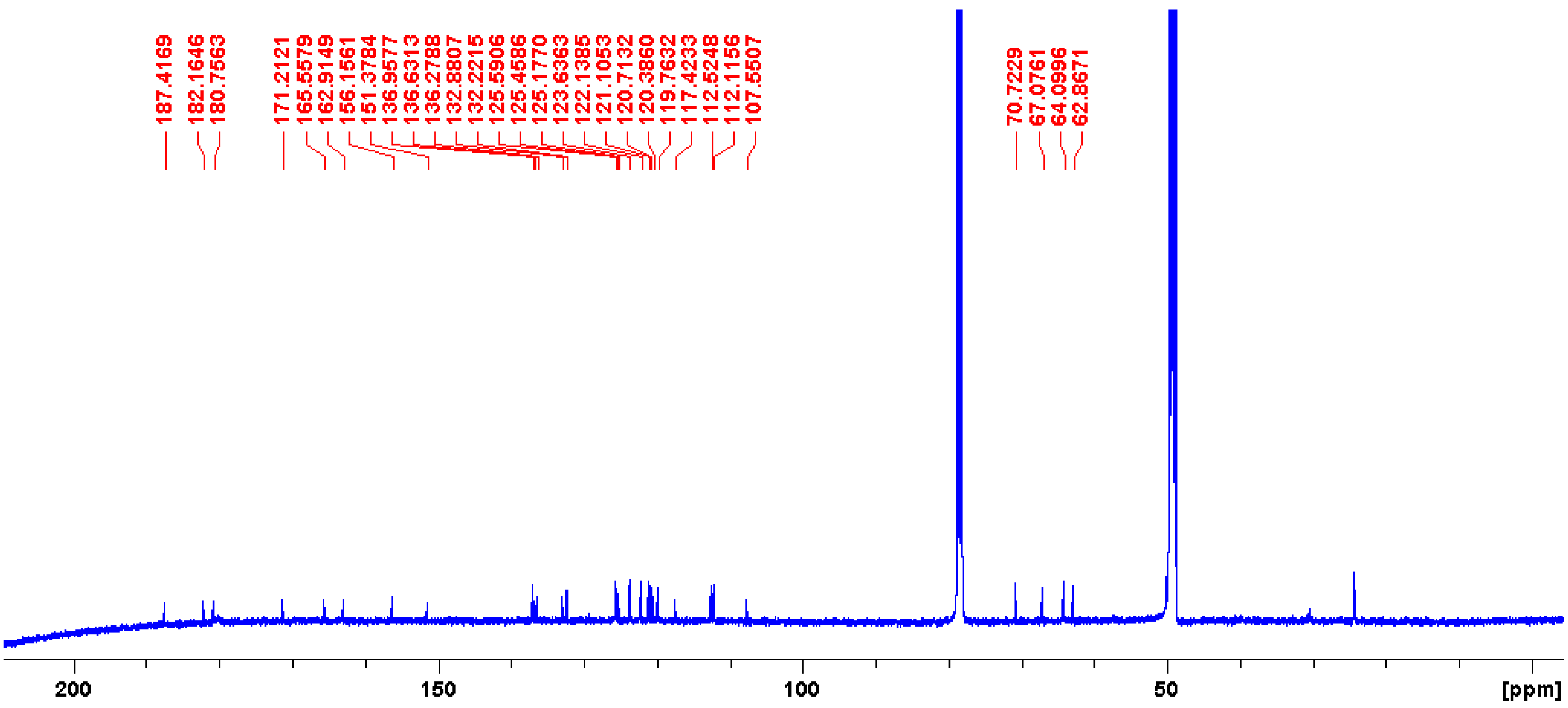

Figure S10. gCOSY spectrum of actinomycetoquinone B (**2**; 125 MHz, $CD_3OD:CDCl_3$ 1:1)

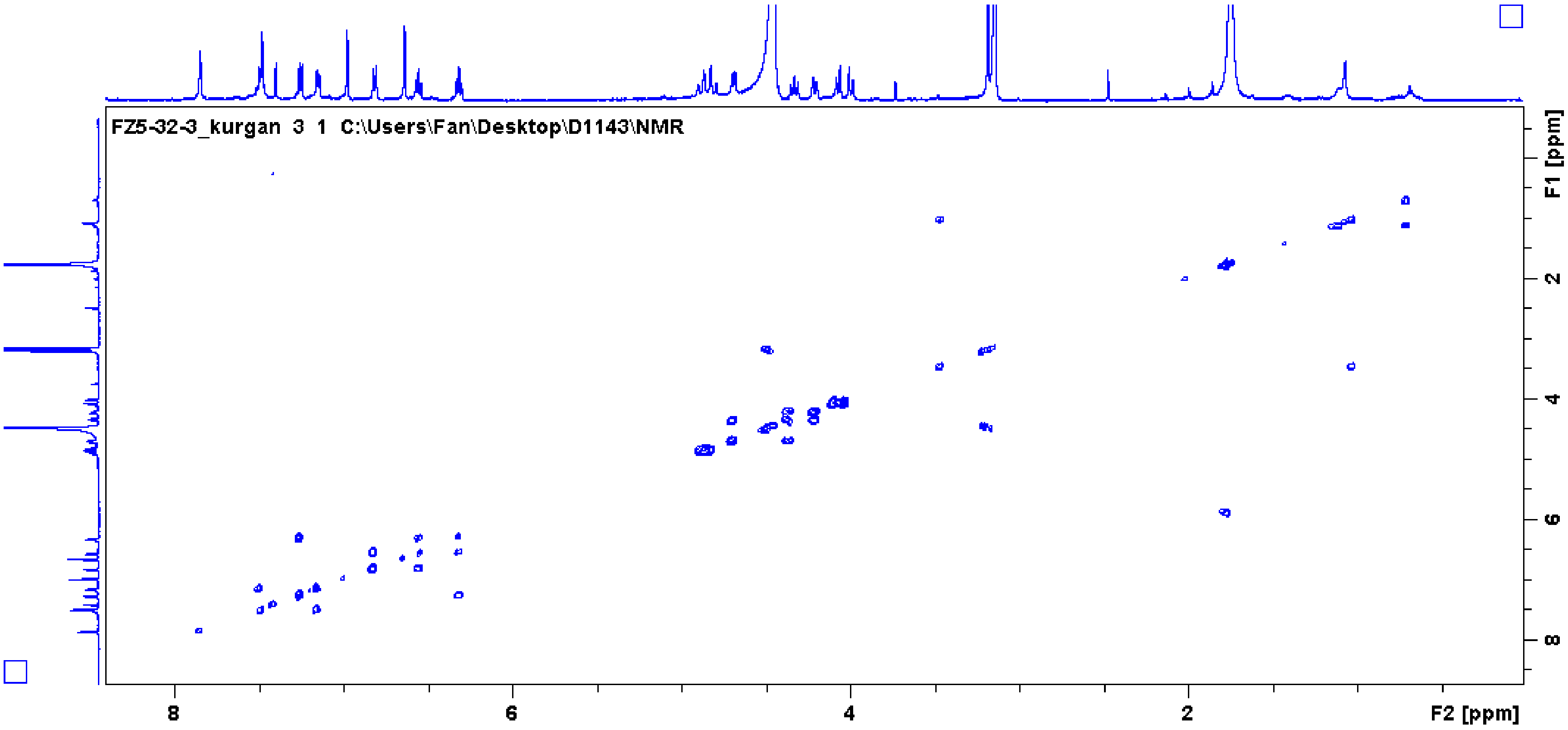

Figure S11. gHSQC spectrum of actinomycetoquinone B (**2**; 125 MHz, $CD_3OD:CDCl_3$ 1:1)

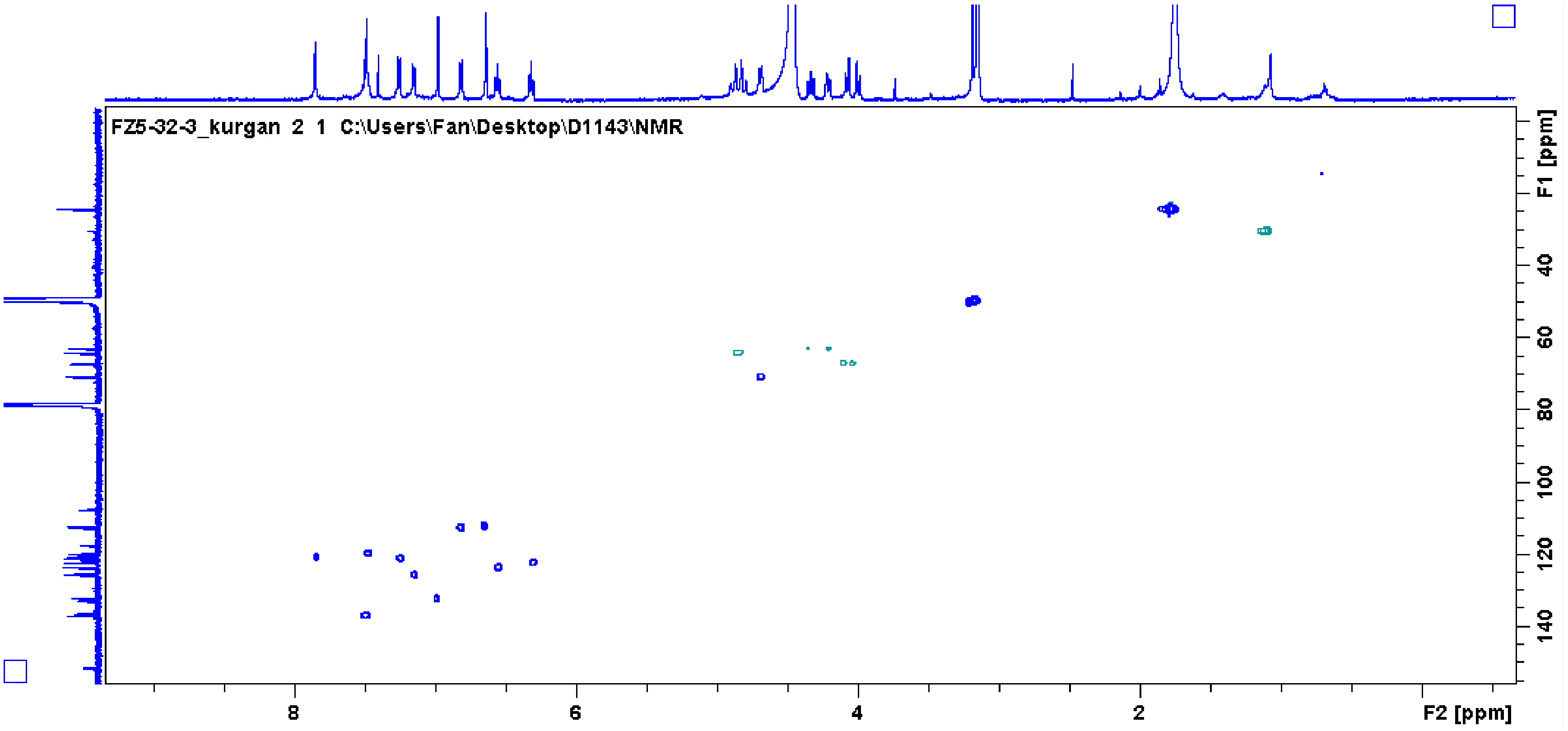

Figure S12. gHMBC spectrum of actinomycetoquinone B (**2**; 125 MHz, $CD_3OD:CDCl_3$ 1:1)

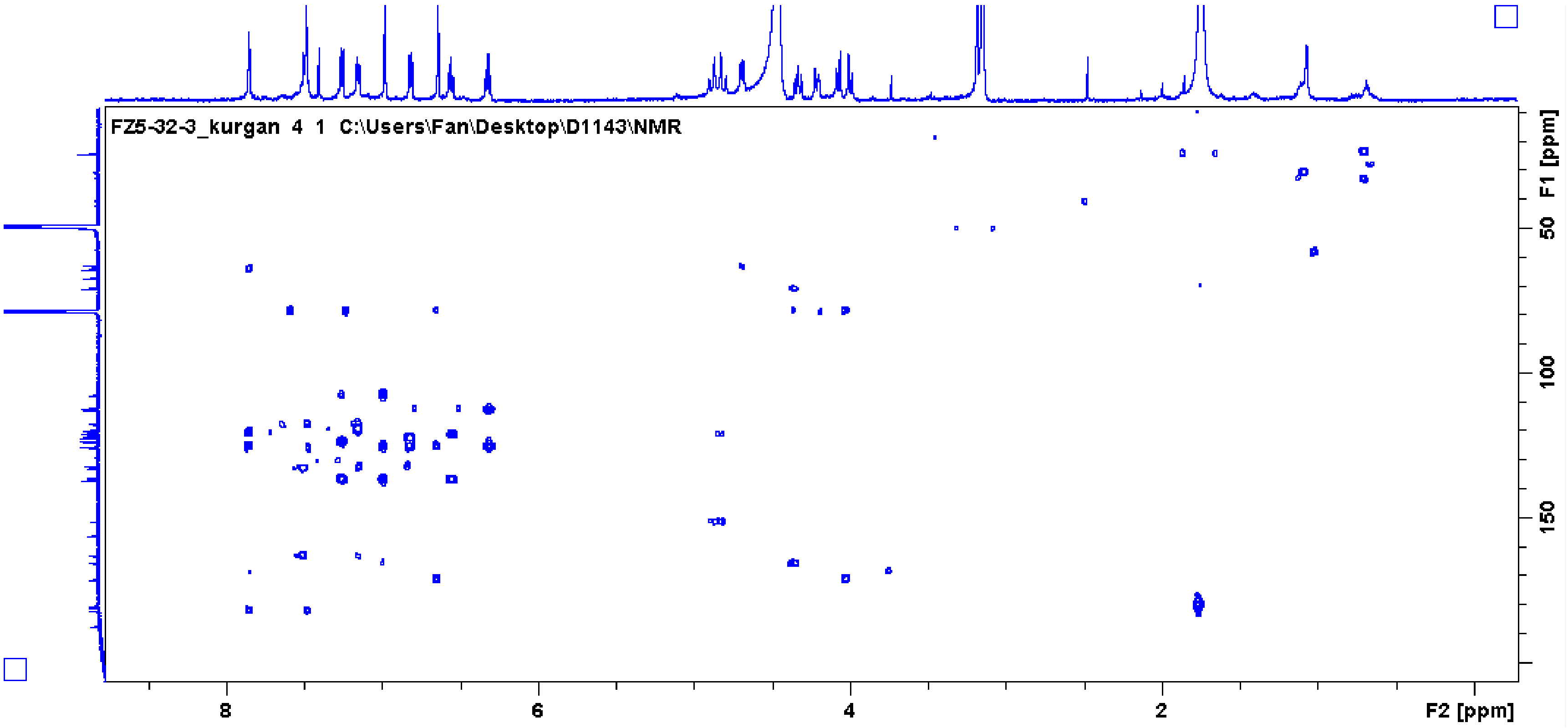

Figure S13. Positive ion HRESIMS of actinomycetoquinone B (**2**)

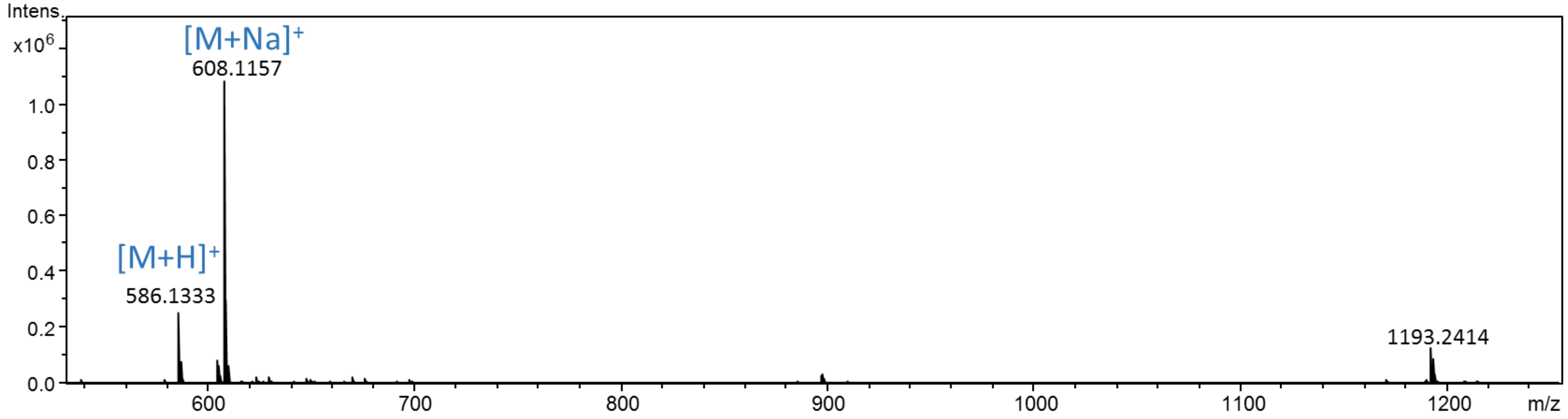

Figure S14. Positive ion ESI-MS/MS spectrum of actinomycetoquinone B (**2**)

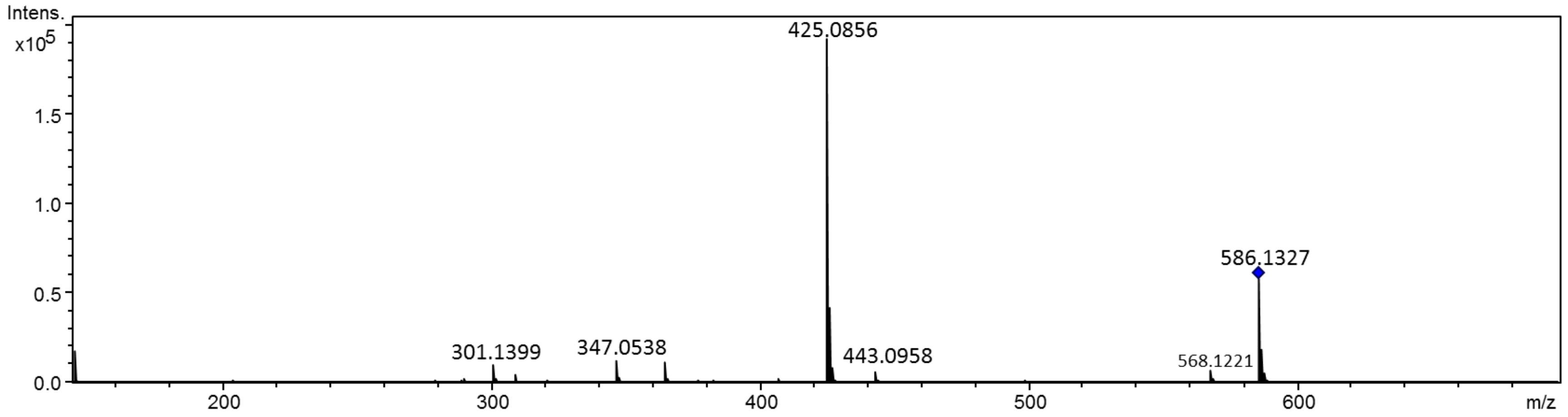

Figure S15. ECD spectrum of actinomycetoquinone B (**2**)

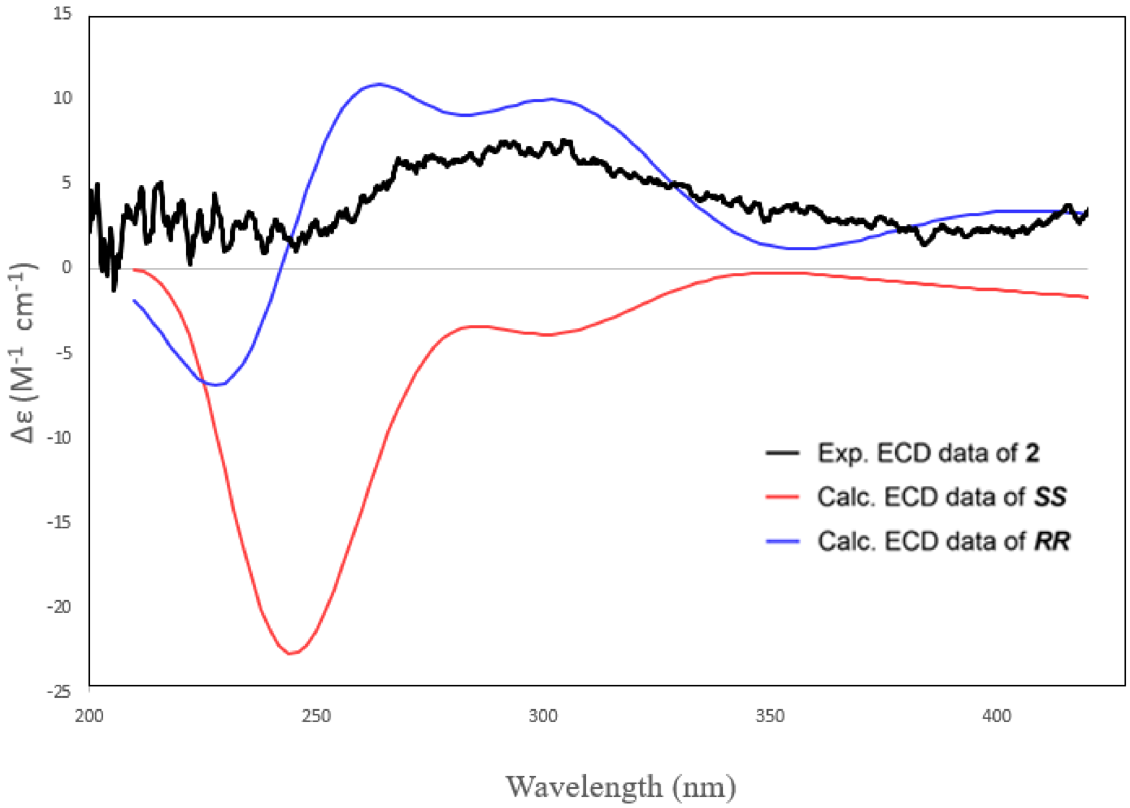

Figure S16. ^{1}H NMR spectrum of actinomycetoquinone C (**3**; 600 MHz, DMSO-d_6)

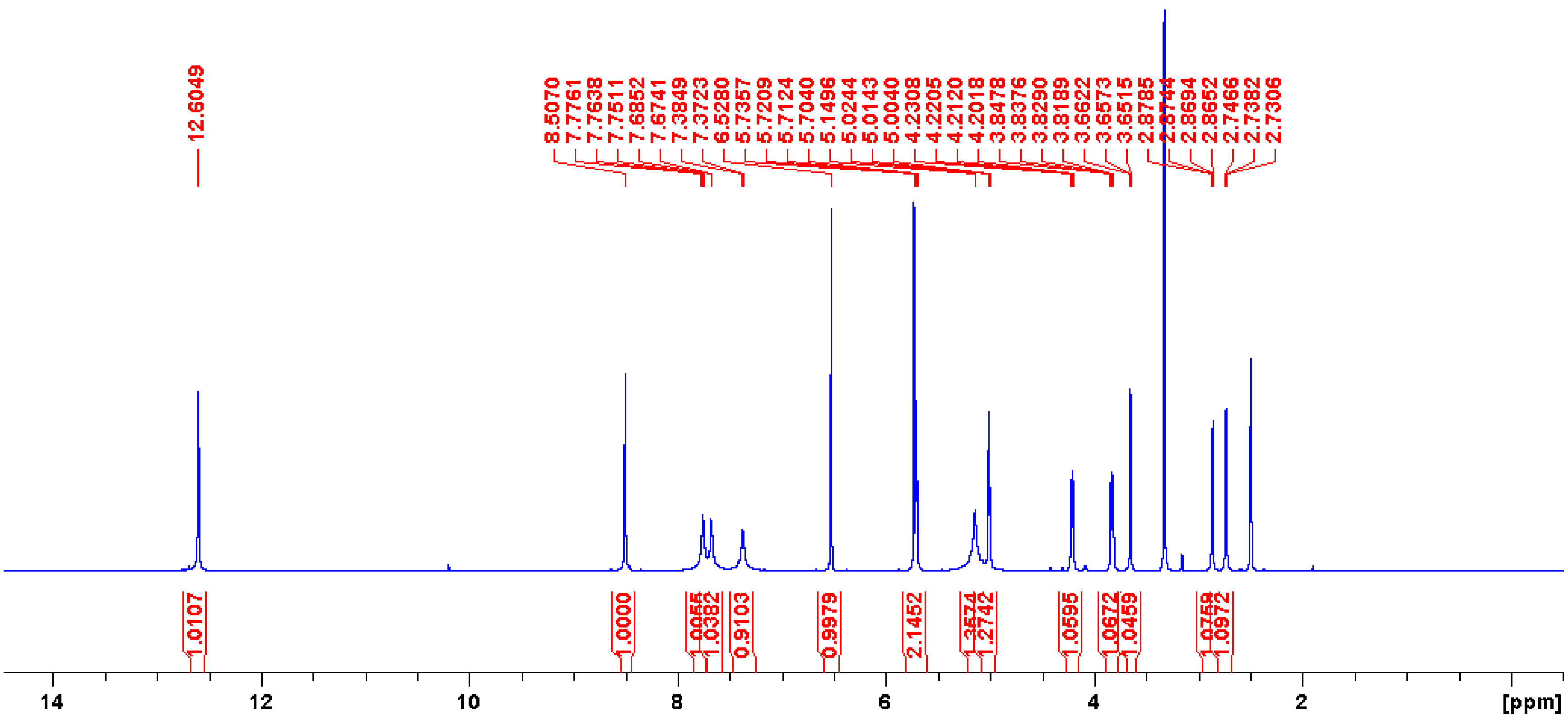

Figure S17. ^{13}C NMR spectrum of actinomycetoquinone C (**3**; 150 MHz, DMSO-d_6)

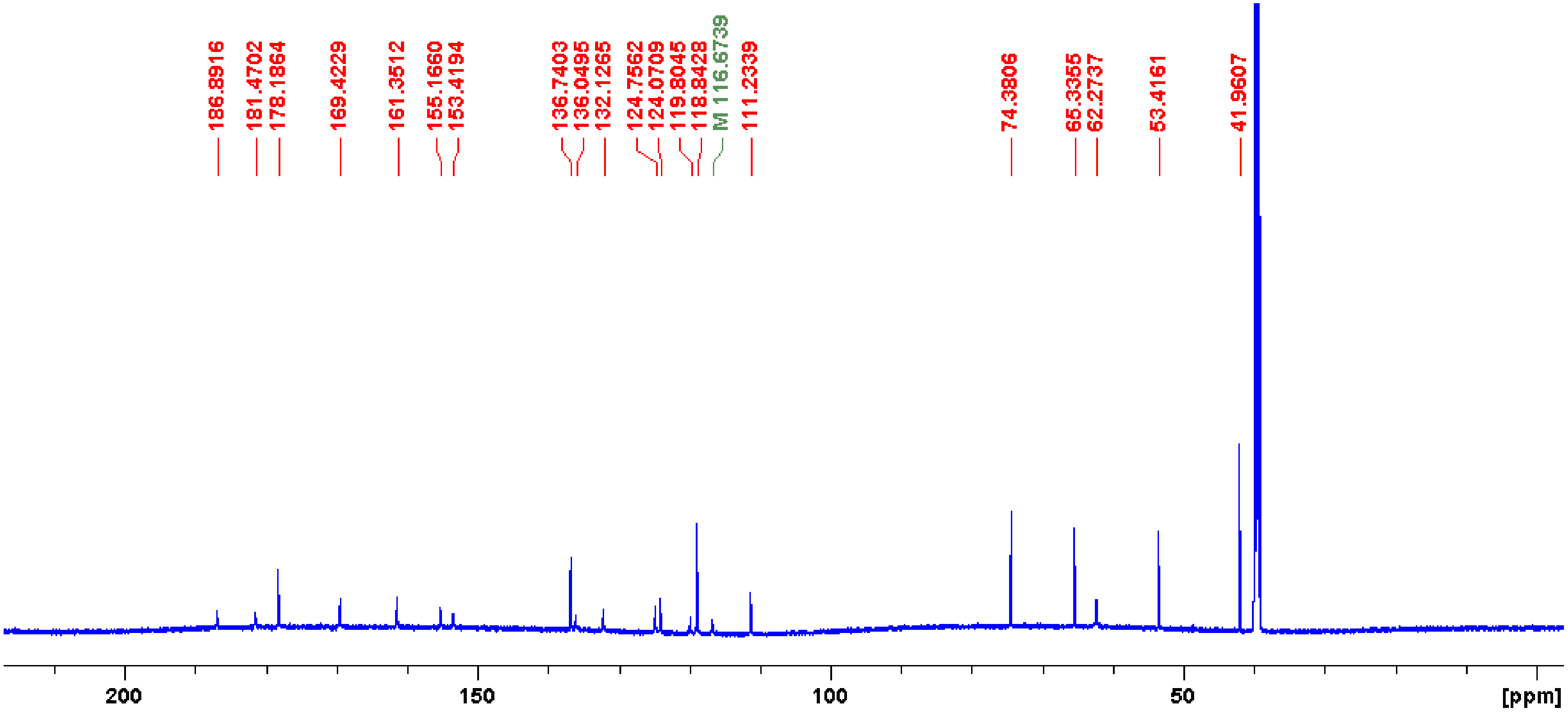

Figure S18. gCOSY spectrum of actinomycetoquinone C (**3**; 150 MHz, DMSO-d_6)

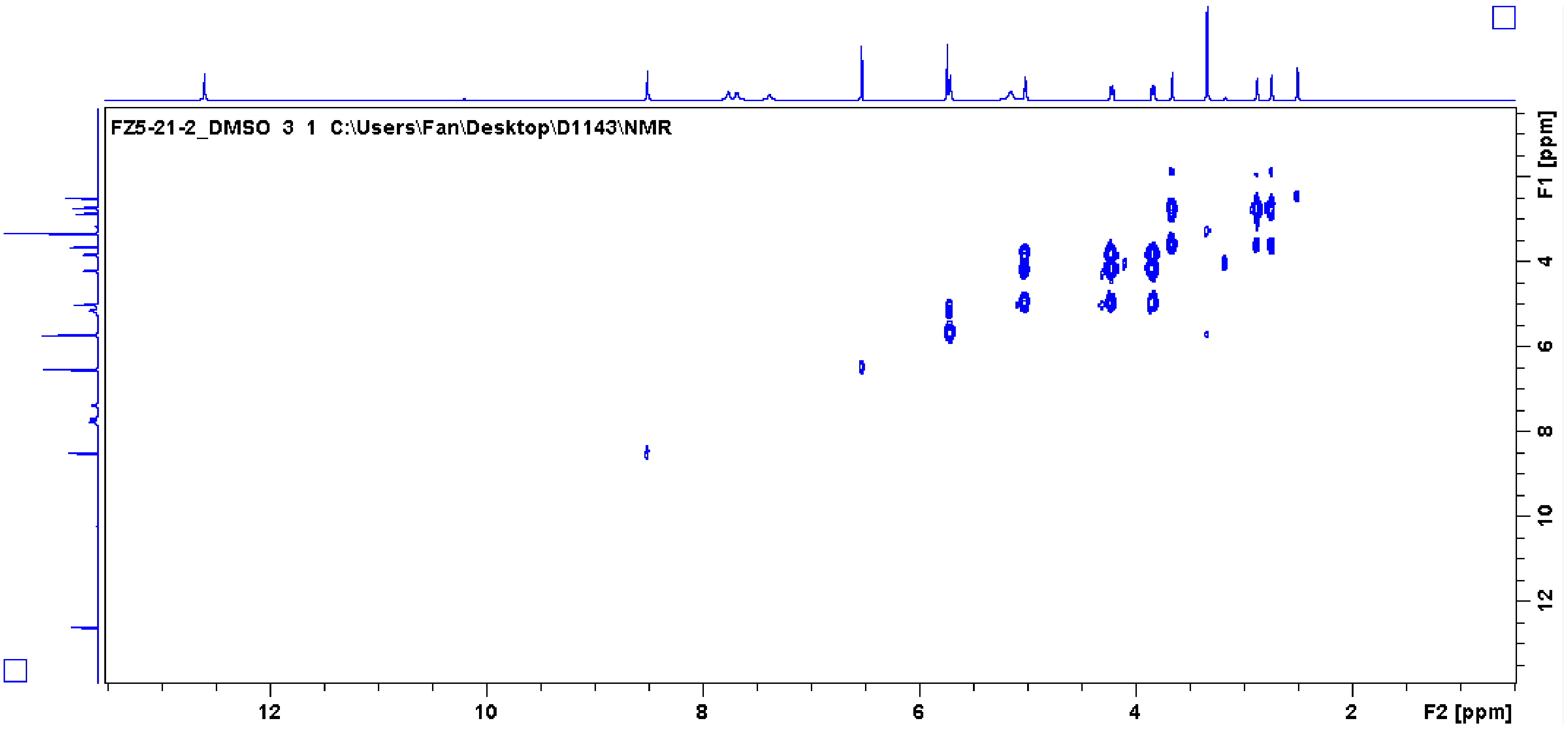

Figure S19. gHSQC spectrum of actinomycetoquinone C (**3**; 150 MHz, DMSO-d_6)

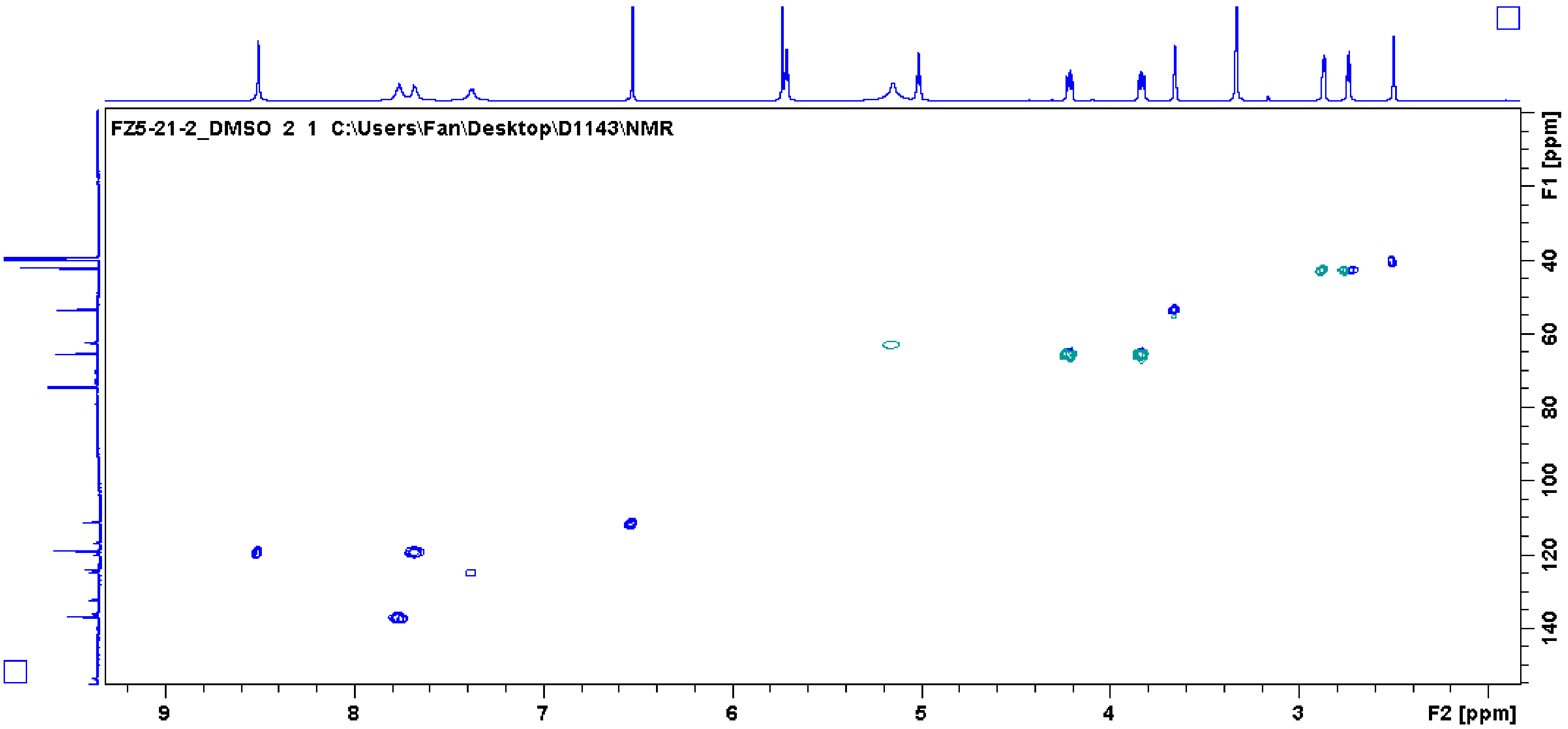

Figure S20. gHMBC spectrum of actinomycetoquinone C (**3**; 150 MHz, DMSO-d_6)

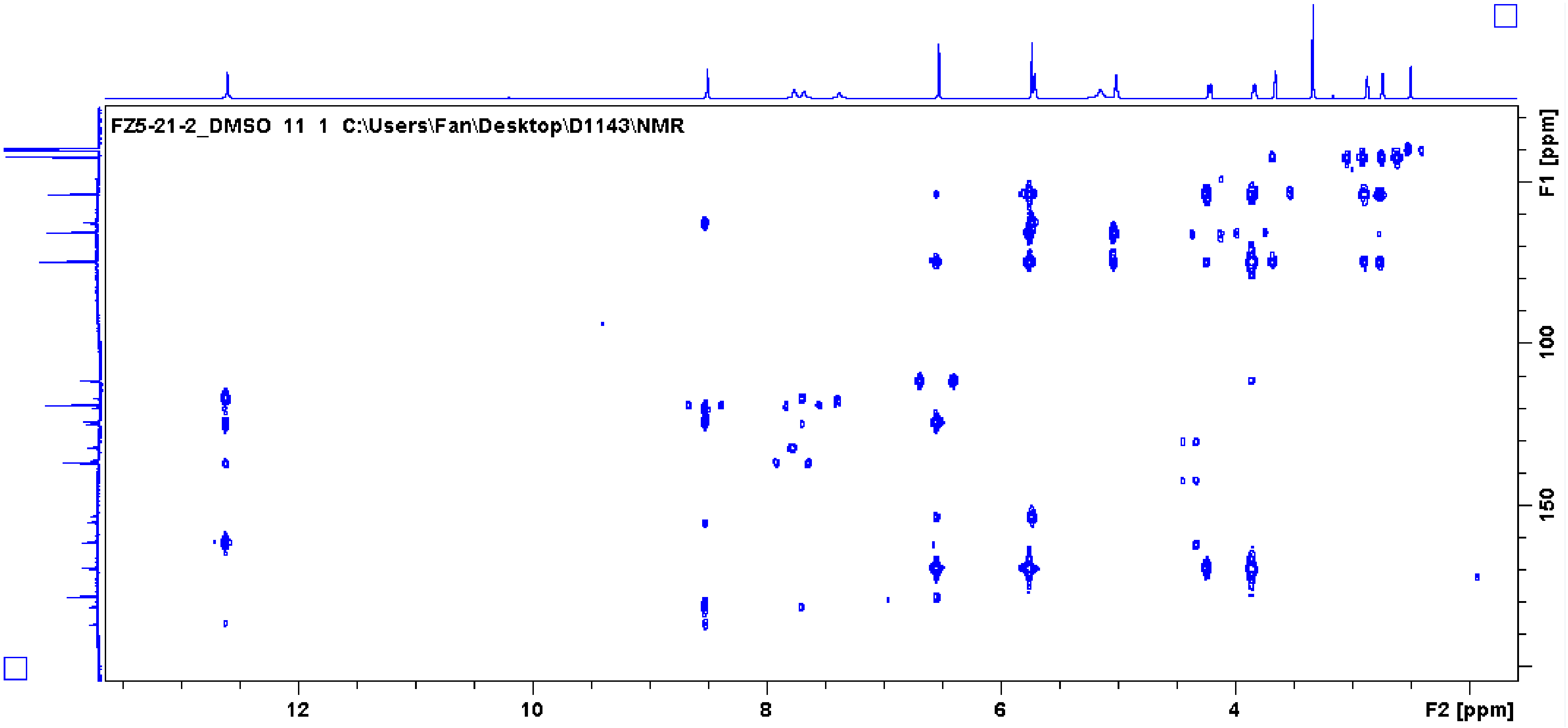

Figure S21. Positive ion HRESIMS of actinomycetoquinone C (**3**)

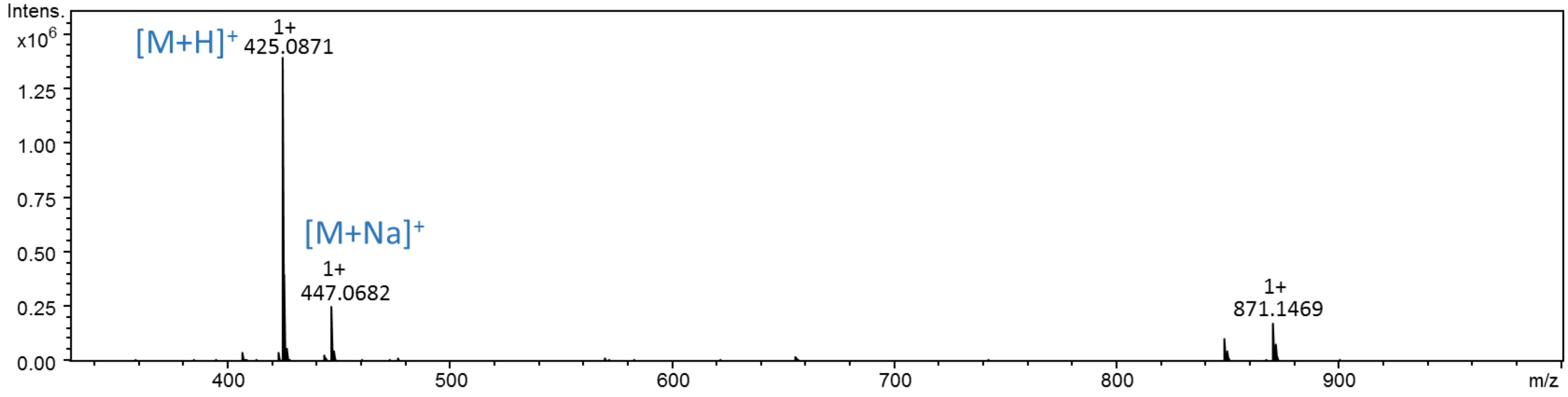

Figure S22. Calculated and Experimental ECD spectrum of actinomycetoquinone C (**3**)

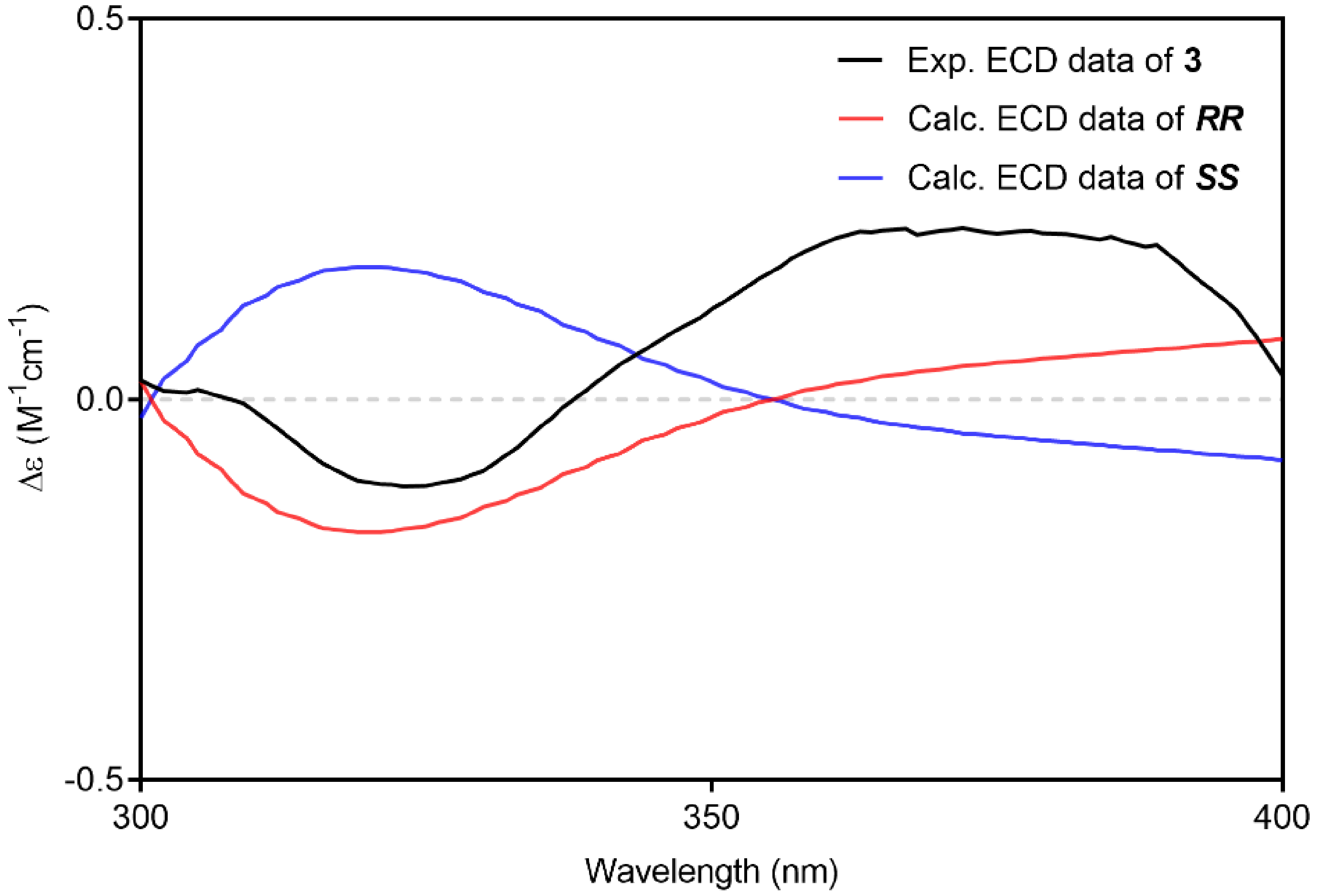

Figure S23. ^{1}H NMR spectrum of actinomycetoquinone D (**4**; 600 MHz, CD_3OD:$CDCl_3$ 1:1)

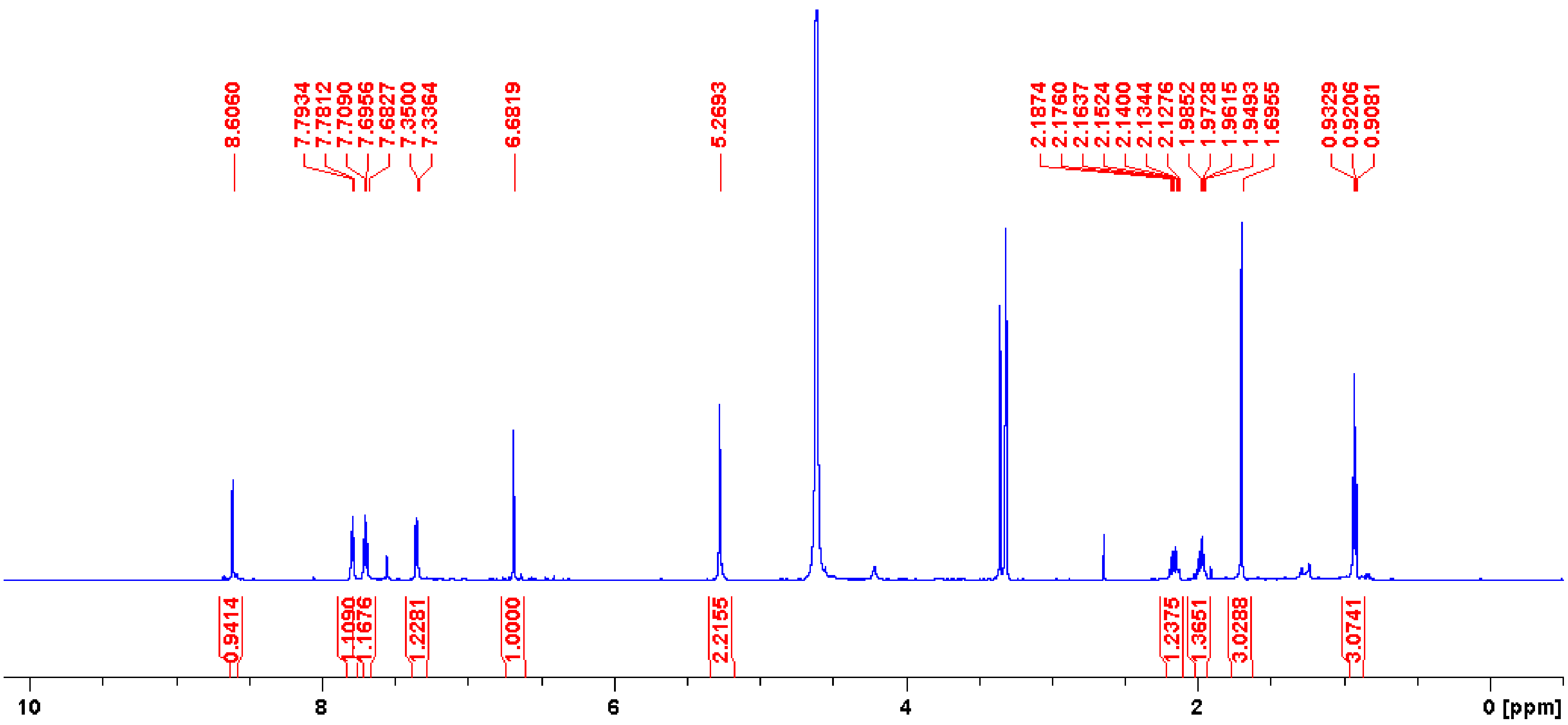

Figure S24. ^{13}C NMR spectrum of actinomycetoquinone D (**4**; 150 MHz, $CD_3OD:CDCl_3$ 1:1)

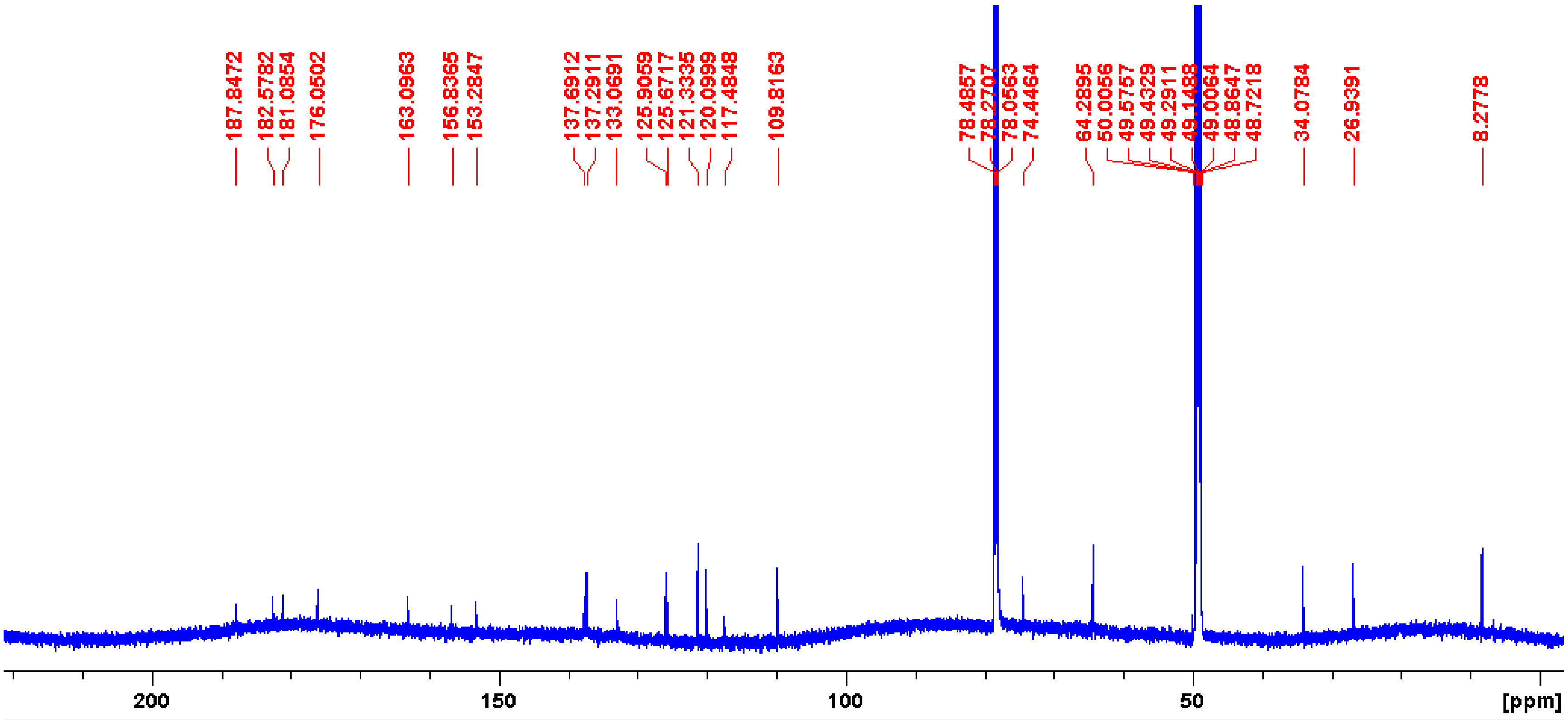

Figure S25. gCOSY spectrum of actinomycetoquinone D (**4**; 150 MHz, CD_3OD:$CDCl_3$ 1:1)

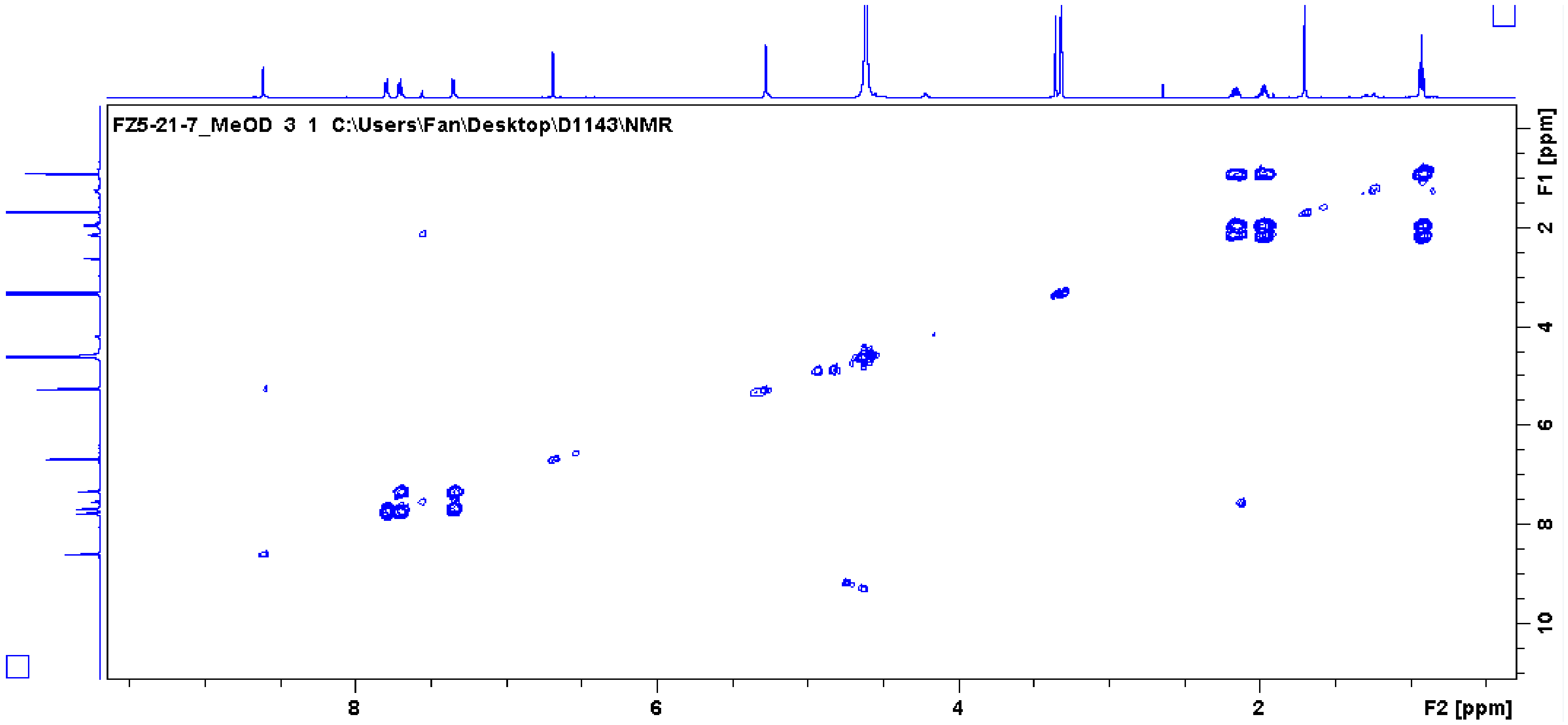

Figure S26. gHSQC spectrum of actinomycetoquinone D (**4**; 150 MHz, $CD_3OD:CDCl_3$ 1:1)

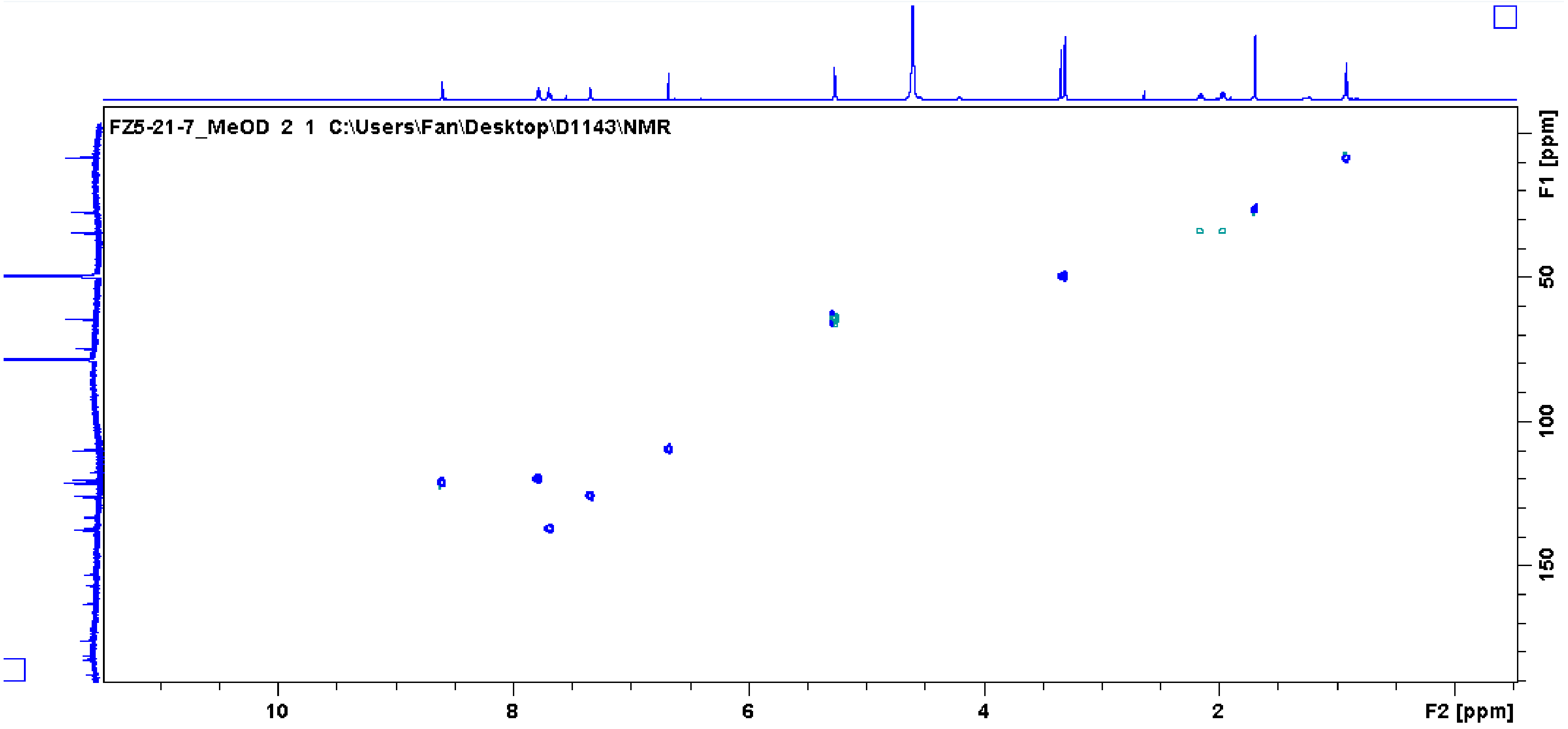

Figure S27. gHMBC spectrum of actinomycetoquinone D (**4**; 150 MHz, $CD_3OD:CDCl_3$ 1:1)

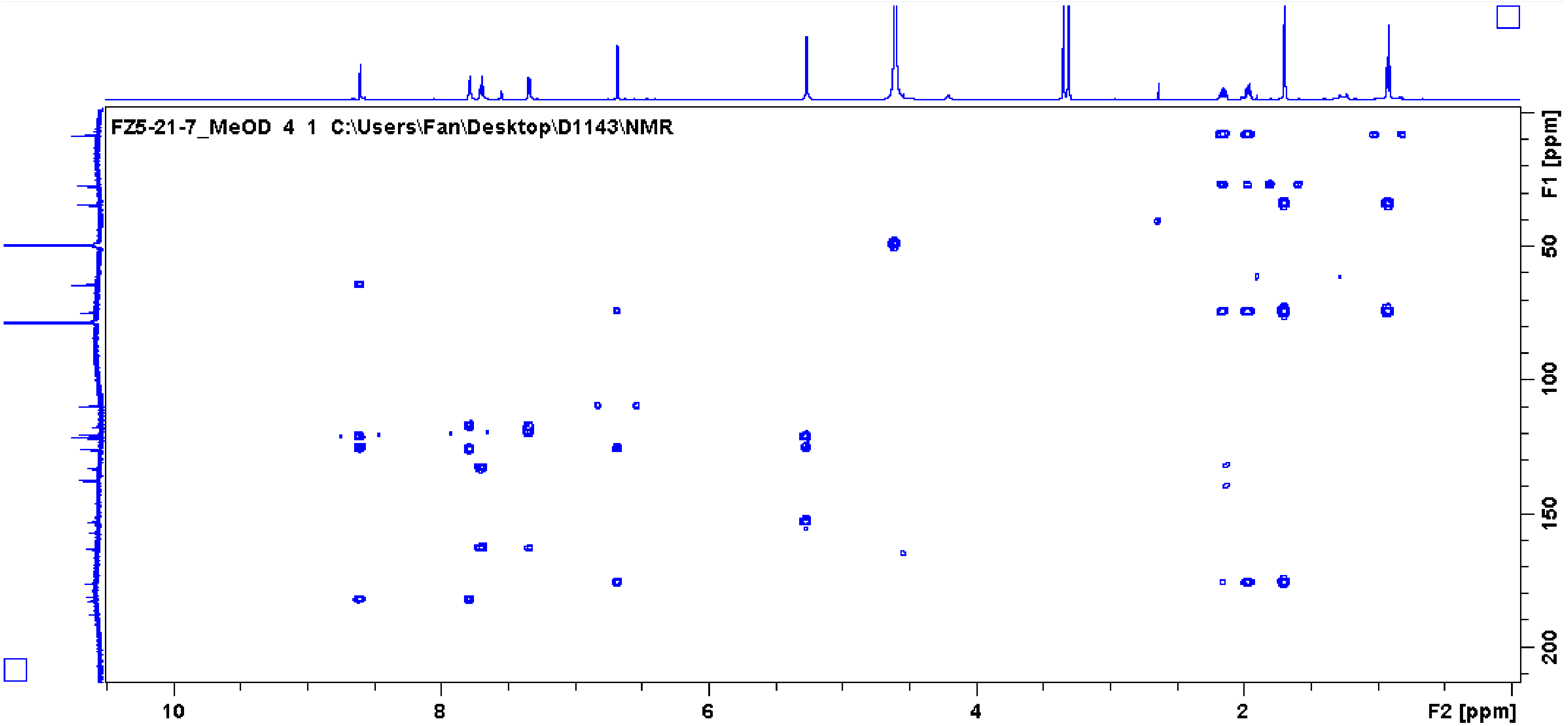

Figure S28. Positive ion HRESIMS of actinomycetoquinone D (**4**)

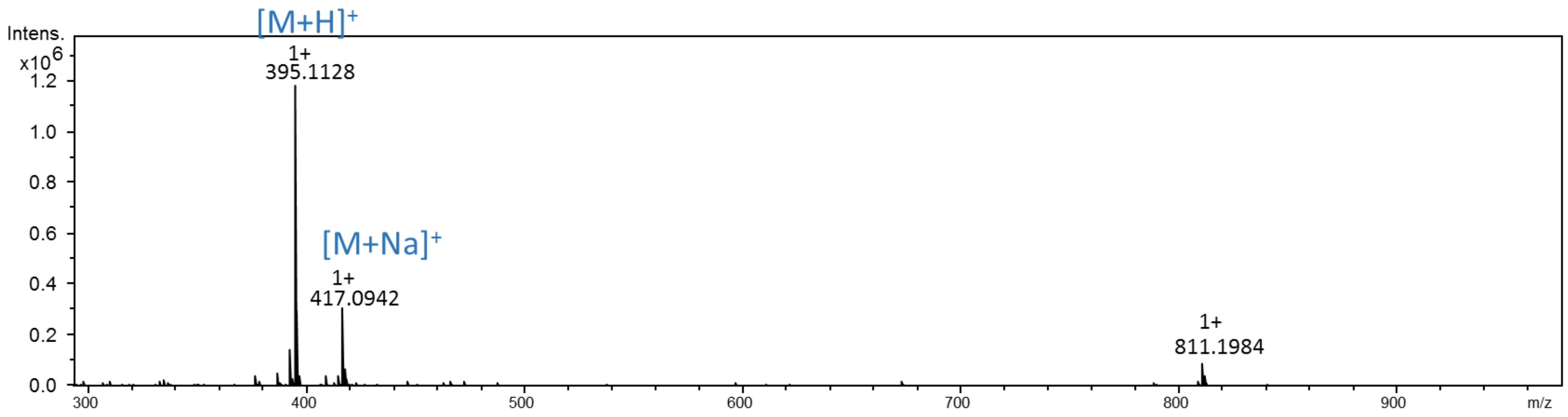

Figure S29. ECD spectrum of actinomycetoquinone D (**4**)

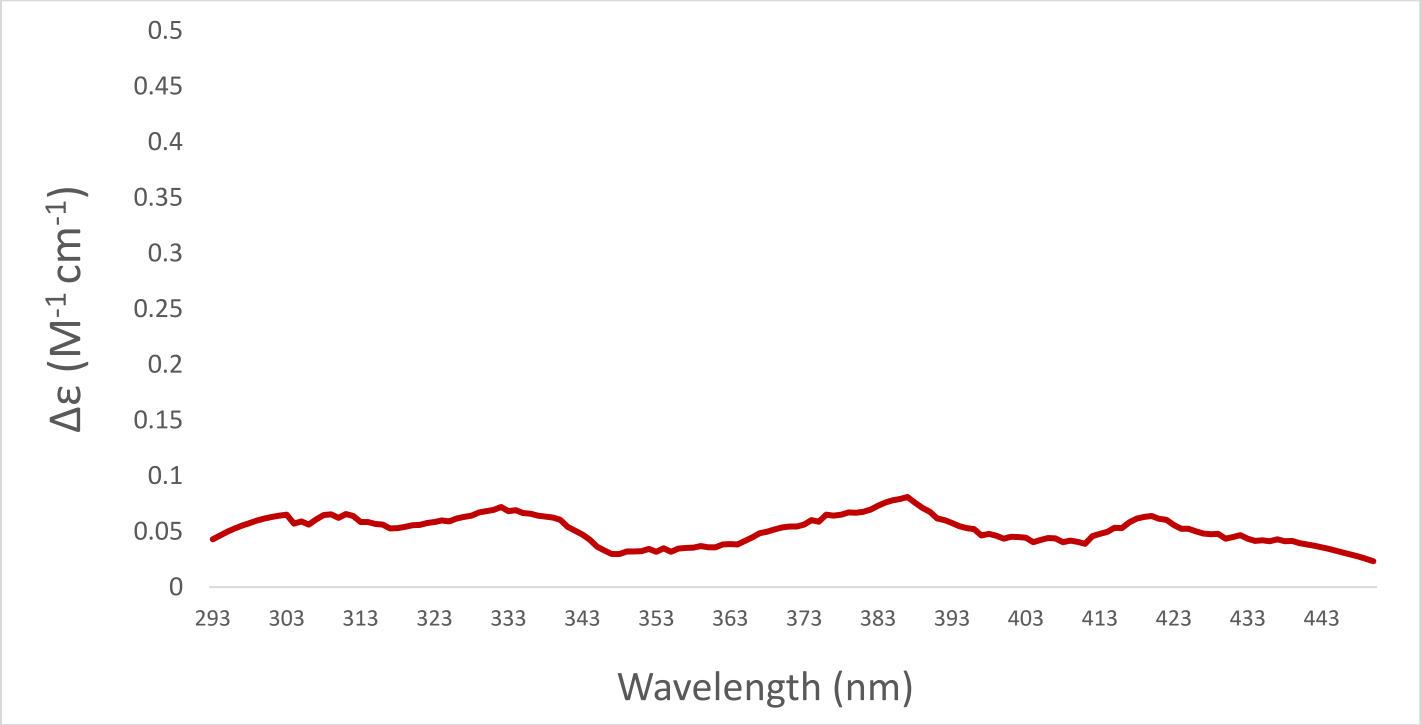

Figure S30. ^{1}H NMR spectrum of actinomycetoquinone E (**5**; 600 MHz, $CD_3OD:CDCl_3$ 1:1)

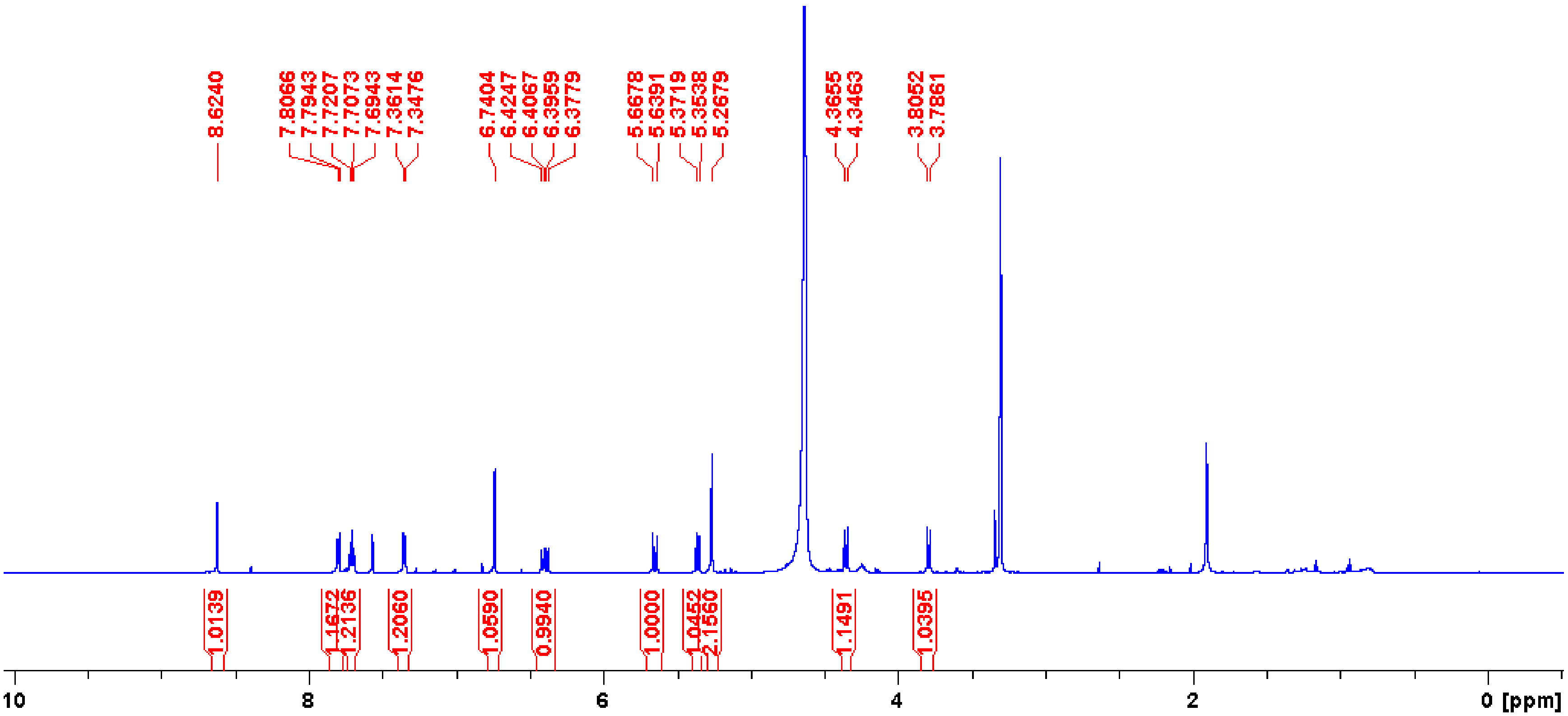

Figure S31. ^{13}C NMR spectrum of actinomycetoquinone E (**5**; 150 MHz, CD_3OD:$CDCl_3$ 1:1)

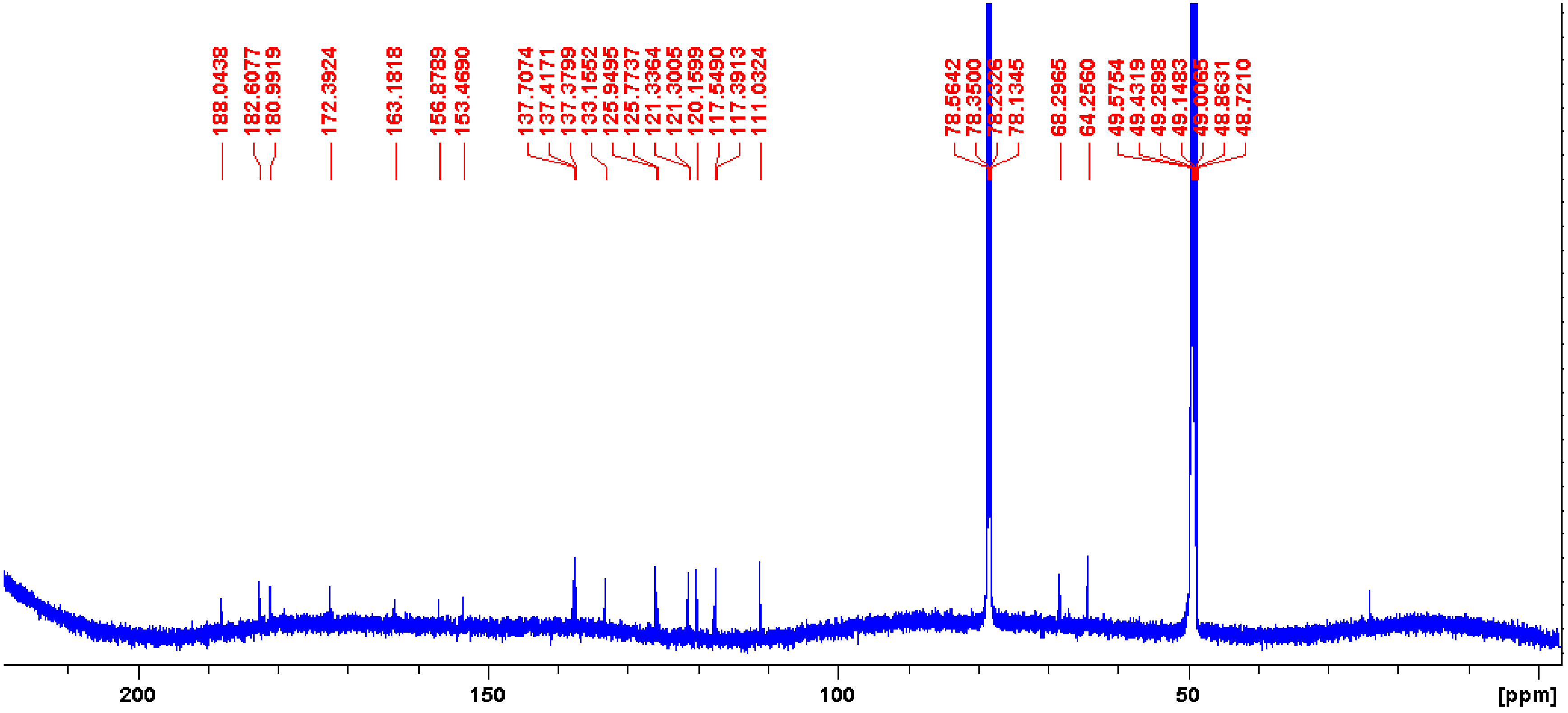

Figure S32. gCOSY spectrum of actinomycetoquinone E (**5**; 150 MHz, $CD_3OD:CDCl_3$ 1:1)

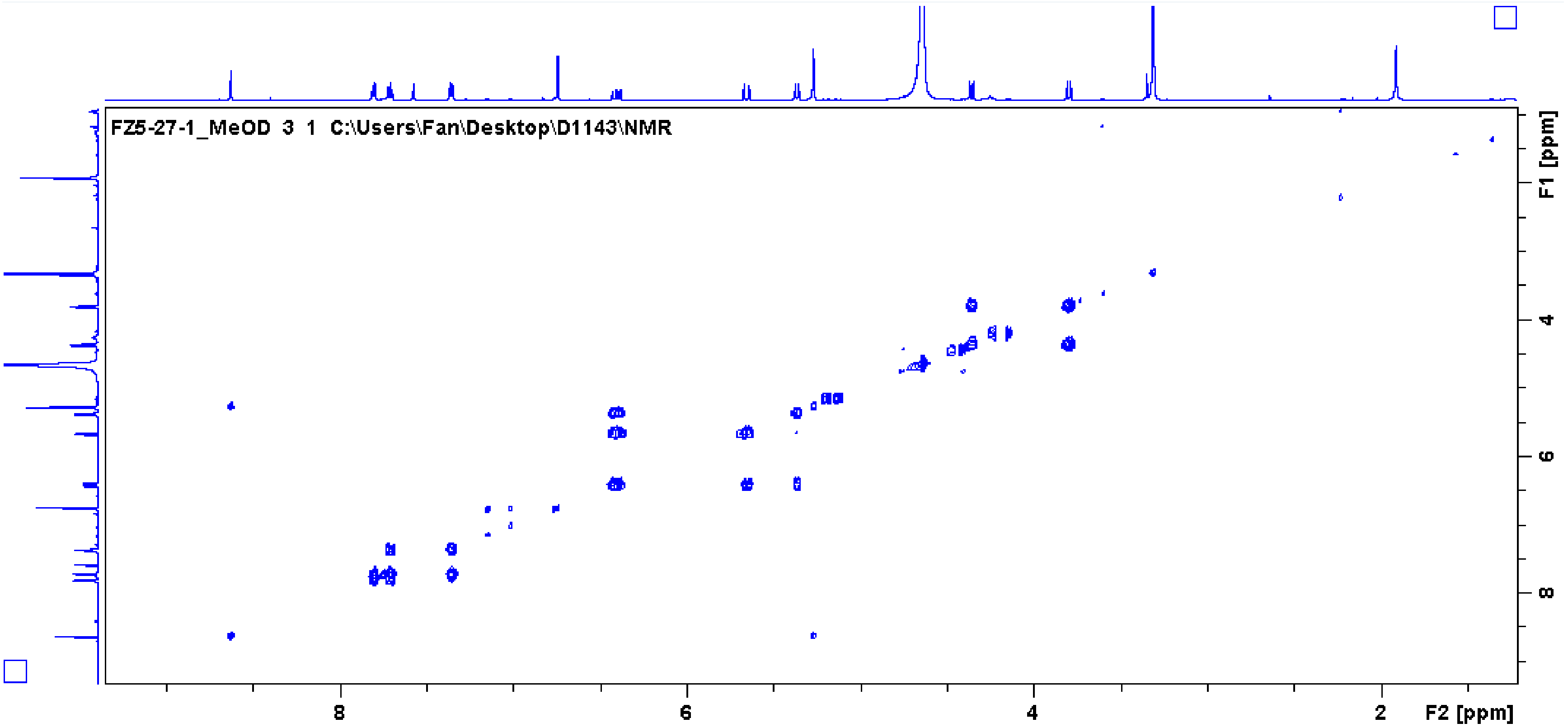

Figure S33. gHSQC spectrum of actinomycetoquinone E (**5**; 150 MHz, CD_3OD:$CDCl_3$ 1:1)

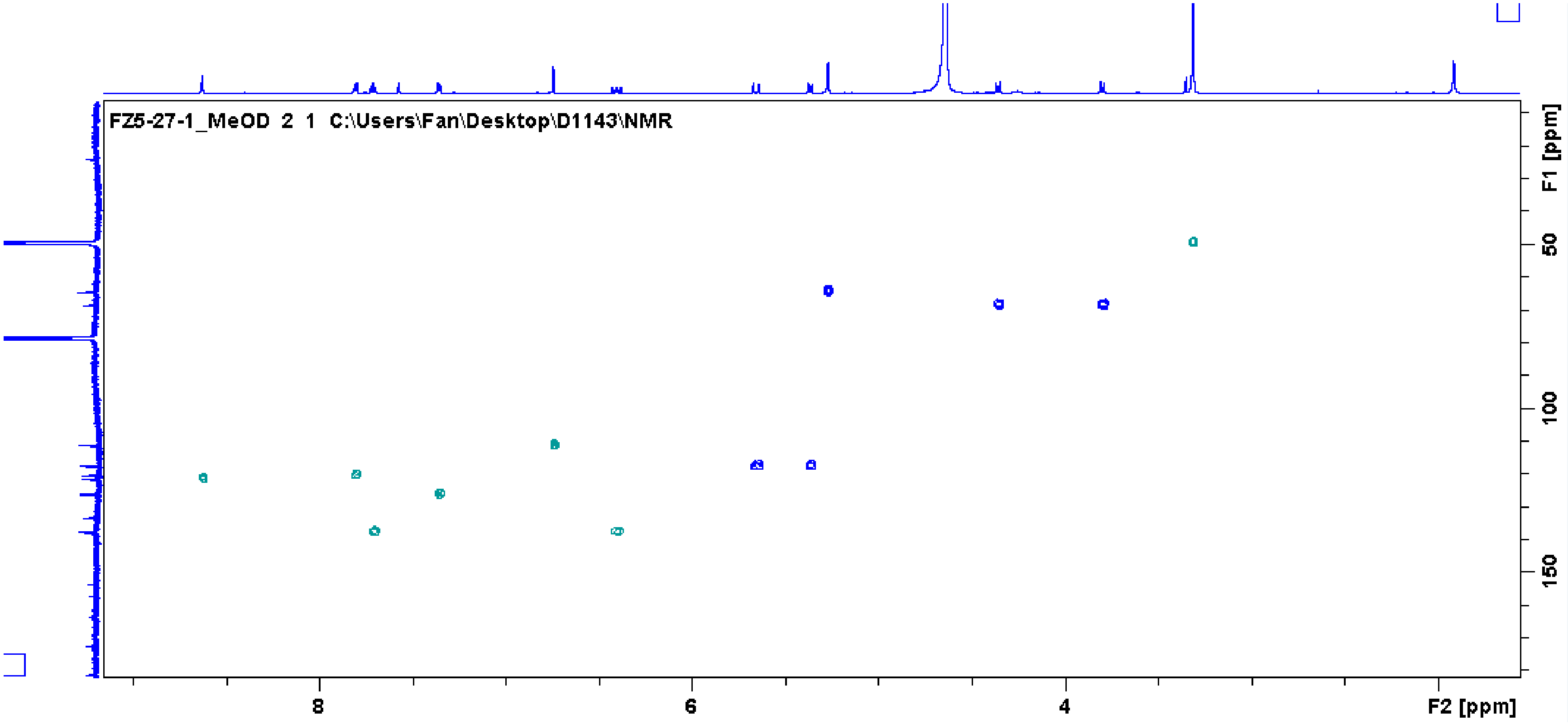

Figure S34. gHMBC spectrum of actinomycetoquinone E (**5**; 150 MHz, CD_3OD:$CDCl_3$ 1:1)

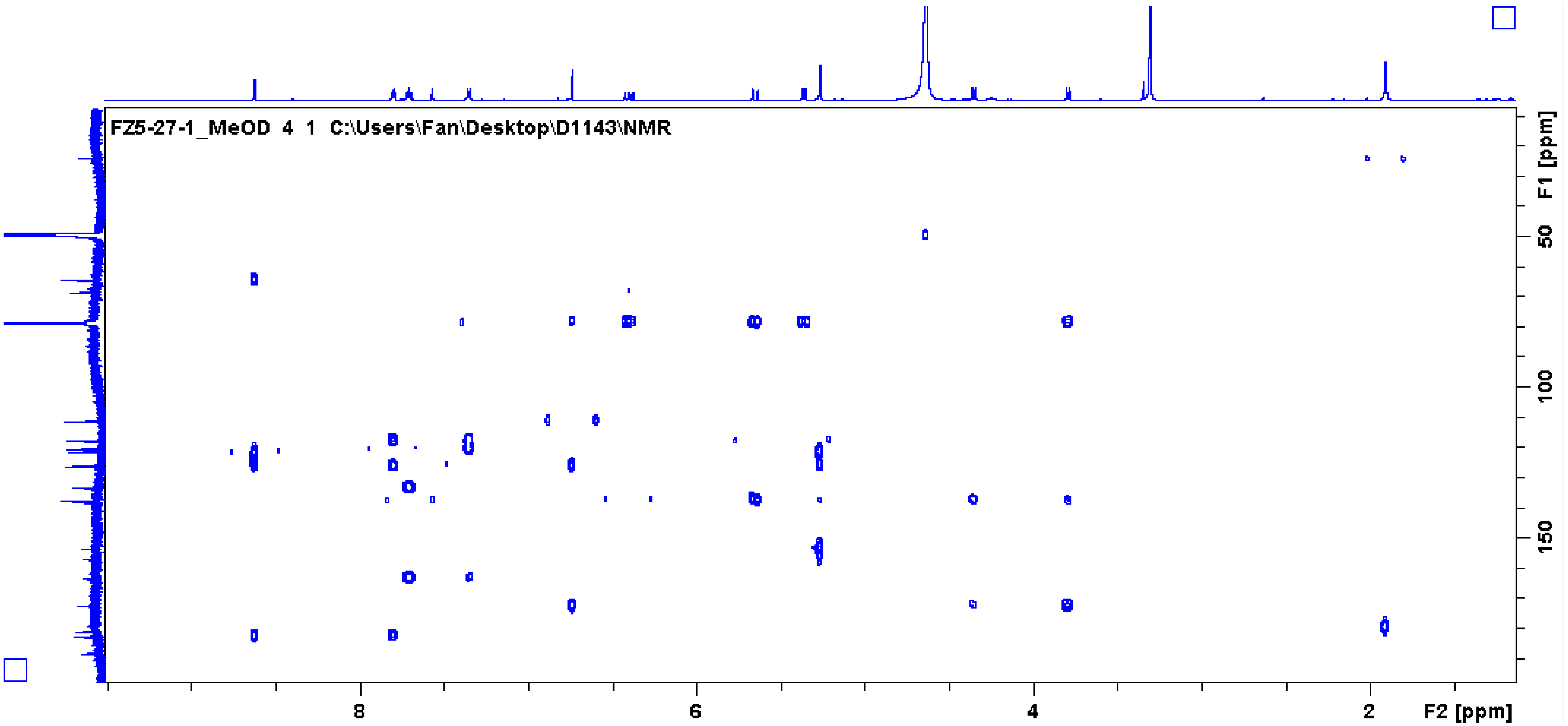

Figure S35. Positive ion HRESIMS of actinomycetoquinone E (**5**)

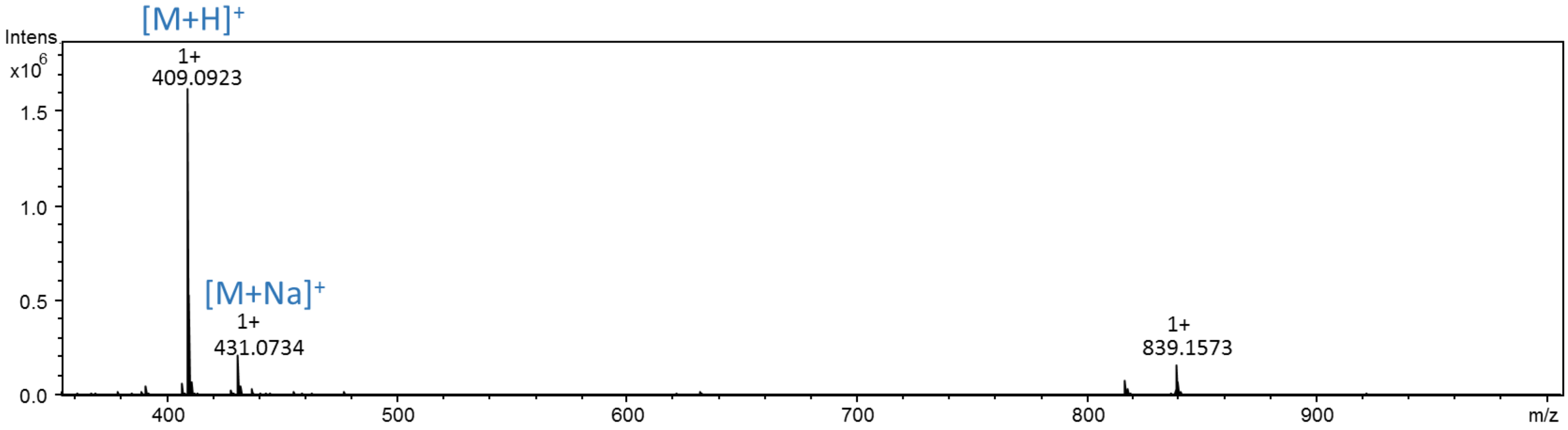

Figure S36. ECD spectrum of actinomycetoquinone E (**5**)

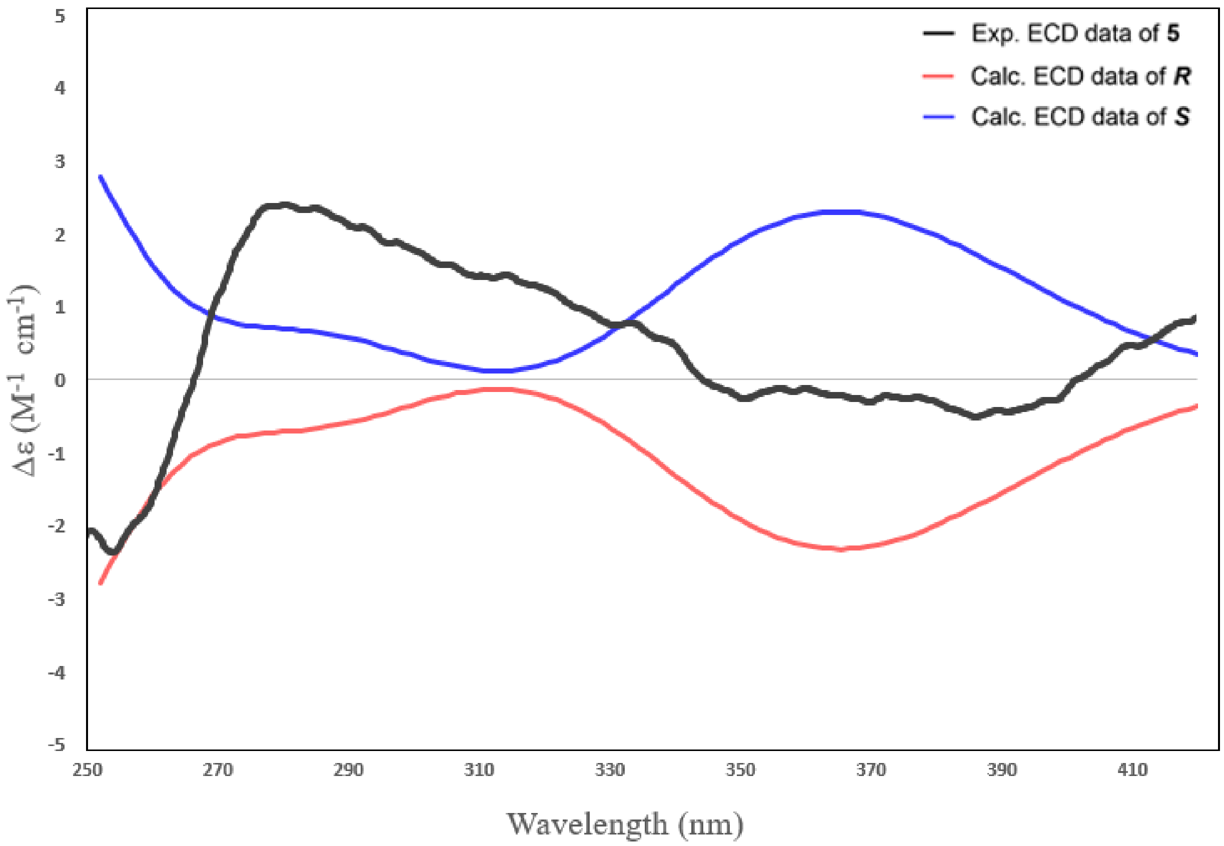

Figure S37. A molecular drawing of actinomycetoquinone A (**1**) shown with 50% probability ellipsoids and selected atom labels. All H atoms bound to C atoms (except those bound to atoms C2 and C2A) are omitted

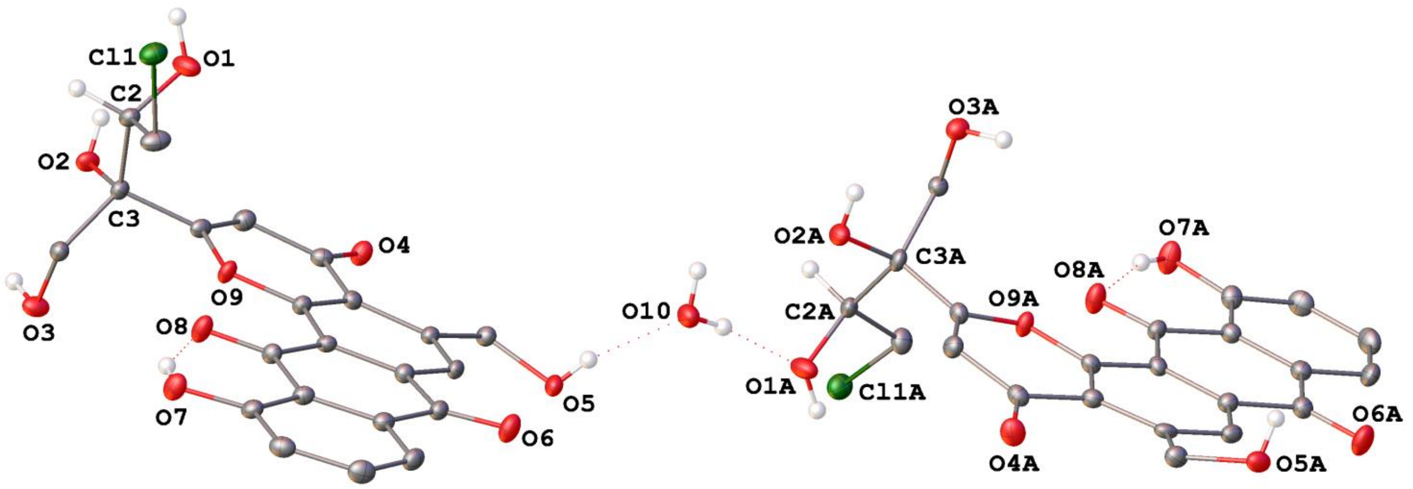

Figure S38. A molecular overlay of the two symmetry-independent molecules in actinomycetoquinone A (**1**) shown with 50% probability ellipsoids and selected atom labels

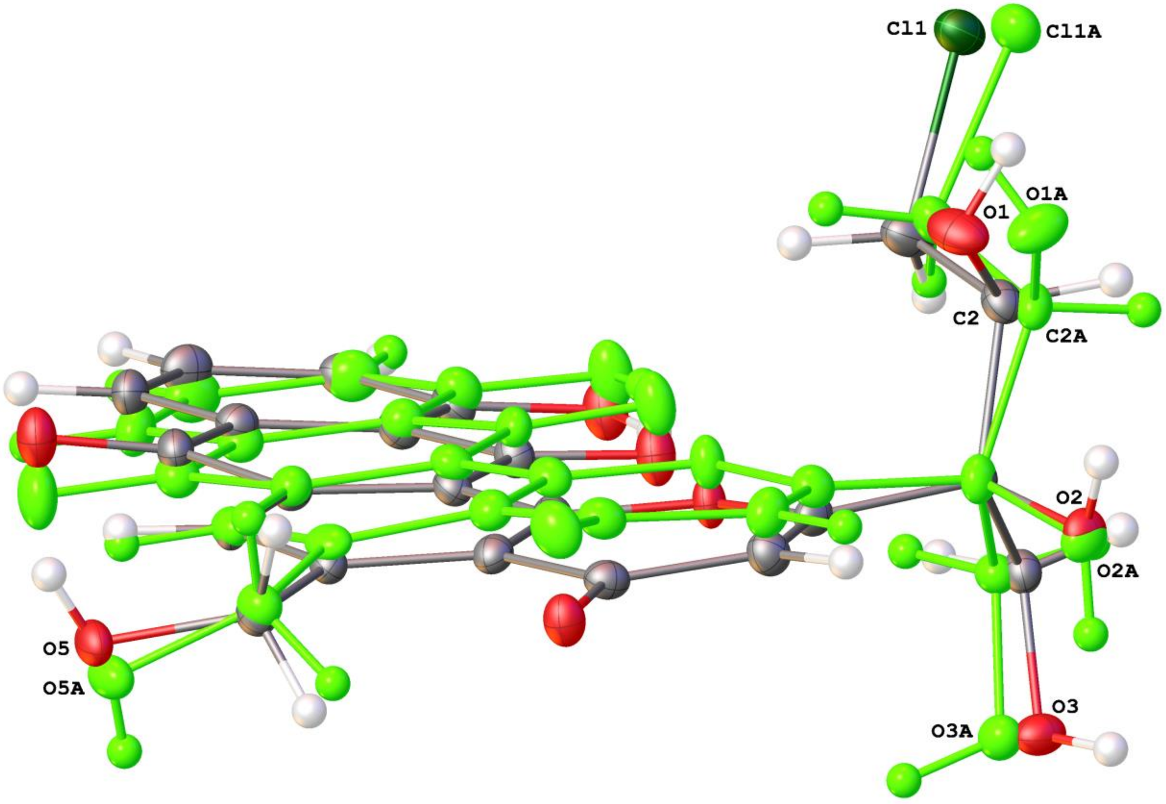

Table S1. Crystal data and structure refinement for actinomycetoquinone A (**1**).

Empirical formula	$[C_{22}H_{18}O_9Cl] \cdot 0.5\ H_2O$
Formula weight	469.81
Temperature/K	100.0
Crystal system	triclinic
Space group	*P*1
a/Å	6.9698(8)
b/Å	11.2605(16)
c/Å	12.5702(16)
α/°	90.932(7)
β/°	99.055(6)
γ/°	99.003(10)
Volume/Å^3	961.4(2)
Z	2
ρ_{calc}g/cm^3	1.623
μ/mm^{-1}	2.313
F(000)	486.0
Crystal size/mm^3	0.11 × 0.034 × 0.03
Radiation	CuKα (λ = 1.54178)
2Θ range for data collection/°	7.128 to 159.1
Index ranges	$-8 \leq h \leq 8, -14 \leq k \leq 13, -15 \leq l \leq 15$
Reflections collected	43505
Independent reflections	7848 [R_{int} = 0.0487, R_{sigma} = 0.0342]
Data/restraints/parameters	7848/4/623
Goodness-of-fit on F^2	1.042
Final R indexes [I>=2σ (I)]	R_1 = 0.0322, wR_2 = 0.0808
Final R indexes [all data]	R_1 = 0.0340, wR_2 = 0.0817
Largest diff. peak/hole / e Å^{-3}	0.29/-0.28
Flack parameter	0.014(13)

Table S2. Fractional atomic coordinates (×10[4]) and equivalent isotropic displacement parameters (Å[2]×10[3]) for actinomycetoquinone A (**1**). U_{eq} is defined as 1/3 of of the trace of the orthogonalised U_{IJ}.

**Atom**	***x***	***y***	***z***	**U(eq)**
Cl1	2412.4(10)	12983.3(7)	789.8(6)	20.12(16)
O1	3987(3)	13101(2)	3286.4(19)	22.3(5)
O2	7721(3)	14341(2)	4053.1(18)	15.9(4)
O3	10834(3)	14222(2)	3077.7(19)	18.7(5)
O4	7836(3)	10416(2)	5845.5(17)	17.0(4)
O5	8145(3)	6870(2)	5227.3(18)	16.6(4)
O6	7028(3)	6333(2)	1435.2(18)	20.4(5)
O7	8128(3)	10708(2)	-1205.5(18)	19.4(5)
O8	7886(3)	11083(2)	772.4(18)	19.3(5)
O9	7687(3)	11485.8(19)	2762.3(16)	14.2(4)
C1	4777(4)	12768(3)	1510(3)	21.4(6)
C2	5264(4)	13524(3)	2553(2)	14.9(6)
C3	7373(4)	13514(3)	3160(2)	13.1(6)
C4	8924(4)	13945(3)	2446(2)	14.3(6)
C5	7576(4)	12272(3)	3570(2)	13.7(6)
C6	7600(4)	11939(3)	4594(2)	15.9(6)
C7	7736(4)	10725(3)	4890(2)	14.4(6)
C8	7719(4)	9858(3)	3995(2)	13.7(6)
C9	7711(4)	8610(3)	4136(2)	12.8(6)
C10	7846(4)	8080(3)	5251(2)	14.0(6)
C11	7568(4)	7852(3)	3243(2)	13.3(5)
C12	7535(4)	8311(3)	2208(2)	13.6(6)
C13	7333(4)	7406(3)	1288(3)	14.5(6)
C14	7484(4)	7879(3)	203(3)	14.5(6)
C15	7396(4)	7057(3)	-654(3)	16.8(6)
C16	7560(5)	7491(3)	-1683(3)	19.4(6)
C17	7801(5)	8711(3)	-1849(3)	17.4(6)
C18	7887(4)	9540(3)	-991(3)	15.3(6)
C19	7710(4)	9123(3)	53(2)	13.7(6)
C20	7754(4)	10003(3)	943(2)	13.5(6)
C21	7654(4)	9533(3)	2040(2)	12.8(6)
C22	7700(4)	10300(3)	2953(2)	13.4(6)
Cl1A	7647.8(10)	3747.2(7)	9261.2(6)	21.07(17)
O1A	6002(3)	3701(2)	6810.8(19)	22.4(5)
O2A	2161(3)	3559(2)	6060.2(18)	16.8(4)
O3A	-842(3)	2541(2)	7031.8(17)	17.0(4)
O4A	3340(3)	-288(2)	4297.1(17)	20.5(5)
O5A	1476(3)	-3903(2)	4687.9(18)	17.9(4)
O6A	2355(4)	-4347(2)	8447.9(19)	24.3(5)
O7A	2437(4)	11(2)	11270.9(19)	22.0(5)
O8A	2847(4)	397(2)	9313.9(18)	20.6(5)

O9A	2873(3)	796.7(19)	7318.4(17)	15.2(4)
C1A	5454(4)	2844(3)	8527(2)	18.3(6)
C2A	4657(4)	3501(3)	7554(3)	15.3(6)
C3A	2650(5)	2824(3)	6941(3)	15.1(6)
C4A	1070(4)	2749(3)	7679(2)	14.1(6)
C5A	2832(4)	1591(3)	6504(2)	14.3(6)
C6A	2944(4)	1262(3)	5483(3)	17.0(6)
C7A	3084(4)	26(3)	5195(2)	15.6(6)
C8A	2891(4)	-843(3)	6066(2)	14.7(6)
C9A	2643(4)	-2106(3)	5870(3)	14.5(6)
C10A	2321(4)	-2668(3)	4732(2)	16.5(6)
C11A	2542(4)	-2854(3)	6731(3)	15.4(6)
C12A	2602(4)	-2392(3)	7774(2)	14.5(6)
C13A	2450(4)	-3275(3)	8647(3)	16.6(6)
C14A	2402(4)	-2802(3)	9752(3)	16.1(6)
C15A	2266(5)	-3613(3)	10576(3)	19.4(6)
C16A	2241(5)	-3183(3)	11621(3)	21.8(7)
C17A	2328(5)	-1964(3)	11837(3)	19.4(7)
C18A	2430(4)	-1141(3)	11012(2)	16.3(6)
C19A	2506(4)	-1562(3)	9951(2)	14.3(6)
C20A	2701(4)	-691(3)	9102(3)	13.5(6)
C21A	2737(4)	-1156(3)	7990(2)	13.1(6)
C22A	2856(4)	-397(3)	7108(2)	13.4(6)
O10	5074(3)	5194(2)	5201(2)	21.7(5)

Table S3. Anisotropic displacement parameters ($Å^2 \times 10^3$) for actinomycetoquinone A (**1**). The anisotropic displacement factor exponent takes the form: $-2\pi^2[h^2a^{*2}U_{11}+2hka^*b^*U_{12}+\ldots]$

**Atom**	**U_{11}**	**U_{22}**	**U_{33}**	**U_{23}**	**U_{13}**	**U_{12}**
Cl1	18.3(3)	21.2(4)	20.5(4)	-2.6(3)	-1.0(3)	6.4(3)
O1	15.2(11)	30.4(14)	23.9(12)	8.4(10)	7.4(9)	6.7(10)
O2	16.6(10)	14.9(11)	16.9(10)	-4.5(8)	4.0(8)	3.9(8)
O3	13.4(10)	21.3(12)	21.0(11)	-5.4(9)	2.0(9)	2.4(8)
O4	22.7(11)	14.3(11)	14.4(10)	0.6(8)	2.8(8)	4.5(8)
O5	16.4(10)	11.8(11)	22.4(11)	4.3(8)	4.2(9)	3.0(8)
O6	29.7(12)	13.0(11)	19.3(11)	0.8(8)	4.4(9)	5.1(9)
O7	28.9(12)	16.9(11)	14.2(11)	2.5(9)	6.4(9)	5.7(9)
O8	30.9(12)	13.3(11)	16.1(10)	1.6(8)	8.0(9)	6.3(9)
O9	21.8(10)	9.6(10)	12.5(10)	0.1(8)	4.3(8)	4.8(8)
C1	17.0(14)	24.1(17)	22.7(16)	-4.8(13)	-1.2(12)	7.0(12)
C2	14.7(13)	14.5(15)	16.8(14)	1.0(11)	4.7(11)	4.5(11)
C3	14.1(13)	10.1(14)	15.3(14)	-0.8(11)	2.4(11)	2.1(10)
C4	15.1(14)	12.4(14)	16.1(14)	1.3(11)	3.9(11)	3.1(11)
C5	13.2(12)	12.5(15)	15.0(14)	-4.1(11)	1.4(10)	2.4(10)
C6	17.0(14)	15.6(15)	15.5(14)	-1.0(11)	2.5(11)	4.3(11)
C7	12.3(13)	17.1(15)	13.9(15)	-1.3(11)	1.8(11)	3.2(11)
C8	11.8(12)	12.8(14)	16.5(14)	-0.5(11)	3.5(10)	0.9(10)
C9	11.3(12)	11.4(14)	15.8(15)	0.5(11)	2.3(10)	2.0(10)
C10	14.9(13)	13.5(14)	14.1(13)	2.0(11)	3.0(10)	3.1(11)
C11	13.6(13)	9.2(13)	17.7(15)	0.5(11)	3.4(11)	2.2(10)
C12	13.4(13)	13.0(15)	14.1(14)	-0.4(11)	2.0(11)	1.6(11)
C13	13.9(13)	10.9(14)	18.7(15)	0.6(11)	2.7(11)	2.5(10)
C14	12.4(13)	15.0(15)	16.2(14)	-1.0(11)	1.9(11)	2.8(11)
C15	18.1(14)	11.5(15)	20.7(16)	-1.8(12)	1.9(12)	3.5(11)
C16	20.0(15)	21.5(17)	16.5(15)	-5.5(12)	2.9(12)	3.7(12)
C17	15.9(14)	22.7(17)	14.3(15)	1.3(12)	3.3(11)	4.7(12)
C18	14.5(13)	16.1(15)	16.3(14)	1.7(12)	2.9(11)	5.7(11)
C19	13.7(13)	15.0(15)	13.3(14)	0.0(11)	2.8(10)	4.3(11)
C20	15.5(13)	13.5(15)	12.3(14)	1.6(11)	3.0(11)	3.6(11)
C21	11.3(12)	12.8(15)	14.9(14)	0.8(11)	2.6(10)	3.5(10)
C22	13.6(13)	12.4(14)	14.7(14)	0.0(11)	3.0(11)	2.8(10)
Cl1A	20.3(3)	18.5(4)	22.7(4)	-2.5(3)	-0.1(3)	1.6(3)
O1A	16.3(11)	30.5(14)	23.5(12)	10.8(10)	8.1(9)	7.3(10)
O2A	16.4(10)	17.2(11)	17.8(11)	6.6(8)	3.9(8)	4.1(8)
O3A	16.7(11)	15.9(11)	18.1(11)	-1.3(8)	1.5(8)	3.6(8)
O4A	26.3(11)	19.9(12)	15.6(11)	-2.3(8)	6.7(9)	1.3(9)
O5A	15.2(10)	16.4(11)	21.7(11)	-5.7(8)	3.6(9)	1.6(8)
O6A	36.5(14)	13.0(12)	25.4(12)	0.0(9)	9.3(10)	5.8(10)
O7A	38.6(14)	15.4(12)	12.0(11)	0.1(9)	5.5(10)	3.3(9)
O8A	33.1(12)	12.6(11)	16.5(11)	-0.5(8)	6.9(9)	2.1(9)
O9A	23.7(11)	9.6(10)	13.2(10)	1.0(8)	5.1(8)	2.9(8)

C1A	19.7(15)	14.6(15)	18.7(14)	0.0(11)	0.5(11)	-0.3(11)
C2A	16.1(14)	11.1(14)	20.0(14)	2.4(11)	6.1(11)	3.0(11)
C3A	18.8(14)	10.5(15)	17.2(14)	3.5(11)	4.9(11)	3.2(11)
C4A	12.8(13)	13.2(15)	16.0(14)	-0.1(11)	2.2(11)	1.4(10)
C5A	14.3(13)	14.0(15)	14.5(14)	2.4(11)	3.7(11)	0.6(11)
C6A	19.1(14)	15.2(15)	18.3(15)	4.9(11)	5.9(11)	4.7(11)
C7A	13.5(13)	17.4(15)	15.4(14)	1.6(11)	3.0(11)	0.2(11)
C8A	13.0(13)	15.5(15)	16.2(14)	0.7(11)	3.4(11)	2.9(11)
C9A	9.3(13)	17.8(16)	17.1(15)	-2.8(11)	3.8(11)	2.6(11)
C10A	16.3(13)	15.9(15)	17.3(15)	-1.6(11)	3.1(11)	2.2(11)
C11A	12.4(13)	13.3(14)	20.5(15)	-2.2(11)	2.8(11)	2.7(10)
C12A	13.7(13)	13.6(15)	16.3(15)	0.6(11)	2.4(11)	2.7(11)
C13A	15.4(13)	15.4(15)	19.2(15)	1.4(12)	3.9(11)	1.7(11)
C14A	13.2(13)	16.3(16)	18.9(15)	1.0(12)	3.8(11)	1.7(11)
C15A	20.8(15)	13.7(15)	25.3(17)	5.3(12)	5.7(12)	4.9(11)
C16A	21.2(16)	23.9(18)	20.6(16)	9.7(13)	4.4(13)	2.6(13)
C17A	21.3(16)	23.7(18)	13.3(15)	2.0(13)	3.4(12)	3.4(13)
C18A	17.3(14)	15.6(16)	15.0(14)	0.8(11)	1.5(11)	0.4(11)
C19A	14.4(13)	15.0(15)	13.9(14)	1.5(11)	2.7(11)	2.8(11)
C20A	13.5(13)	10.8(14)	16.4(15)	-0.4(11)	2.2(11)	2.6(10)
C21A	10.8(12)	13.7(15)	14.0(14)	0.7(11)	1.8(10)	-0.3(10)
C22A	12.4(13)	14.2(15)	13.7(14)	-0.6(11)	4.0(10)	0.5(10)
O10	19.3(11)	19.8(12)	28.3(13)	8.4(9)	8.8(9)	4.0(9)

Table S4. Bond lengths for actinomycetoquinone A (**1**)

Atom	Atom	Length/Å		Atom	Atom	Length/Å
Cl1	C1	1.805(3)		Cl1A	C1A	1.806(3)
O1	C2	1.417(4)		O1A	C2A	1.421(4)
O2	C3	1.413(4)		O2A	C3A	1.424(4)
O3	C4	1.425(4)		O3A	C4A	1.430(4)
O4	C7	1.250(4)		O4A	C7A	1.225(4)
O5	C10	1.411(4)		O5A	C10A	1.419(4)
O6	C13	1.216(4)		O6A	C13A	1.218(4)
O7	C18	1.337(4)		O7A	C18A	1.331(4)
O8	C20	1.230(4)		O8A	C20A	1.235(4)
O9	C5	1.356(3)		O9A	C5A	1.370(4)
O9	C22	1.362(4)		O9A	C22A	1.364(4)
C1	C2	1.514(4)		C1A	C2A	1.515(4)
C2	C3	1.547(4)		C2A	C3A	1.562(4)
C3	C4	1.538(4)		C3A	C4A	1.541(4)
C3	C5	1.517(4)		C3A	C5A	1.515(4)
C5	C6	1.345(4)		C5A	C6A	1.347(4)
C6	C7	1.436(4)		C6A	C7A	1.455(5)
C7	C8	1.476(4)		C7A	C8A	1.488(4)
C8	C9	1.418(4)		C8A	C9A	1.418(5)
C8	C22	1.407(4)		C8A	C22A	1.400(4)
C9	C10	1.527(4)		C9A	C10A	1.525(4)
C9	C11	1.380(4)		C9A	C11A	1.385(5)
C11	C12	1.407(4)		C11A	C12A	1.395(4)
C12	C13	1.503(4)		C12A	C13A	1.498(4)
C12	C21	1.387(4)		C12A	C21A	1.399(4)
C13	C14	1.485(4)		C13A	C14A	1.486(4)
C14	C15	1.396(4)		C14A	C15A	1.398(4)
C14	C19	1.405(4)		C14A	C19A	1.402(5)
C15	C16	1.405(5)		C15A	C16A	1.396(5)
C16	C17	1.382(5)		C16A	C17A	1.384(5)
C17	C18	1.402(4)		C17A	C18A	1.405(4)
C18	C19	1.418(4)		C18A	C19A	1.420(4)
C19	C20	1.476(4)		C19A	C20A	1.469(4)
C20	C21	1.492(4)		C20A	C21A	1.490(4)
C21	C22	1.419(4)		C21A	C22A	1.416(4)

Table S5. Bond angles for actinomycetoquinone A (**1**).

Atom	Atom	Atom	Angle/°		Atom	Atom	Atom	Angle/°
C5	O9	C22	120.5(2)		C22A	O9A	C5A	120.8(2)
C2	C1	Cl1	110.1(2)		C2A	C1A	Cl1A	109.9(2)
O1	C2	C1	111.2(3)		O1A	C2A	C1A	112.2(3)
O1	C2	C3	105.7(2)		O1A	C2A	C3A	108.5(2)
C1	C2	C3	113.6(2)		C1A	C2A	C3A	112.6(2)
O2	C3	C2	108.4(2)		O2A	C3A	C2A	104.4(2)
O2	C3	C4	106.6(2)		O2A	C3A	C4A	109.7(2)
O2	C3	C5	108.5(2)		O2A	C3A	C5A	108.9(3)
C4	C3	C2	111.2(2)		C4A	C3A	C2A	109.9(2)
C5	C3	C2	110.5(2)		C5A	C3A	C2A	111.9(2)
C5	C3	C4	111.5(2)		C5A	C3A	C4A	111.7(2)
O3	C4	C3	110.8(2)		O3A	C4A	C3A	109.3(2)
O9	C5	C3	111.7(2)		O9A	C5A	C3A	109.9(2)
C6	C5	O9	122.0(3)		C6A	C5A	O9A	122.3(3)
C6	C5	C3	126.2(3)		C6A	C5A	C3A	127.9(3)
C5	C6	C7	121.7(3)		C5A	C6A	C7A	120.8(3)
O4	C7	C6	122.2(3)		O4A	C7A	C6A	122.7(3)
O4	C7	C8	121.9(3)		O4A	C7A	C8A	121.8(3)
C6	C7	C8	115.9(3)		C6A	C7A	C8A	115.5(3)
C9	C8	C7	123.2(3)		C9A	C8A	C7A	122.5(3)
C22	C8	C7	117.8(3)		C22A	C8A	C7A	118.6(3)
C22	C8	C9	119.0(3)		C22A	C8A	C9A	118.8(3)
C8	C9	C10	121.8(3)		C8A	C9A	C10A	121.9(3)
C11	C9	C8	119.2(3)		C11A	C9A	C8A	119.0(3)
C11	C9	C10	119.0(3)		C11A	C9A	C10A	118.9(3)
O5	C10	C9	112.2(2)		O5A	C10A	C9A	112.2(3)
C9	C11	C12	120.8(3)		C9A	C11A	C12A	121.3(3)
C11	C12	C13	116.6(3)		C11A	C12A	C13A	117.4(3)
C21	C12	C11	121.8(3)		C11A	C12A	C21A	121.3(3)
C21	C12	C13	121.6(3)		C21A	C12A	C13A	121.3(3)
O6	C13	C12	120.6(3)		O6A	C13A	C12A	120.5(3)
O6	C13	C14	122.0(3)		O6A	C13A	C14A	121.5(3)
C14	C13	C12	117.3(3)		C14A	C13A	C12A	117.9(3)
C15	C14	C13	118.3(3)		C15A	C14A	C13A	118.6(3)
C15	C14	C19	121.4(3)		C15A	C14A	C19A	121.3(3)
C19	C14	C13	120.3(3)		C19A	C14A	C13A	120.0(3)
C14	C15	C16	119.0(3)		C16A	C15A	C14A	119.4(3)
C17	C16	C15	120.7(3)		C17A	C16A	C15A	120.4(3)
C16	C17	C18	120.5(3)		C16A	C17A	C18A	120.8(3)
O7	C18	C17	117.5(3)		O7A	C18A	C17A	117.5(3)
O7	C18	C19	122.7(3)		O7A	C18A	C19A	123.0(3)
C17	C18	C19	119.9(3)		C17A	C18A	C19A	119.4(3)
C14	C19	C18	118.6(3)		C14A	C19A	C18A	118.6(3)

C14	C19	C20	122.1(3)		C14A	C19A	C20A	122.2(3)
C18	C19	C20	119.3(3)		C18A	C19A	C20A	119.2(3)
O8	C20	C19	120.2(3)		O8A	C20A	C19A	120.4(3)
O8	C20	C21	122.2(3)		O8A	C20A	C21A	121.3(3)
C19	C20	C21	117.7(3)		C19A	C20A	C21A	118.3(3)
C12	C21	C20	120.7(3)		C12A	C21A	C20A	120.2(3)
C12	C21	C22	117.0(3)		C12A	C21A	C22A	117.0(3)
C22	C21	C20	122.3(3)		C22A	C21A	C20A	122.7(3)
O9	C22	C8	121.8(3)		O9A	C22A	C8A	121.3(3)
O9	C22	C21	116.2(3)		O9A	C22A	C21A	116.4(3)
C8	C22	C21	121.9(3)		C8A	C22A	C21A	122.3(3)

Table S6. Hydrogen bonds for actinomycetoquinone A (**1**).

**D**	**H**	**A**	**d(D-H)/Å**	**d(H-A)/Å**	**d(D-A)/Å**	**D-H-A/°**
O1	H1	O3[1]	0.83(5)	1.85(5)	2.681(3)	173(5)
O2	H2	O10[2]	0.75(5)	2.11(5)	2.785(3)	149(4)
O3	H3	O5A[3]	0.86(5)	1.98(5)	2.834(3)	168(4)
O5	H5	O10	0.74(4)	1.89(5)	2.618(3)	170(5)
O7	H7	O8	0.87(4)	1.76(4)	2.551(3)	151(4)
C1	H1A	O9	0.99	2.40	2.943(4)	114.1
O1A	H1AA	O3A[4]	0.80(5)	1.95(5)	2.710(3)	156(4)
O3A	H3A	O4[5]	0.91(4)	1.89(4)	2.749(3)	157(4)
O5A	H5A	O5[5]	0.77(5)	2.07(5)	2.778(3)	152(4)
O7A	H7A	O8A	0.75(5)	1.84(5)	2.557(3)	162(5)
O10	H10E	O1A	0.80(3)	1.92(3)	2.716(3)	173(4)
O10	H10F	O5A[2]	0.79(3)	2.10(4)	2.837(3)	153(4)

Table S7. Torsion angles for actinomycetoquinone A (**1**).

**A**	**B**	**C**	**D**	**Angle/°**		**A**	**B**	**C**	**D**	**Angle/°**
Cl1	C1	C2	O1	67.8(3)		Cl1A	C1A	C2A	O1A	62.6(3)
Cl1	C1	C2	C3	-173.1(2)		Cl1A	C1A	C2A	C3A	-174.6(2)
O1	C2	C3	O2	-63.7(3)		O1A	C2A	C3A	O2A	-54.4(3)
O1	C2	C3	C4	179.4(2)		O1A	C2A	C3A	C4A	-172.0(2)
O1	C2	C3	C5	55.1(3)		O1A	C2A	C3A	C5A	63.3(3)
O2	C3	C4	O3	47.4(3)		O2A	C3A	C4A	O3A	45.0(3)
O2	C3	C5	O9	-166.6(2)		O2A	C3A	C5A	O9A	-166.9(2)
O2	C3	C5	C6	15.0(4)		O2A	C3A	C5A	C6A	13.2(4)
O4	C7	C8	C9	3.4(4)		O4A	C7A	C8A	C9A	-11.3(4)
O4	C7	C8	C22	-177.0(3)		O4A	C7A	C8A	C22A	171.9(3)
O6	C13	C14	C15	-4.2(4)		O6A	C13A	C14A	C15A	0.7(4)
O6	C13	C14	C19	175.9(3)		O6A	C13A	C14A	C19A	-179.7(3)
O7	C18	C19	C14	-179.3(3)		O7A	C18A	C19A	C14A	-177.2(3)
O7	C18	C19	C20	1.2(4)		O7A	C18A	C19A	C20A	3.1(5)
O8	C20	C21	C12	-179.1(3)		O8A	C20A	C21A	C12A	178.9(3)
O8	C20	C21	C22	0.2(4)		O8A	C20A	C21A	C22A	-2.7(4)
O9	C5	C6	C7	-0.3(4)		O9A	C5A	C6A	C7A	1.1(5)
C1	C2	C3	O2	174.1(3)		C1A	C2A	C3A	O2A	-179.2(2)
C1	C2	C3	C4	57.2(3)		C1A	C2A	C3A	C4A	63.2(3)
C1	C2	C3	C5	-67.1(3)		C1A	C2A	C3A	C5A	-61.6(3)
C2	C3	C4	O3	165.4(2)		C2A	C3A	C4A	O3A	159.2(2)
C2	C3	C5	O9	74.7(3)		C2A	C3A	C5A	O9A	78.2(3)
C2	C3	C5	C6	-103.7(3)		C2A	C3A	C5A	C6A	-101.7(4)
C3	C5	C6	C7	177.9(3)		C3A	C5A	C6A	C7A	-179.0(3)
C4	C3	C5	O9	-49.5(3)		C4A	C3A	C5A	O9A	-45.6(3)
C4	C3	C5	C6	132.1(3)		C4A	C3A	C5A	C6A	134.5(3)
C5	O9	C22	C8	-3.9(4)		C5A	O9A	C22A	C8A	3.1(4)
C5	O9	C22	C21	175.0(2)		C5A	O9A	C22A	C21A	-174.9(3)
C5	C3	C4	O3	-70.9(3)		C5A	C3A	C4A	O3A	-75.9(3)
C5	C6	C7	O4	177.2(3)		C5A	C6A	C7A	O4A	-174.3(3)
C5	C6	C7	C8	-3.7(4)		C5A	C6A	C7A	C8A	5.9(4)
C6	C7	C8	C9	-175.7(3)		C6A	C7A	C8A	C9A	168.5(3)
C6	C7	C8	C22	3.9(4)		C6A	C7A	C8A	C22A	-8.3(4)
C7	C8	C9	C10	-3.8(4)		C7A	C8A	C9A	C10A	-7.1(4)
C7	C8	C9	C11	175.9(3)		C7A	C8A	C9A	C11A	177.8(3)
C7	C8	C22	O9	-0.3(4)		C7A	C8A	C22A	O9A	4.1(4)
C7	C8	C22	C21	-179.1(3)		C7A	C8A	C22A	C21A	-178.0(3)
C8	C9	C10	O5	-170.1(3)		C8A	C9A	C10A	O5A	-160.5(3)
C8	C9	C11	C12	3.3(4)		C8A	C9A	C11A	C12A	2.6(4)
C9	C8	C22	O9	179.3(3)		C9A	C8A	C22A	O9A	-172.8(3)
C9	C8	C22	C21	0.6(4)		C9A	C8A	C22A	C21A	5.1(4)
C9	C11	C12	C13	-178.8(3)		C9A	C11A	C12A	C13A	179.0(3)

C9	C11	C12	C21	0.4(4)		C9A	C11A	C12A	C21A	0.9(4)
C10	C9	C11	C12	-177.0(3)		C10A	C9A	C11A	C12A	-172.7(3)
C11	C9	C10	O5	10.3(4)		C11A	C9A	C10A	O5A	14.6(4)
C11	C12	C13	O6	6.7(4)		C11A	C12A	C13A	O6A	2.7(4)
C11	C12	C13	C14	-174.4(3)		C11A	C12A	C13A	C14A	-176.7(3)
C11	C12	C21	C20	175.8(3)		C11A	C12A	C21A	C20A	177.0(3)
C11	C12	C21	C22	-3.5(4)		C11A	C12A	C21A	C22A	-1.4(4)
C12	C13	C14	C15	177.0(3)		C12A	C13A	C14A	C15A	-179.8(3)
C12	C13	C14	C19	-3.0(4)		C12A	C13A	C14A	C19A	-0.3(4)
C12	C21	C22	O9	-175.8(2)		C12A	C21A	C22A	O9A	176.4(2)
C12	C21	C22	C8	3.0(4)		C12A	C21A	C22A	C8A	-1.6(4)
C13	C12	C21	C20	-5.1(4)		C13A	C12A	C21A	C20A	-1.0(4)
C13	C12	C21	C22	175.6(3)		C13A	C12A	C21A	C22A	-179.4(3)
C13	C14	C15	C16	-179.2(3)		C13A	C14A	C15A	C16A	179.3(3)
C13	C14	C19	C18	178.8(3)		C13A	C14A	C19A	C18A	179.2(3)
C13	C14	C19	C20	-1.7(4)		C13A	C14A	C19A	C20A	-1.2(4)
C14	C15	C16	C17	-0.1(5)		C14A	C15A	C16A	C17A	0.7(5)
C14	C19	C20	O8	-177.5(3)		C14A	C19A	C20A	O8A	-177.8(3)
C14	C19	C20	C21	3.1(4)		C14A	C19A	C20A	C21A	1.6(4)
C15	C14	C19	C18	-1.2(4)		C15A	C14A	C19A	C18A	-1.3(4)
C15	C14	C19	C20	178.4(3)		C15A	C14A	C19A	C20A	178.4(3)
C15	C16	C17	C18	0.1(5)		C15A	C16A	C17A	C18A	0.4(5)
C16	C17	C18	O7	179.8(3)		C16A	C17A	C18A	O7A	177.6(3)
C17	C18	C19	C14	1.1(4)		C17A	C18A	C19A	C14A	2.3(4)
C17	C18	C19	C20	-178.5(3)		C17A	C18A	C19A	C20A	-177.3(3)
C18	C19	C20	O8	2.1(4)		C18A	C19A	C20A	O8A	1.8(4)
C18	C19	C20	C21	-177.3(3)		C18A	C19A	C20A	C21A	-178.8(3)
C19	C14	C15	C16	0.7(4)		C19A	C14A	C15A	C16A	-0.3(5)
C19	C20	C21	C12	0.3(4)		C19A	C20A	C21A	C12A	-0.4(4)
C19	C20	C21	C22	179.6(3)		C19A	C20A	C21A	C22A	177.9(3)
C20	C21	C22	O9	4.9(4)		C20A	C21A	C22A	O9A	-2.1(4)
C20	C21	C22	C8	-176.3(3)		C20A	C21A	C22A	C8A	179.9(3)
C21	C12	C13	O6	-172.4(3)		C21A	C12A	C13A	O6A	-179.2(3)
C21	C12	C13	C14	6.4(4)		C21A	C12A	C13A	C14A	1.3(4)
C22	O9	C5	C3	-174.2(2)		C22A	O9A	C5A	C3A	174.2(2)
C22	O9	C5	C6	4.3(4)		C22A	O9A	C5A	C6A	-5.9(4)
C22	C8	C9	C10	176.6(3)		C22A	C8A	C9A	C10A	169.6(3)
C22	C8	C9	C11	-3.8(4)		C22A	C8A	C9A	C11A	-5.4(4)

Table S8. Hydrogen atom coordinates (Å×10^4) and isotropic displacement parameters (Å^2×10^3) for actinomycetoquinone A (**1**).

**Atom**	***x***	***y***	***z***	**U(eq)**
H1	2960(70)	13400(40)	3190(40)	27
H2	6740(70)	14390(40)	4210(30)	19
H3	10850(60)	14760(40)	3570(40)	22
H5	7200(70)	6460(40)	5230(30)	20
H7	8190(60)	11080(40)	-590(40)	23
H1A	4751.44	11907.87	1669.08	26
H1B	5805.24	13001.03	1057.59	26
H2A	5111.2	14373.07	2388.69	18
H4A	8921.02	13308.11	1893.03	17
H4B	8587.7	14668.77	2070.88	17
H6	7526.17	12521.81	5134.77	19
H10A	8947.26	8564.01	5745.24	17
H10B	6615.65	8129.61	5537.85	17
H11	7490.87	7010.1	3329.47	16
H15	7227.43	6217.29	-543.1	20
H16	7504.57	6938.83	-2270.58	23
H17	7908.29	8991.29	-2549.05	21
H1AA	6930(70)	3400(40)	7060(40)	27
H2AA	960(70)	3460(40)	5890(30)	20
H3A	-1270(60)	1760(40)	6820(30)	20
H5A	420(70)	-3940(40)	4820(30)	21
H7A	2500(70)	260(40)	10730(40)	26
H1AB	5767.04	2063.85	8288.91	22
H1AC	4446.48	2680.16	9002.06	22
H2AB	4434.42	4303.55	7815.4	18
H4AA	1208.92	2085.56	8177.9	17
H4AB	1242.46	3510.4	8114.22	17
H6A	2932.55	1845.16	4945.26	20
H10C	1445.08	-2225.91	4247.43	20
H10D	3599.74	-2587.53	4468.63	20
H11A	2429.8	-3698.09	6607.51	18
H15A	2191.24	-4450.47	10426.97	23
H16A	2164.74	-3728.6	12186.34	26
H17A	2317.39	-1681.48	12551.9	23
H10E	5240(60)	4750(40)	5680(30)	26
H10F	3930(50)	5230(40)	5130(30)	26

Table S9. ^{1}H and ^{13}C NMR data for **2** (500 MHz for ^{1}H, 125 MHz for ^{13}C $CD_3OD:CDCl_3$ 1:1).

Positon	δ_C, mult.	δ_H (J in Hz)	^{1}H-^{1}H COSY	HMBC
1	62.9, CH_2	4.33, t (10.0);<br>4.21, dd (10.0, 3.3)	2	2, 3, 10'
2	70.8, CH	4.69, dd (10.0, 3.3);	1	1
3	78.7, C			
4	67.1, CH_2	4.08, d (12.0);<br>4.00, d (12.0)		3, 5
5	171.2, C			
6	112.1, CH	6.64, s		
7	180.8, C			
8	125.5, C			
9	151.3, C			
10	64.1, CH_2	4.89, d (17.0);<br>4.81, d (17.0)		9, 11
11	120.7, CH	7.86, s		8, 10, 13, 21
12	136.3, C			
13	182.2, C			
14	132.9, C			
15	119.8, CH	7.49, d (8.0)		13, 17, 19
16	137.0, CH	7.50, t (8.0)	17	14, 18
17	125.6, CH	7.16, d (8.0)	15, 16	15, 18
18	162.9, C			
19	117.4, C			
20	187.4, C			
21	120.4, C			
22	156.2, C			
2'	132.2, CH	6.98, s		3', 4', 9', 10'
3'	107.6, C			
4'	125.2, C			
5'	121.0, CH	7.26, d (8.0)	5'	3', 7', 9'
6'	122.1, CH	6.32, t (8.0)	4', 6'	4', 8'
7'	123.6, CH	6.56, t (8.0)	5', 7'	5', 9'
8'	112.5, CH	6.82, d (8.0)	6'	4', 6'
9'	136.6, C			
10'	165.6, C			

Table S10. ^{1}H and ^{13}C NMR data for **3** (600 MHz for ^{1}H, 150 MHz for ^{13}C DMSO-d_6).

Positon	δ_C, mult.	δ_H (J in Hz)	^{1}H-^{1}H COSY	HMBC
1	42.0, CH_2	2.87, dd (5.5, 2.5); 2.73, dd (5.5, 4.1)	2	2, 3
2	53.4, CH	3.66, dd (4.1, 2.5)	1	1, 3
3	74.4, C			
4	65.3, CH_2	4.22, dd (11.0, 6.0); 3.84, dd (11.0, 6.0)	4-OH	2, 3, 5
5	169.4, C			
6	111.2, CH	6.53, s		2, 3, 5, 7, 8, 9
7	178.2, C			
8	124.7, C			
9	153.4, C			
10	62.3, CH_2	5.15, br s		
11	118.8, CH	8.50, s		8, 10, 13, 20, 21, 22
12	136.0, C			
13	181.4, C			
14	132.1, C			
15	118.8, CH	7.68, d (7.5)		14
16	136.7, CH	7.76, t (7.5)	17	13, 17, 19
17	124.1, CH	7.38, d (7.5)	16	19
18	161.4, C			
19	116.7, C			
20	186.9, C			
21	119.8, C			
22	155.2, C			
3-OH		5.74, s		2, 3, 4, 5
4-OH		5.01, t (6.0)	4	3, 4
10-OH		5.71, t (5.1)		9, 10
18-OH		12.6, s		16, 17, 18, 19, 20

Table S11. ^{1}H and ^{13}C NMR data for **4** (600 MHz for ^{1}H, 150 MHz for ^{13}C CD_3OD:$CDCl_3$ 1:1).

Positon	δ_C, mult.	δ_H (J in Hz)	^{1}H-^{1}H COSY	HMBC
1	8.3, CH_3	0.92, t (7.4)	2	2, 3
2	34.1, CH_2	2.16, m; 1.96, m	1	1, 3, 4, 5
3	74.4, C			
4	26.9, CH_3	1.70, s		2, 3, 5
5	176.1, C			
6	109.8, CH	6.68, s		3, 5, 7, 8, 9
7	181.1, C			
8	125.6, C			
9	153.3, C			
10	64.3, CH_2	5.27, s	11	8, 9, 11
11	121.3, CH	8.61, s	10	8, 10, 13, 21
12	137.7, C			
13	182.6, C			
14	133.1, C			
15	120.1, CH	7.78, d (7.9)		13, 17, 19
16	137.3, CH	7.70, t (7.9)	17	14, 18
17	125.9, CH	7.34, d (7.9)	16	15, 18, 19
18	163.1, C			
19	117.5, C			
20	187.8, C			
21	121.3, C			
22	156.8, C			

Table S12. ^{1}H and ^{13}C NMR data for **5** (600 MHz for ^{1}H, 150 MHz for ^{13}C CD_3OD:$CDCl_3$ 1:1).

Positon	δ_C, mult.	δ_H (J in Hz)	^{1}H-^{1}H COSY	HMBC
1	117.4, CH_2	5.66, d (17); 5.36, d (11)	2	2, 3
2	137.4, CH	6.40, dd (17, 11)	1	3, 4, 5
3	78.4, C			
4	68.3, CH_2	4.36, d (12); 3.80, d (12)		2, 3, 5
5	172.4, C			
6	111.0, CH	6.74, s		3, 5, 7, 8
7	181.0, C			
8	125.8, C			
9	153.5, C			
10	64.3, CH_2	5.27, s	11	8, 9, 11
11	121.3, CH	8.62, s	10	8, 10, 13, 21
12	137.7, C			
13	182.6, C			
14	133.2, C			
15	120.2, CH	7.80, d (7.9)		13, 17, 19
16	137.4, CH	7.70, t (7.9)	17	14, 18
17	125.9, CH	7.36, d (7.9)	16	15, 18, 19
18	163.2, C			
19	117.5, C			
20	188.0, C			
21	121.3, C			
22	156.9, C			
